# Macrophage heterogeneity and oncogenic mechanisms in lung adenocarcinoma: insights from scRNA-seq analysis and predictive modeling

**DOI:** 10.3389/fimmu.2024.1491872

**Published:** 2025-01-09

**Authors:** Han Zhang, Jiaxing Dai, Qiuqiao Mu, Xiaojiang Zhao, Ziao Lin, Kai Wang, Meng Wang, Daqiang Sun

**Affiliations:** ^1^ Tianjin Chest Hospital, Tianjin University, Tianjin, China; ^2^ Tianjin Medical College, Tianjin, China; ^3^ Clinical School of Thoracic, Tianjin Medical University, Tianjin, China; ^4^ OmixScience Research Institute, OmixScience Co., Ltd., Hangzhou, China; ^5^ Liangzhu Laboratory, Zhejiang University, Hangzhou, China

**Keywords:** LUAD, TME, macrophage, prognostic model, immunotherapy, COL5A1

## Abstract

**Background:**

Macrophages play a dual role in the tumor microenvironment(TME), capable of secreting pro-inflammatory factors to combat tumors while also promoting tumor growth through angiogenesis and immune suppression. This study aims to explore the characteristics of macrophages in lung adenocarcinoma (LUAD) and establish a prognostic model based on macrophage-related genes.

**Method:**

We performed scRNA-seq analysis to investigate macrophage heterogeneity and their potential pseudotime evolutionary processes. Specifically, we used scRNA-seq data processing, intercellular communication analysis, pseudotime trajectory analysis, and transcription factor regulatory analysis to reveal the complexity of macrophage subpopulations. Data from The Cancer Genome Atlas (TCGA) was used to assess the impact of various macrophage subtypes on LUAD prognosis. Univariate Cox regression was applied to select prognostic-related genes from macrophage markers. We constructed a prognostic model using Lasso regression and multivariate Cox regression, categorizing LUAD patients into high and low-risk groups based on the median risk score. The model’s performance was validated across multiple external datasets. We also examined differences between high and low-risk groups in terms of pathway enrichment, mutation information, tumor microenvironment(TME), and immunotherapy efficacy. Finally, RT-PCR confirmed the expression of model genes in LUAD, and cellular experiments explored the carcinogenic mechanism of COL5A1.

**Results:**

We found that signals such as SPP1 and MIF were more active in tumor tissues, indicating potential oncogenic roles of macrophages. Using macrophage marker genes, we developed a robust prognostic model for LUAD that effectively predicts prognosis and immunotherapy efficacy. A nomogram was constructed to predict LUAD prognosis based on the model’s risk score and other clinical features. Differences between high and low-risk groups in terms of TME, enrichment analysis, mutational landscape, and immunotherapy efficacy were systematically analyzed. RT-PCR and cellular experiments supported the oncogenic role of COL5A1.

**Conclusion:**

Our study identified potential oncogenic mechanisms of macrophages and their impact on LUAD prognosis. We developed a prognostic model based on macrophage marker genes, demonstrating strong performance in predicting prognosis and immunotherapy efficacy. Finally, cellular experiments suggested COL5A1 as a potential therapeutic target for LUAD.

## 1 Introduction

Lung cancer, particularly non-small cell lung cancer (NSCLC) and its major subtype LUAD, has consistently been one of the most commonly diagnosed cancers worldwide and a leading cause of cancer-related deaths (1). Over the years, the treatment of lung cancer has evolved to include surgical interventions, radiation therapy, chemotherapy, targeted therapy, and immunotherapy (2). Specifically, targeted therapies focusing on specific molecular alterations and immunotherapies that harness the immune system to combat cancer cells herald a new era of personalized medicine, leading to improved survival rates and reduced side effects for many patients (2, 3). However, significant challenges persist due to the heterogeneity of lung cancer, the emergence of treatment-resistant mutations, and the complex interactions between the tumor and its microenvironment (4). Furthermore, the invasiveness of the disease and its prognostic implications remain substantial barriers, with many individuals still unable to be accurately detected and prognosticated early (5, 6). The dynamic landscape of lung cancer research underscores the ongoing need for innovation, integration of emerging technologies, and multidisciplinary collaboration to overcome these challenges and improve patient prognoses.

TME constitutes a complex and diverse network, not only composed of tumor cells but also encompassing various stromal elements, including immune cells, fibroblasts, endothelial cells, and the extracellular matrix (7). This ecosystem plays a crucial regulatory role in tumor initiation, progression, metastasis, and treatment response. With a deepening understanding of tumor biology, immune-centric therapeutic strategies such as Chimeric Antigen Receptor T-cells (CAR-T), CAR Natural Killer cells (CAR-NK), and emerging CAR Macrophages (CAR-M) have garnered widespread attention (8–10). These treatments utilize the power of the immune system to target and eradicate specific malignancies with unprecedented precision. However, different factors in the TME may influence these innovative therapies. Tumor-associated macrophages (TAMs), depending on their polarization states, can either promote tumor growth or enhance tumor immune responses (11). Therefore, it becomes particularly important to consider a comprehensive strategy in cancer treatment, one that recognizes the immense potential of new immunotherapies while also considering the complexity of the TME, especially in the interaction between key components like TAMs and advanced treatments like CAR-T, CAR-NK, CAR-M.

scRNA-seq sequencing technology has shown significant advantages in cancer research. It reveals the intricate heterogeneity within tumor cells, providing deep insights into tumor development, progression, and treatment response (12). This technology is particularly applicable for analyzing intercellular interactions within the TME, helping researchers understand the relationships between tumor cells and surrounding immune and other cell types. Moreover, scRNA-seq sequencing can reveal mechanisms of tumor treatment resistance, providing key information for developing personalized treatment strategies. Combining scRNA-seq sequencing with traditional Bulk-RNA sequencing to establish a prognostic model for LUAD captures the tumor’s biological characteristics more comprehensively. This integrated approach not only reveals the overall characteristics of tumor cell populations but also precisely locates changes at the scRNA-seq level. Such a strategy greatly enhances the accuracy and clinical utility of the model, making prognostication more precise, thereby guiding more effective treatment decisions and ultimately improving treatment outcomes and quality of life for LUAD patients.

In this study, we first analyzed macrophage heterogeneity in scRNA-seq, identifying SPP1 and MIF as potential oncogenic pathways in macrophages through the ‘CellChat’ R package. Then we examined the impact of different macrophage subtypes on LUAD prognosis and established a robust prognostic model based on macrophage marker genes. We found that patients in the low-risk group had better prognoses, more immune cell infiltration, and were more likely to benefit from immunotherapy. Additionally, a series of basic experiments corroborated the accuracy of our analysis and identified COL5A1 as a potential therapeutic target for LUAD.

## 2 Methods

### 2.1 Data acquisition

The scRNA-seq dataset (GSE131907) used for the analysis of macrophage heterogeneity was downloaded from the Gene Expression Omnibus (GEO) database (https://www.ncbi.nlm.nih.gov/geo/query/acc.cgi?acc=GSE131907) (12). This scRNA-seq dataset comprises 58 samples, including 21 normal samples (11 normal lung tissues and 10 normal lymph node samples), and 37 tumor samples (18 LUAD tumor tissues, 10 LUAD brain metastases, 7 LUAD metastatic lymph node tissues, and 5 LUAD pleural effusion metastases). The gene expression matrix, clinical information, and mutation data of LUAD patients used for training the prognostic model were downloaded from The Cancer Genome Atlas (TCGA) database (https://portal.gdc.cancer.gov/repository). Gene expression matrices and clinical information for five independent validation cohorts (GSE31210, GSE37745, GSE50081, GSE68465, GSE3141) used for model prognosis and diagnostic capability validation were obtained from the GEO database. The expression matrix and clinical information for the bladder cancer immunotherapy cohort IMVigor210, treated with anti-PD-1 therapy, were downloaded from the website provided in previous literature (http://researchpub.gene.com/IMvigor210CoreBiologies) (13). The Immunophenoscoring (IPS) data, used for predicting immune therapy responses, were downloaded from The Cancer Immunome Atlas (TCIA) database (https://tcia.at/patients) (14). A higher IPS indicates a higher likelihood of the patient benefiting from immunotherapy. Additionally, we downloaded related data from the TIDE (http://tide.dfci.harvard.edu) database to evaluate the likelihood of immune escape in LUAD patients. The melanoma cohort GSE78220, post-immunotherapy, was downloaded from the GEO database (15).

### 2.2 scRNA-seq data analysis

The analysis of scRNA-seq data was primarily conducted using the R packages “Seurat”, “SCTransform”, “SCP”, and “CellChat”. Initially, the raw matrix files were loaded into the R environment to create Seurat objects. Rigorous quality control was applied, retaining cells with gene expression levels between 300 and 10,000, mitochondrial gene proportions below 20%, and erythrocyte gene proportions below 3%. Genes expressed at levels below 3 in individual cells were removed, resulting in 207,626 high-quality cells. The CellCycleScoring function was used to assess the cell cycle, and the SCTransform function for data scaling and normalization. Principal Component Analysis (PCA) based on 3,000 highly variable genes was used for dimensionality reduction, and the “Harmony” R package was employed to eliminate batch effects between different samples. Using the “FindNeighbors” and “FindClusters” functions, we identified 40 cell clusters. A combination of manual annotation and the “SingleR” package was used for final cell sub-group annotation, resulting in 8 distinct cell subgroups. The “CellChat” R package was utilized to infer communication between cell subgroups. The “SCP” package’s RunSlingshot function was used for pseudotime analysis of four macrophage types. The “FindAllmarkers” function was employed to calculate marker genes for each macrophage type. Simultaneously, we inferred potential gene regulatory networks for four types of macrophages based on the ‘SCENIC’ R package.

We used the ‘inferCNV’ algorithm to infer copy number variation (CNV) in epithelial cells to assess their malignant characteristics. First, the raw count matrix of epithelial cells was extracted from single-cell RNA sequencing data, and cells were annotated based on tissue type, with tumor samples labeled as “malignant_Epithelial” and normal samples as “normal.” The ‘inferCNV’ package was then used to create an analysis object by inputting the gene expression matrix, cell annotation file, and gene order file. During the analysis, the normal cell group was used as a reference for CNV inference, followed by denoising and clustering. The results and associated plots were generated. CNV features of tumor and normal cells were compared based on the inferred CNV data. Subsequently, the CNV value for each cell was calculated, and Pearson correlation analysis was performed to correlate tumor cells with the top 5% of cells exhibiting the highest CNV. Based on these analyses, each cell was assigned a “cancer” or “normal” label. Finally, a scatter plot was created using ‘ggplot2’ to illustrate the correlation between CNV values and tumor cells, further evaluating the identification and distribution of malignant cells.

### 2.3 Evaluation of the prognostic impact of macrophage enrichment scores in LUAD patients

In the TCGA database, macrophage enrichment scores for each individual sample were computed using the Single-sample GSEA (ssGSEA) algorithm (16), based on specific macrophage marker genes. Initially, a comparative analysis was conducted to examine the disparities in enrichment scores between normal and tumor samples. Following this, the tumor samples from TCGA-LUAD were bifurcated into two distinct groups according to the median of their enrichment scores. Subsequently, a Kaplan-Meier survival analysis was undertaken to elucidate the survival variances between these groups.

### 2.4 Construction and validation of the prognostic model

The process commenced with a correlation analysis to sift out genes in the TCGA database that exhibited a correlation exceeding 0.4 with the macrophage enrichment scores. These genes were then subjected to a univariate COX analysis, pinpointing those with a significant bearing on the survival of LUAD patients. Following this, Lasso regression analysis was deployed to refine the gene pool, culminating in the development of a multivariate COX regression-based prognostic model for LUAD, centered on macrophage-related marker genes. Based on the model’s scoring system, patients were categorized into high and low-risk groups. The Kaplan-Meier method was employed to craft survival curves, while Log-rank tests were utilized to evaluate the survival disparities. Moreover, the model’s capacity to diagnose 1-, 3-, and 5-year survival rates was gauged using the receiver operating characteristic (ROC) curve function of the R package ‘TimerROC’ (17, 18).

### 2.5 Nomogram development

The initial step involved performing univariate and multivariate COX analyses on the risk scores and clinical data of TCGA-LUAD patients, aiming to identify variables with significant prognostic influence on LUAD. Leveraging the ‘rms’ R package (19), the final nomogram was constructed. Its efficacy was subsequently appraised using calibration curves and decision curve analysis (DCA).

### 2.6 Enrichment analysis

The analysis began with the application of the ‘GSVA’ algorithm (20) to discern Hallmarker pathways that were significantly more enriched in the high-risk group as opposed to the low-risk group. This was followed by employing the ‘GSEA’ algorithm (21) to dissect the significant Gene Ontology (GO) pathway differences between the high and low-risk groups (22). The ssGSEA algorithm facilitated an assessment of the correlation between enrichment scores and tumor immunity-related scores, as well as pathways pertinent to immunotherapy efficacy (**Supplementary Table 1**). Furthermore, this algorithm was instrumental in quantifying the divergences in immune cells and immune-related functions between the high and low-risk groups. Lastly, the enrichment of genes involved in model construction within the scRNA-seq data was evaluated using the ‘AUcell’ R package (23).

### 2.7 Tumor microenvironment assessment

Immune cell infiltration data for seven databases were downloaded from the Timer2.0 database (http://timer.comp-genomics.org/) (24). Subsequently, the differences in immune cell infiltration between high and low-risk groups were assessed. The ‘estimate’ R package (25) was used to calculate stromal, immune, TumorPurity, and ESTIMATE scores for each TCGA-LUAD sample.

### 2.8 Mutation analysis

Mutation data for LUAD patients were downloaded from the TCGA database using the R package ‘TCGAbiolinks’, and uniformly decompressed. The ‘maftools’ R package’s read.maf function was employed to read mutation data and clinical information into a maf file. The plotmafSummary function analyzed the mutation profile of TCGA-LUAD patients. The oncoplot function generated heatmaps combining clinical and mutation information to display mutation details in high and low-risk groups. The somaticInteractions function was used to analyze co-mutation scenarios between hub genes and the top 10 most frequently mutated genes in TCGA-LUAD. Additionally, Tumor Mutation Burden (TMB) was calculated for each patient, comparing the differences in TMB between high and low-risk groups and the correlation between risk scores and TMB.

### 2.9 Immunotherapy efficacy prediction

Initially, differences in the expression of immune checkpoint-related genes and Major Histocompatibility Complex (MHC) genes between high and low-risk groups were compared, and the correlations between hub genes, risk scores, and the aforementioned genes were calculated. Subsequently, differences in Immunophenoscoring (IPS) between high and low-risk groups were calculated, along with the use of The tumor immune dysfunction and exclusion (TIDE) algorithm to assess the likelihood of immune escape (26). Finally, the prognostic and immunotherapy predictive capabilities of the model were further validated in two immunotherapy cohorts, IMvigor210 and GSE78220.

### 2.10 Cell line culture

In our experimental protocol, the BEAS-2B normal human lung epithelial cells and LUAD cell lines (A549, H1650,H1975,H1299) were obtained from the Cell Resource Center of the Shanghai Institute for Biological Sciences. Subsequently, they were cultured in RPMI-1640 medium (Gibco BRL, USA), supplemented with 10% fetal bovine serum (FBS, Cell-Box, Hong Kong) and 1% penicillin-streptomycin mixture (Biosharp, China). The culture conditions were maintained at 5% CO_2_, 95% relative humidity, and a constant temperature of 37°C.

### 2.11 RNA extraction and RT-PCR analysis

Total RNA was extracted from the cell lines using TRIzol (15596018, Thermo) as per the provided protocol. cDNA synthesis was performed with the PrimeScriptT-MRT kit (R232-01, Vazyme). The subsequent quantitative RT-PCR was conducted using SYBR Green Master Mix (Q111-02, Vazyme), with GAPDH mRNA serving as the normalization control. The relative expression levels were calculated employing the 2−ΔΔCt method. Primers used were sourced from Nanjing Sunbio Technology Co.,Ltd (Nanjing, China), detailed in **Supplementary Table 1**.

### 2.12 Cell growth evaluation via CCK-8 assay

Cells were seeded at 3×10³ cells/well in 96-well plates. Post-seeding, 10 mL of CCK-8 solution (A311-01, Vazyme) was added, and cells were incubated at 37°C in the dark for 2 hours. Cell proliferation was monitored by measuring absorbance at 450 nm at 0, 24, 48, 72, and 96 hours using a spectrophotometer (A33978, Thermo).

### 2.13 Assessment of colony formation

Cells were plated at 1×10³ cells/well in 6-well plates and cultured for 14 days. Post-incubation, cells were washed with PBS, fixed with 4% paraformaldehyde for 15 minutes, and stained with Crystal Violet from Solarbio, China.

### 2.15 Migration and invasion analysis via transwell assays

Migration and invasion capacities were tested using 24-well transwell inserts, with A549 and H1650 cells seeded at 1×10^5^ cells in the upper chamber. For invasion assays, chambers were pre-coated with matrigel (BD Biosciences, USA), while others remained uncoated for migration assays. Post-migration/invasion, cells on the membrane’s underside were fixed and stained with crystal violet (Solarbio, China).

### 2.16 Animal models

The subcutaneous tumor formation animal experiment was conducted after obtaining approval from the Animal Experiment Ethics Committee of Tianjin Chest Hospital. We implanted A549 cells, stably transfected with COL5A1, and untreated control cells into the left and right inguinal areas of 5-week-old BALB/c nude mice, respectively. After 30 days of cultivation, the mice were euthanized, and the tumors were collected for weighing.

### 2.17 Macrophage co-culture

In this study, we employed a co-culture experimental approach to explore the interactions between macrophages and LUAD cell lines. Specifically, we first treated THP-1 cells with 200 nM PMA for 24 hours to induce their differentiation into macrophages. These differentiated macrophages were then co-cultured with two different states of LUAD cell lines: one untreated and the other with knocked-down COL5A1 expression. After the co-culture, we utilized RT-PCR technology to detect the expression of anti-inflammatory Mφ markers CD163 and CD206 in the macrophages, assessing their polarization status under varying microenvironmental conditions. This approach allowed us to gain a deeper understanding of the role of COL5A1 in regulating macrophage behavior and LUAD development, thereby providing a foundation for revealing the potential value of COL5A1 as a therapeutic target.

### 2.18 Statistical analysis

The statistical analyses for the bioinformatics part were conducted in the R environment (version 4.3.1), while basic experimental data were statistically analyzed using Graphpad and ImageJ. When evaluating intergroup differences, the independent sample T-test or one-way ANOVA was applied for samples adhering to a normal distribution. For samples not following a normal distribution, the Wilcoxon rank-sum test or Kruskal-Wallis test was employed. Survival analysis was performed using the Kaplan-Meier method to construct survival curves, with Log-rank tests assessing the survival differences. Spearman’s Rank Correlation Coefficient was utilized to determine correlations between data sets. A p-value of less than 0.05 was considered statistically significant, with *P < 0.05, **P < 0.01, ***P < 0.001 indicating increasing levels of significance.

## 3 Results

### 3.1 Establishment of the scRNA-seq Atlas of LUAD

**Figure 1:**
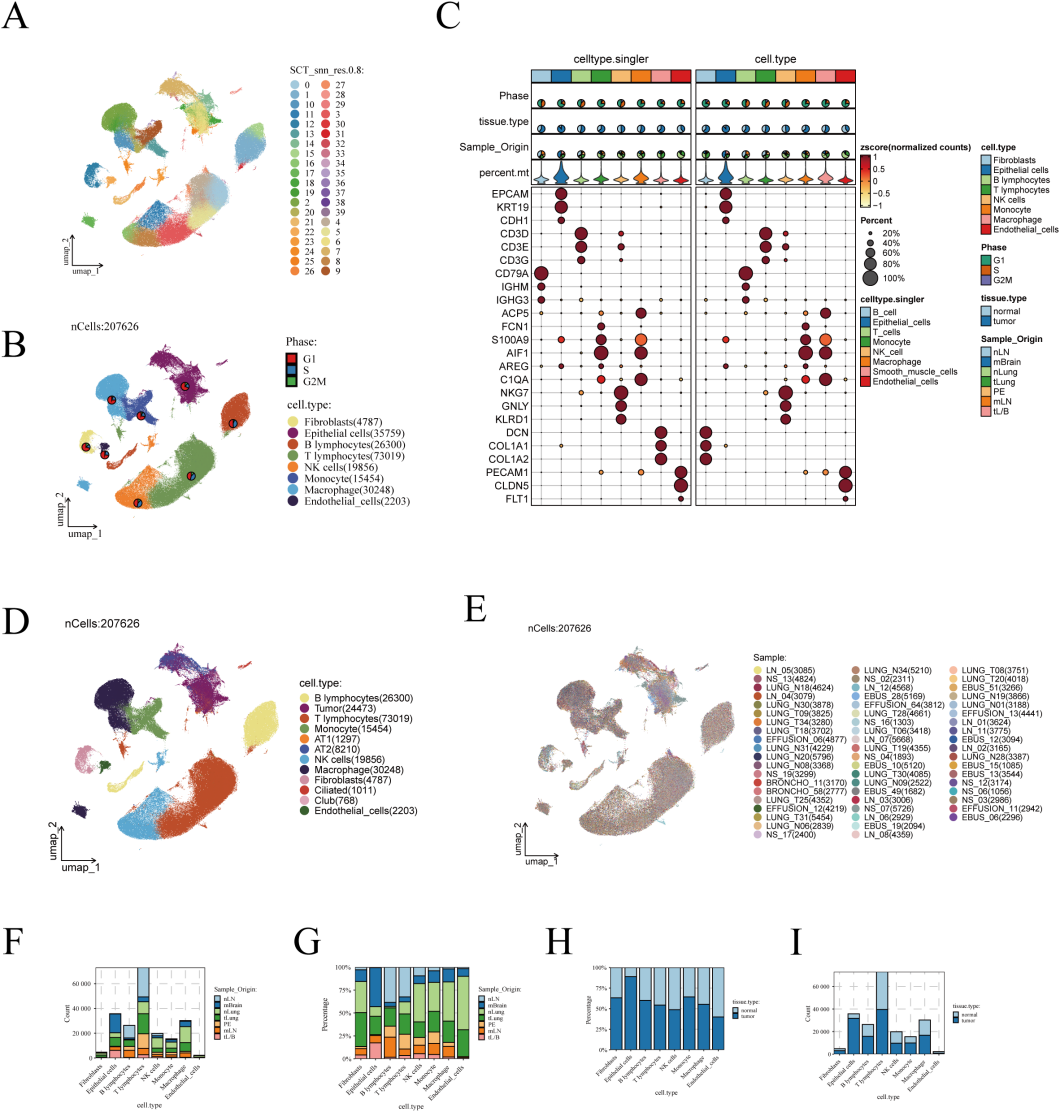
Visualization of scRNA-seq data. **(A)** The UMAP algorithm identified 40 cell clusters. **(B)** Eight cell types were annotated using the UMAP algorithm based on marker genes. **(C)** Display of marker genes, with the left half showing results annotated by SingleR and the right half showing manually annotated results. **(D)** UMAP plot showing the distribution of cells, with malignant epithelial cells inferred using the inferCNV algorithm and further clustering of benign epithelial cells. **(E)** UMAP algorithm showing the patient origin of cells. **(F)** and proportion **(G)** of cells from different tissue origins. Bar graphs illustrating the differences in cell numbers **(H)** and proportions **(I)** between LUAD and normal lung tissues.

As detailed in the Methodology section and illustrated in **Supplementary Figure 1**, through stringent quality control, we obtained 207,626 high-quality cells from 58 samples across 44 patients. Utilizing the ‘Seurat’ and ‘SCTransform’ packages, 40 distinct cell clusters were identified using UMAP (Uniform Manifold Approximation and Projection) technology (**Figure 1A**). The study combined manual annotation (based on classic marker genes) and automatic annotation via the SingR algorithm, successfully categorizing these cell clusters into 8 different subgroups (**Figure 1B**). Additionally, the expression of these marker genes is depicted in a bubble chart format in **Figure 1C**.

Next, we isolated epithelial cells and applied the inferCNV algorithm to infer the malignancy of tumor-derived epithelial cells. As shown in **Supplementary Figure 2A**, epithelial cells were classified into 35 distinct clusters. Using the inferCNV algorithm, chromosomal variations in normal tissue-derived epithelial cells were used as a reference to infer the malignancy of tumor-derived epithelial cells (**Supplementary Figure 2B**). Malignant epithelial cells were identified based on a Pearson correlation coefficient greater than 0.2 between their CNV profiles and the average CNV features of the top 5% tumor cells, with a CNV level exceeding 0.2 (**Supplementary Figure 2C**). The inferCNV annotation results are shown in **Supplementary Figure 2D**, and the tissue origin of the epithelial cells is depicted in **Supplementary Figure 2E**. Overall, the number of malignant cells inferred by the inferCNV algorithm was lower than the number of tumor-derived epithelial cells, which we attribute to the presence of normal tissue-derived cells mixed within the tumor samples during collection.

We further evaluated the proportion of malignant versus benign cells in each cluster (**Supplementary Figure 2F**). Clusters with a higher proportion of malignant cells were annotated as tumor cells, while clusters with a higher proportion of normal cells were classified as normal lung epithelial cells. Normal cells were further annotated based on marker gene expression, identifying them as AT1, AT2, ciliated, or club cells (**Supplementary Figure 2G**). The expression patterns of lung epithelial marker genes are shown in **Supplementary Figure 2H**, confirming the accuracy of the clustering. The epithelial cell annotations derived from the inferCNV algorithm were then incorporated into the metadata and visualized (**Figure 1D**). Additionally, we examined the distribution of cells according to their sample origins, observing an even distribution across the different cell types with no apparent batch effects (**Figure 1E**).

We further analyzed the distribution of different cell subgroups across various tissue sources. The analysis indicated that T cells are the most abundant in the TME, followed by epithelial cells and macrophages (**Figure 1F**). Notably, macrophages were more abundant in normal lung tissues compared to early-stage LUAD tissues (**Figure 1G**). When comparing cell abundance between tumor and normal lung tissues, it was found that epithelial cells predominantly reside in tumor tissues (**Figure 1H**). In contrast, the distribution of macrophages showed less disparity between normal and tumor tissues (**Figure 1I**).

### 3.2 Intercellular communication analysis

**Figure 2:**
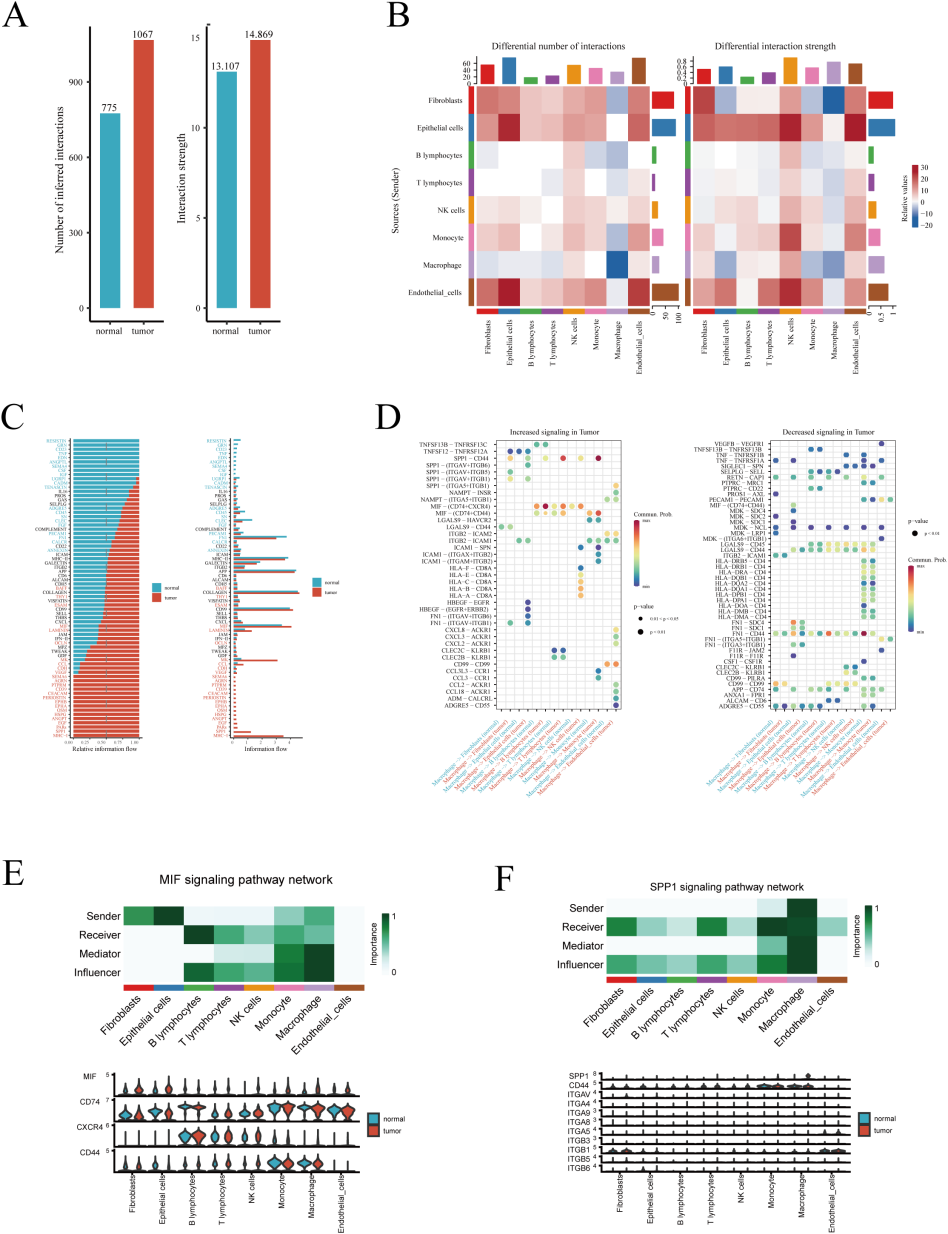
Intercellular communication analysis. **(A)** Overview of the differences in the number and intensity of signaling pathways between tumor and normal samples. **(B)** Heatmap showing the cellular origins of differing signaling pathways between tumor and normal samples. **(C)** Bar graph detailing the specific signaling pathways that differ. **(D)** Heatmap displaying significantly increased and decreased macrophage-related signaling pathways in tumor samples. **(E)** The role of the MIF and **(F)** SPP1 signaling pathways among different cells, and the expression of key genes in these pathways.

We performed intercellular communication analysis on tumor and normal lung tissues separately using the ‘CellChat’ R package, aiming to identify signaling pathways that are aberrantly activated or inactivated in tumors, thereby potentially identifying therapeutic targets for LUAD. Initially, at an overarching level, signaling pathways in tumors were more active compared to normal tissues, both in terms of the number and intensity of signals (**Figure 2A**). Heatmaps of interaction quantity and intensity revealed an increase in both the number and strength of interactions involving epithelial cells, whether as target or source cells, in tumor tissues (**Figure 2B**). Conversely, signaling pathways related to macrophages were significantly reduced, an intriguing phenomenon that may reflect how alterations in the tumor microenvironment are associated with the inhibition of certain normal functions and interactions of macrophages. This suggests that modulating these pathways could offer potential strategies for cancer treatment. Furthermore, we compared all significantly different signaling pathways between tumor and normal samples in detail, identifying pathways such as SPP1 and MHC-I as particularly active in tumors (**Figure 2C**). Detailed analysis of macrophage-related signaling pathways revealed an increase in the activity of SPP1 and MIF pathways in tumors, potentially related to macrophages promoting tumor growth, invasion, or immune suppression (**Figure 2D**, left). In contrast, the activity of pathways from macrophages, such as VEGFB to VEGFR1 and TNFSF13B to TNFRSF13B, was reduced in tumors, possibly reflecting adaptive changes in macrophage function under tumor conditions, which could impact immune responses and tumor cell regulation (**Figure 2D**, right). Despite an overall suppression of macrophage-related pathways (**Figure 2B**), the activities of SPP1 and MIF remained more vigorous, potentially indicating a closer association of these pathways with cancer progression. Therefore, we presented detailed information on these two pathways and compared the expression of key molecules in these pathways between tumor and normal lung tissues (**Figures 2E, F**). Results showed that MIF is primarily produced by macrophages and acts on fibroblasts, epithelial cells, and other immune cells, whereas the SPP1 pathway is highly related to macrophages.

### 3.3 Pseudotime analysis and transcription factor regulatory analysis of macrophages

**Figure 3:**
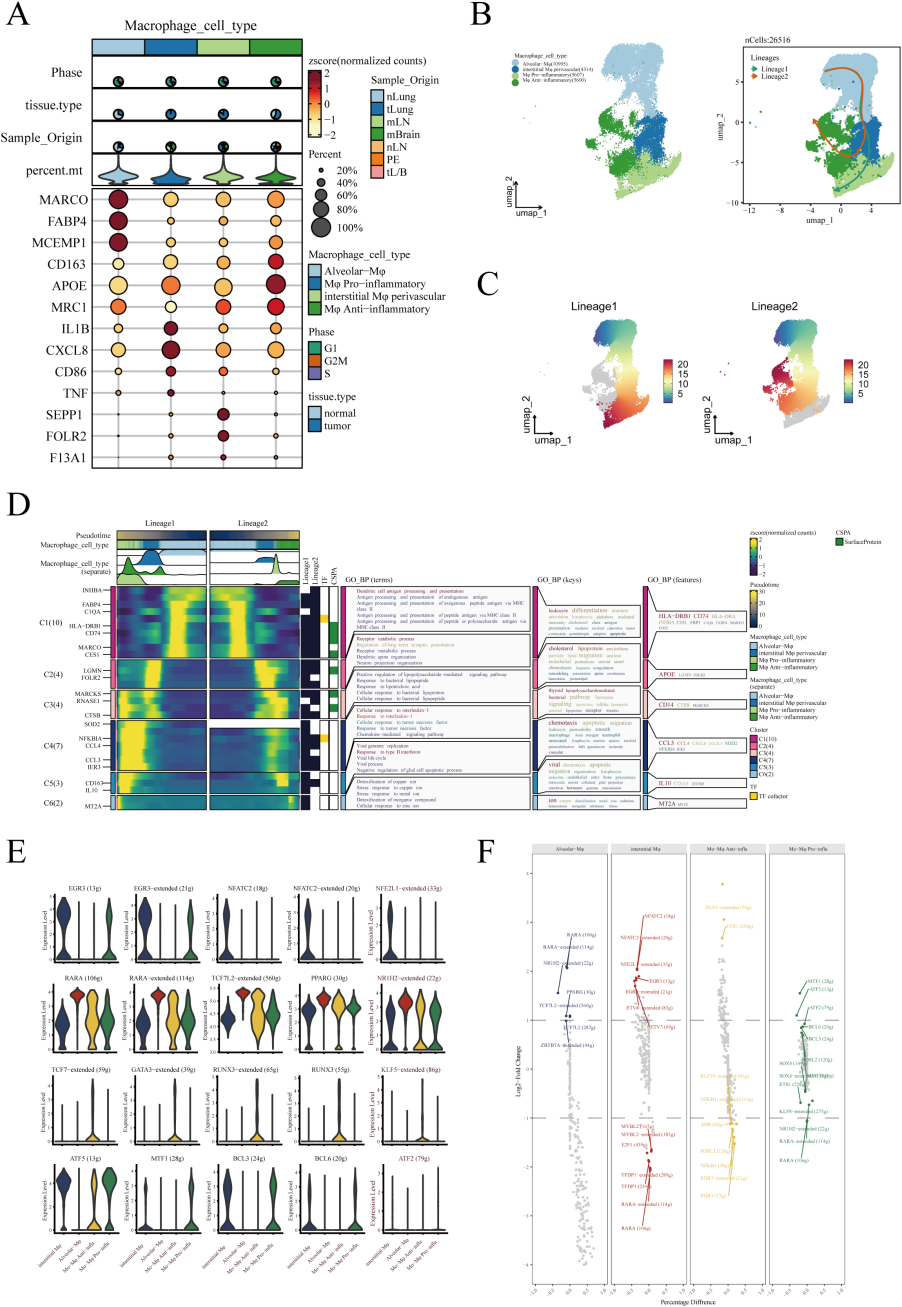
Identification and pseudotime analysis of macrophage subgroups. **(A)** Marker genes for four types of macrophages. **(B)** Four types of macrophages annotated in the UMAP algorithm based on marker genes and pseudotime analysis conducted using the Slingshot R package, revealing two distinct developmental trajectories of macrophages. **(C)** Detailed illustration of the two differentiation trajectories, where the depth of color indicates the order in pseudotime, with darker colors representing later stages. **(D)** Heatmap depicting the differential gene expression during the differentiation process of the two cell fates and the enrichment analysis results of these genes. **(E)** Violin plots show the top 5 transcription factors most strongly associated with each type of macrophage. **(F)** The top 7 transcription factors with the highest and lowest expression in each type of macrophage.

In order to delve deeper into the subtypes and functions of macrophages within the TME, we conducted a detailed sub-group analysis and pseudotime trajectory study. Drawing from existing literature, we classified macrophages into four subtypes: Alveolar−Mφ, Interstitial Mφ Perivascular, Mφ Anti−inflammatory, and Mφ Pro−inflammatory (27–30). **Figure 3A** displays the expression of marker genes within each macrophage subgroup. The UMAP dimensionality reduction method was utilized to illustrate the distribution of these macrophage subgroups in the reduced space, underscoring their separation and specificity (**Figure 3B**). Next, employing the Slingshot R package for pseudotime analysis, we discovered two distinct trajectories of macrophage fate (**Figure 3B**). Both differentiation pathways initiated from Alveolar−Mφ, proceeded through Interstitial Mφ Perivascular, and finally diverged into either Mφ Anti−inflammatory or Mφ Pro−inflammatory. Additionally, we meticulously depicted these two differentiation pathways, with varying shades representing the order in pseudotime (**Figure 3C**). Next, we analyzed the differential gene expression and their enrichment in biological pathways during the differentiation process of these two divergent macrophage fates. We found that pathways related to dendritic cell antigen processing and presentation were predominantly enriched in Alveolar Mφ, while Interstitial Mφ Perivascular were associated with positive regulation mediated by lipopolysaccharides. Moreover, high expression of CD163 and CCL2 in anti-inflammatory macrophages may indicate their key role in anti-inflammatory responses (**Figure 3D**). To gain insight into the transcriptional regulation of macrophages, we analyzed the activity and expression of transcription factors across different macrophage populations. As shown in **Figure 3E**, we present the top five transcription factors with the highest expression levels across four distinct macrophage subtypes using violin plots. These plots provide a visual representation of the distribution and variation in transcription factor expression within each group. In **Figure 3F**, we further explore the transcriptional landscape by highlighting the seven transcription factors with the highest and lowest expression levels in various macrophage subtypes. This comparative analysis allows us to identify key regulators that may play a significant role in macrophage function and their involvement in immune responses, inflammation, and the tumor microenvironment.

### 3.4 Macrophage subgroups’ impact on LUAD prognosis

**Figure 4:**
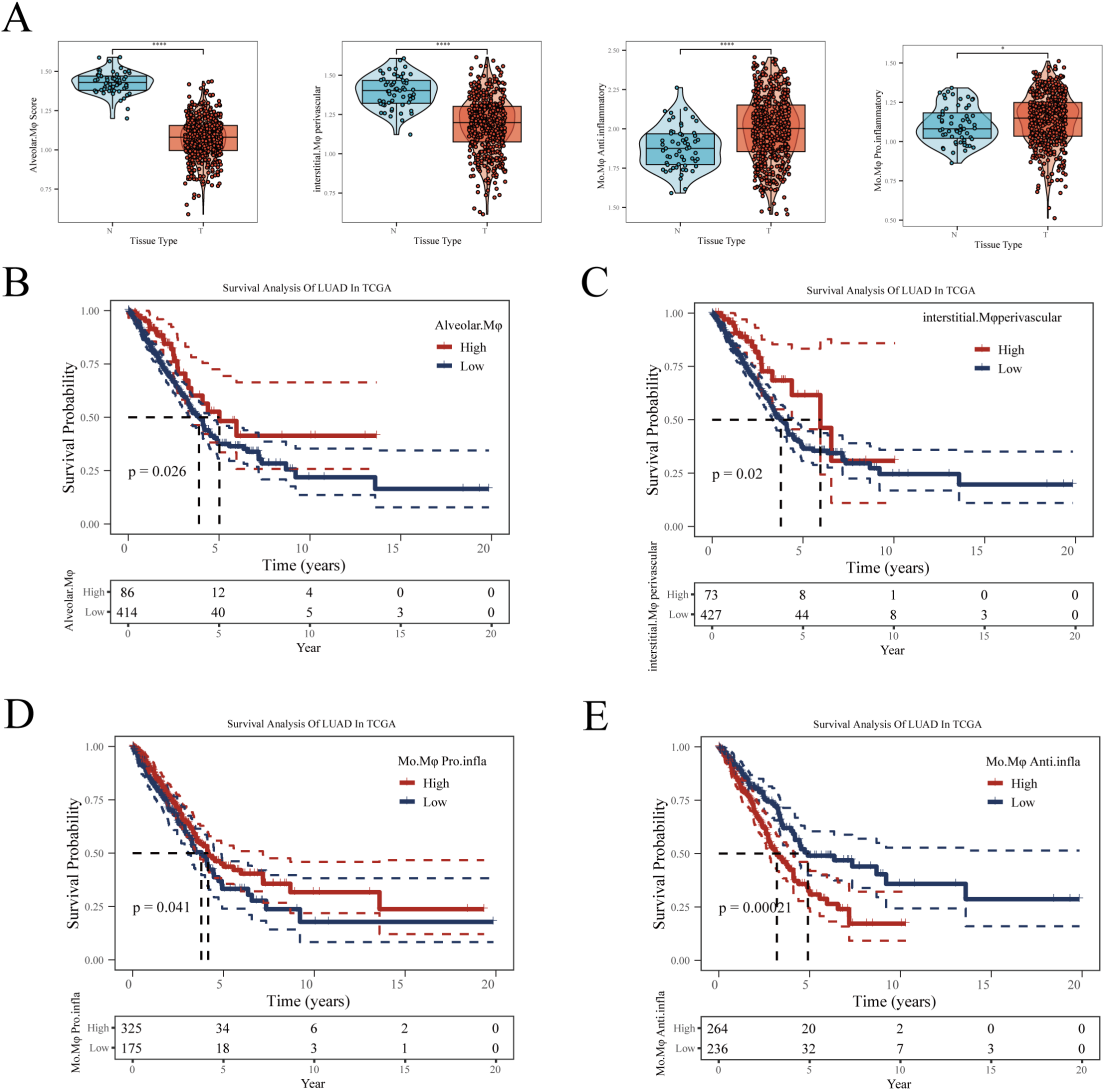
The relationship between four types of macrophages and LUAD prognosis. **(A)** Differences in enrichment scores of four types of macrophages between normal and tumor samples in TCGA-LUAD. **(B)** Impact of Alveolar-Mφ, **(C)** Interstitial-Mφ Perivascular, **(D)** Mo-Mφ Pro-Inflammatory, **(E)** Mo-Mφ Anti-Inflammatory enrichment scores on LUAD prognosis. The optimal cut-off value was determined using the surv_cutpoint function from the ‘survminer’ package. *P < 0.05; **P < 0.01; ***P < 0.001.

We next explored the potential impact of different macrophage subgroups on the prognosis of lung adenocarcinoma (LUAD) patients. Initially, marker genes for various macrophages were identified in sc-RNAseq data using the FindAllMarkers function. In the TCGA-LUAD cohort, we then calculated the enrichment scores for each patient’s macrophage subgroups using the ssGSEA algorithm based on these marker genes. Comparing the macrophage enrichment scores between normal lung tissues and tumor tissues (**Figure 4A**), we observed that the enrichment scores of Alveolar-Mφ, Interstitial-Mφ Perivascular, and Mφ Pro-inflammatory were significantly higher in normal samples than in tumor samples. Conversely, Mφ Anti-inflammatory was notably more enriched in tumor tissues, suggesting a potential association of this macrophage subgroup with poor prognosis in LUAD. Further Kaplan-Meier survival analysis revealed that the three macrophage subgroups significantly enriched in normal samples correlated with better prognosis in LUAD patients (**Figures 4B–D**). Specifically, higher enrichment scores of these subgroups in patients were associated with longer overall survival, indicating their potential role in inhibiting tumor progression. On the other hand, patients with higher enrichment scores of Mφ Anti-inflammatory exhibited poorer prognosis (**Figure 4E**), suggesting that this subgroup might contribute to tumor growth and immune escape. In summary, all four macrophage subgroups significantly influenced the prognosis of LUAD, leading us to include all macrophage marker genes in our subsequent modeling.

### 3.5 Construction and validation of the prognostic model

**Figure 5:**
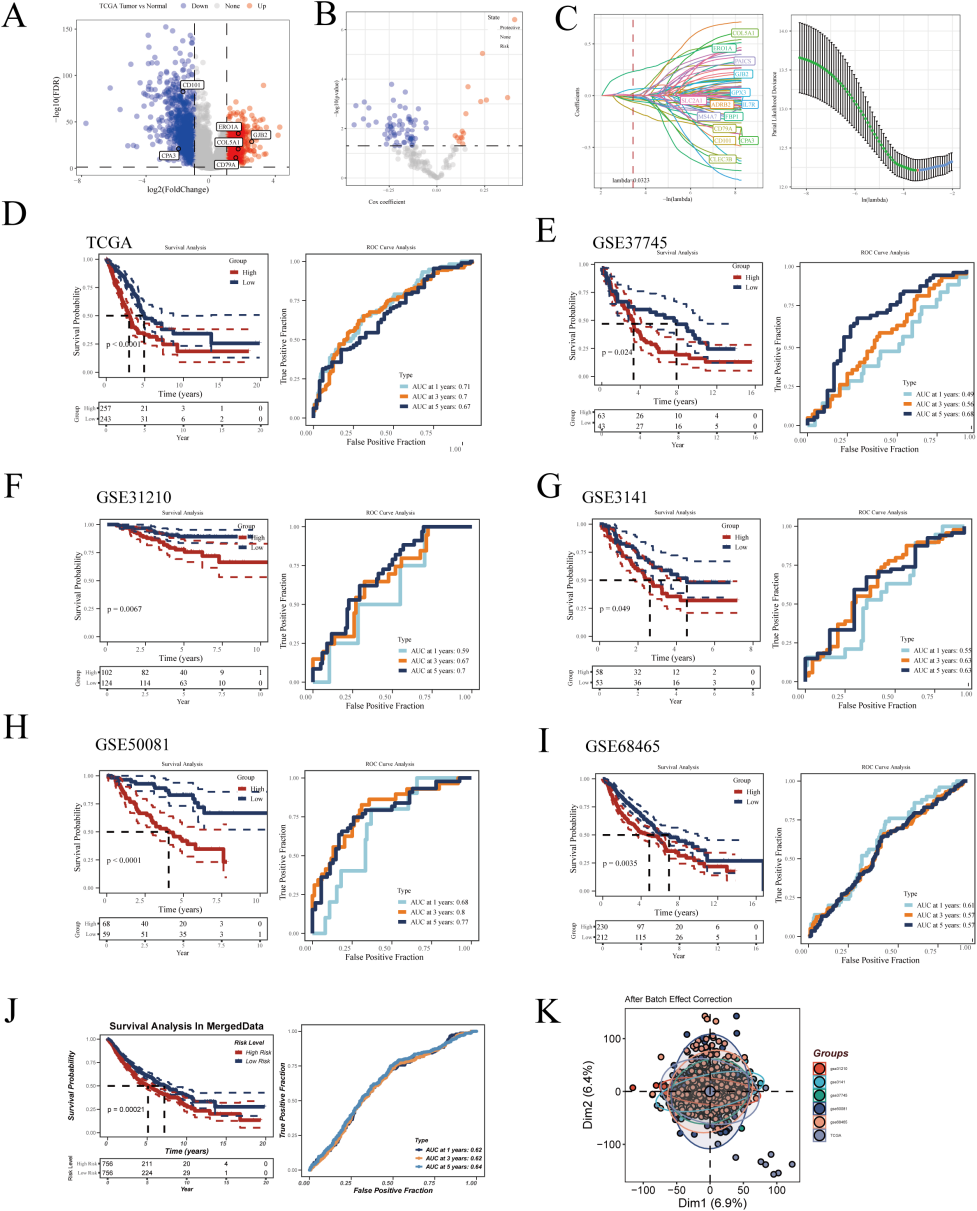
Construction of the LUAD prognostic model. **(A)** Volcano plot displaying differential genes between tumor and normal samples in TCGA-LUAD. **(B)** Volcano plot showing results of univariate Cox regression analysis. **(C)** Lasso regression further refines LUAD prognosis-related genes. Kaplan-Meier survival curves and ROC curves demonstrating the model’s prognostic and diagnostic efficacy in **(D)** TCGA-LUAD, **(E)** GSE37745, **(F)** GSE31210, **(G)** GSE3141, **(H)** GSE50081, **(I)** GSE68465, and **(J)** MergeData. **(K)** Principal component analysis (PCA) plot of six LUAD datasets after batch effect removal.

Developing a prognostic model can aid physicians in formulating personalized treatment plans based on the specific tumor characteristics of a patient, thereby enhancing treatment efficacy and minimizing unnecessary side effects (31). Additionally, prognostic models assist in continuous monitoring and management of patient health, enabling timely detection of disease recurrence or progression (32). To better understand the prognostic factors of LUAD, we constructed a prognostic model based on macrophage-related genes. Initially, in the TCGA-LUAD dataset, we identified differentially expressed genes (DEGs) with significant expression differences between normal and tumor samples (logFC≥1, FDR<0.05) (**Figure 5A**). We then selected genes correlated with macrophage enrichment scores greater than 0.4 (p<0.05) and intersected them with the aforementioned DEGs. Using univariate Cox regression analysis, we identified genes significantly impacting LUAD prognosis in the TCGA dataset (**Figure 5B**). To optimize the model, we employed the LASSO Cox regression method and selected 14 key genes at the optimal λ value of 0.0323 (**Figure 5C**). The prognostic model was then constructed using multivariate Cox regression, with the formula: Risk Score = -0.39*CD101 - 0.099*CPA3 - 0.118*CD79A + 0.124*COL5A1 + 0.298*ERO1A + 0.082*GJB2. This formula estimates patient risk scores by weighting the expression levels of each gene. Patients were divided into high and low-risk groups based on the median score, and the prognostic ability and diagnostic accuracy of the model were validated in the TCGA database (**Figure 5D**). Kaplan-Meier survival curves indicated poorer prognosis for patients in the high-risk group. ROC curve analysis demonstrated that the model’s predictive AUC values for 1-year, 3-year, and 5-year survival rates were all above 0.65, confirming the model’s predictive efficacy. To ensure the robustness of the model, its prognostic and diagnostic capabilities were also validated in five independent GEO datasets (**Figures 5E–I**). Finally, we merged all the datasets after correcting for batch effects using the sva package. Survival analysis revealed that patients in the high-risk group had significantly worse prognosis compared to those in the low-risk group (**Figure 5J**). **Figure 5K** shows the effect of our batch effect correction. These external validations further confirmed the broad applicability and reliability of our model.

### 3.6 Identification of independent risk factors and construction of the nomogram

In our study, the risk score was integrated with clinical characteristics such as gender, age, and tumor stage for a comprehensive assessment. Through univariate and multivariate Cox regression analyses, key prognostic factors for LUAD were identified, including tumor stage and risk score (**Supplementary Figures 3A, B**). Based on these insights, we constructed a Nomogram that combines the risk score with clinical staging (**Supplementary Figure 3C**), aimed at precisely predicting the survival prognosis of LUAD patients. Calibration curves validated the high accuracy of the Nomogram, demonstrating its reliability in predicting LUAD survival rates (**Supplementary Figure 3D**). In terms of prognostic effectiveness, our Nomogram surpassed traditional clinical parameters in predictive accuracy (AUC), as further substantiated by time-dependent ROC curve analysis (**Supplementary Figure 3E**). Additionally, decision curve analysis (DCA) also confirmed the effectiveness of our Nomogram in identifying high-risk patients (**Supplementary Figure 3F**).

### 3.7 Enrichment analysis

The GSVA enrichment analysis revealed that the three most significantly enriched Hallmarker pathways in the high-risk group were GLYCOLYSIS, E2F_TARGETS, and G2M_CHECKPOINT(**Supplementary Figure 4A**). Notably, despite the presence of oxygen, tumor cells often prefer glycolysis to generate energy for rapid proliferation, a phenomenon known as the “Warburg effect” (33). This enhanced glycolysis is generally associated with metabolic reprogramming of cancer cells, a strategy adopted to meet their energy and biosynthetic demands for growth. Additionally, the E2F family of transcription factors (E2F_TARGETS) plays a crucial role in cell cycle regulation, especially in controlling the G1/S transition. The enrichment of E2F targets might indicate abnormalities in cell cycle control and accelerated cell proliferation, a hallmark of cancer (34). The G2/M checkpoint, a critical phase in the cell cycle, ensures cells complete DNA replication and damage repair before mitosis. The enrichment of the G2M_CHECKPOINT pathway suggests that in the high-risk group, cell cycle regulation and DNA damage response mechanisms might be activated or altered, potentially linked to rapid tumor growth and chemotherapy resistance (35). Further GSEA enrichment analysis indicated that pathways significantly enriched in the high-risk group included Chromosome Segregation and DNA Dependent DNA Replication (see **Supplementary Figure 4B**), while the low-risk group showed significant enrichment in pathways like B Cell Receptor Signaling Pathway and Immunoglobulin Complex (**Supplementary Figure 4C**). Subsequently, we assessed the relationship between the risk score, immune escape-related pathways, and tumor immune cycle (**Supplementary Figure 4D**). The analysis showed a positive correlation between the RiskScore and most immune escape-related pathways (except IFN-Gamma signature and APM signal), implying that patients in the high-risk group are more prone to immune escape. Notably, there was a significant negative correlation between the risk score and tumor immune cycle-related pathways. This suggests that as the risk score increases, the body’s immune system’s ability to recognize and eliminate tumor cells may decrease. Additionally, we evaluated the differences in immune cell infiltration and immune-related functions between high and low-risk groups (**Supplementary Figures 4E, F**). The results indicated that patients in the low-risk group had significantly better immune-related functions and immune cell infiltration than those in the high-risk group. Finally, the AUcell algorithm was used to assess the enrichment of genes involved in model construction across different cell types in the scRNA-seq dataset (**Supplementary Figure 4G**). The analysis showed that these genes were mainly enriched in macrophages and monocytes, further validating the accuracy of our analysis.

### 3.8 Immune microenvironment analysis

We downloaded immune infiltration data from seven algorithms (TIMER, CIBERSORT, CIBERSORT-ABS, QUANTISEQ, MCPCOUNTER, XCELL, and EPIC) from the Timer2.0 database. As shown in **Supplementary Figure 4A**, patients in the low-risk group exhibited higher levels of immune infiltration, suggesting that their tumors were more characteristic of “hot tumors” (36), known for better immunotherapy responsiveness. In contrast, patients in the high-risk group showed less immune cell infiltration, typically indicating lower sensitivity to immunotherapy due to reduced activation of the body’s immune response (37). Additionally, we employed the ‘estimate’ algorithm to assess the differences in immune infiltration levels between high and low-risk groups. This algorithm outputs four scores: stromal score representing stromal cell infiltration, immune score indicating immune cell infiltration, estimate score which is a combination of the two, and tumor purity that represents the purity of tumor cells. The results indicated that patients in the low-risk group had higher levels of stromal and immune cell infiltration, and the risk score was negatively correlated with stromal and immune cell infiltration levels (see **Supplementary Figures 4B–D**). Conversely, the high-risk group exhibited higher tumor purity, and the risk score was significantly positively correlated with tumor purity (**Supplementary Figure 4E**). The correlation analysis revealed a significant positive correlation between the risk score and M0 macrophage infiltration levels, while a significant negative correlation was observed with M2 macrophage infiltration (**Supplementary Figure 4F**).

### 3.9 Mutation analysis

**Figure 6:**
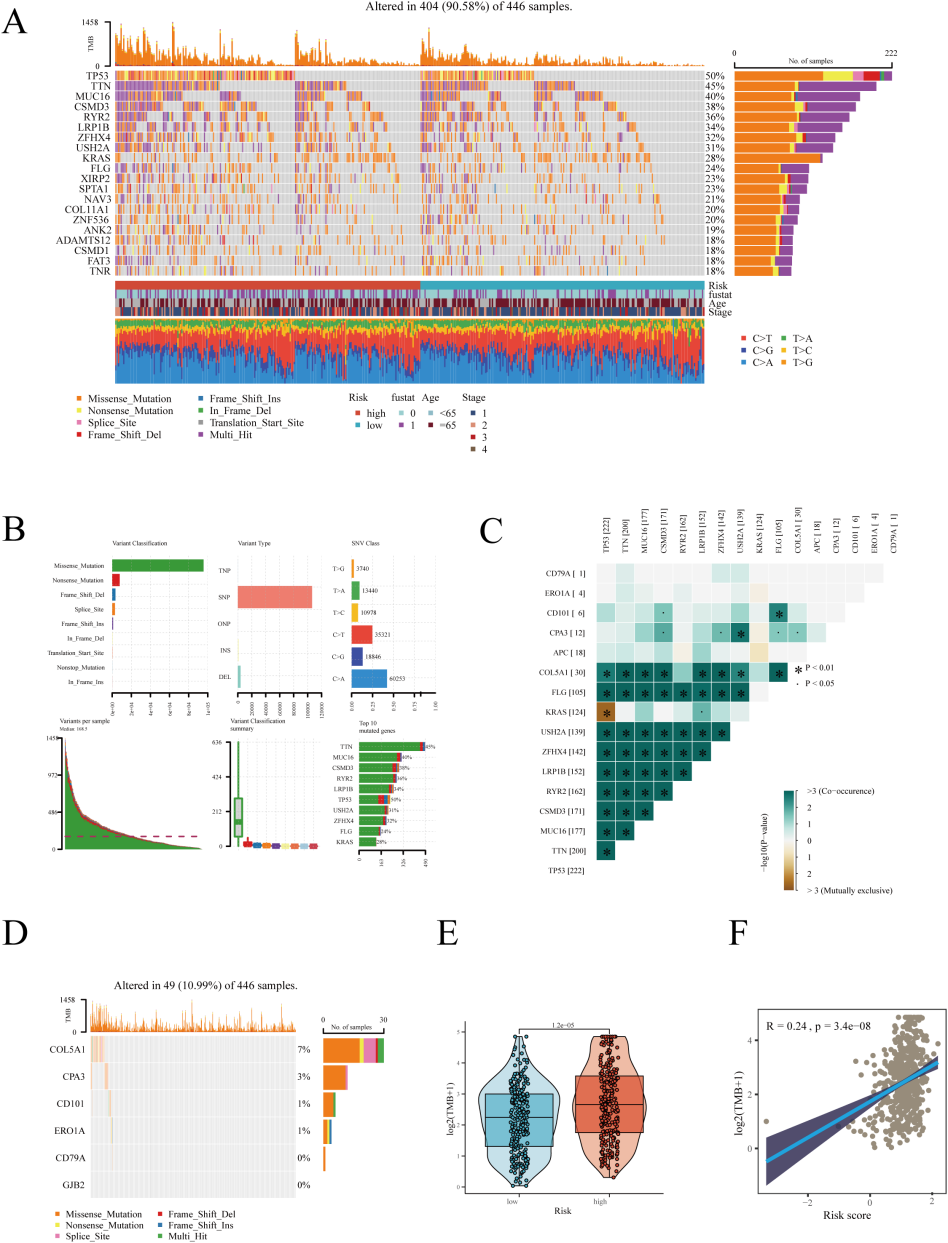
Gene mutation analysis in high and low-risk groups. **(A)** Heatmap displaying the mutation landscape in high and low-risk groups, highlighting the top 20 most frequently mutated genes. **(B)** Mutation landscape in TCGA-LUAD. **(C)** Co-mutation scenarios of hub genes. **(D)** Mutation information of hub genes. **(E)** Differences in TMB between high and low-risk groups. **(F)** Correlation between risk score and TMB. *P < 0.05.

In cancer research, analyzing the mutational landscape is crucial for uncovering tumor genesis and development mechanisms, tailoring treatment plans based on individual genetic characteristics, developing new targeted drugs, accurately predicting treatment responses and resistance, and enhancing early diagnosis and risk assessment accuracy. We first examined mutation scenarios in high and low-risk groups. After excluding samples with missing clinical information, 446 samples were analyzed, among which 404 exhibited gene mutations (**Figure 6A**). The waterfall plot displayed the top 20 genes with the highest mutation frequency, revealing a higher mutation frequency in the high-risk group. As shown in **Figure 6B**, in LUAD, the most common mutation type was SNP (single nucleotide polymorphism), predominantly featuring missense mutations. SNPs, a genetic mutation type, refer to variations at specific locations in the genome (38). These can occur in coding (genes) or non-coding regions and don’t necessarily lead to changes in protein sequences. Missense mutations, a specific type of SNP, occur in gene coding areas and result in altered amino acids, thus changing protein structure and function (39). Co-mutation analysis showed the co-mutation scenarios between hub genes and the top 10 most mutated genes (**Figure 6C**). **Figure 6D** detailed hub gene mutations, with COL5A1 having the highest mutation frequency: 30 mutations in 446 patients, primarily deletions. Subsequently, we found a higher TMB in the high-risk group, with a significant positive correlation between RiskScore and TMB (**Figures 6E, F**).

### 3.10 Immunotherapy efficacy prediction

**Figure 7:**
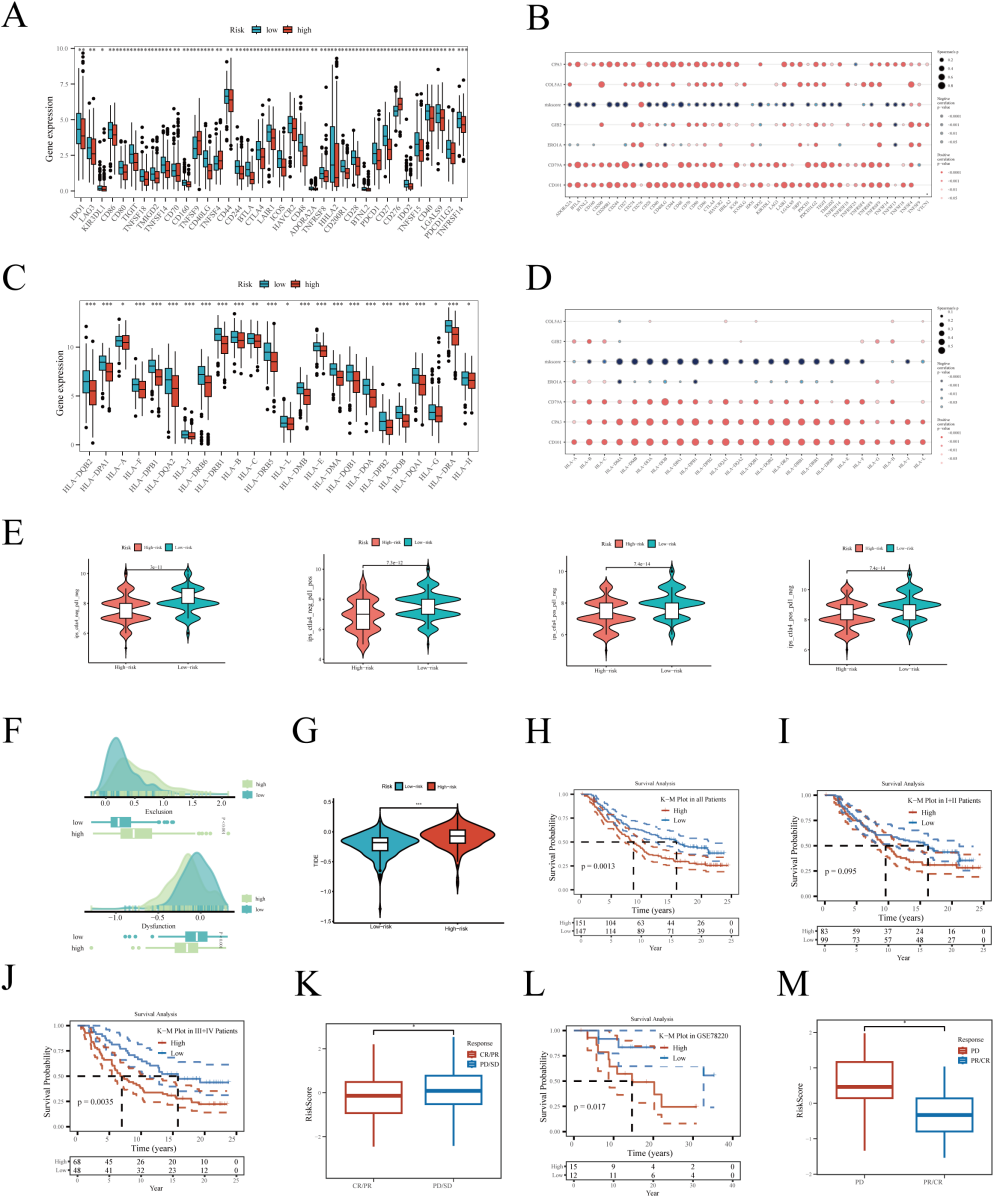
Relationship between risk score and immunotherapy. **(A)** Differential expression of immune checkpoint-related genes between high and low-risk groups. **(B)** Correlation of hub genes and risk score with checkpoint-related genes. **(C)** Differential expression of human major histocompatibility complex genes between high and low-risk groups. **(D)** Correlation of hub genes and risk score with human major histocompatibility complex genes. Differences in **(E)** IPS and **(F, G)** TIDE scores between high and low-risk groups. Kaplan-Meier survival analysis in the IMvigor210 cohort for **(H)** all, **(I)** stages I+II, and **(J)** stages III+IV patients. **(K)** Risk score differences between different immunotherapy outcomes. Survival analysis in the GSE78220 immunotherapy cohort **(L)** between high and low-risk groups. **(M)** Risk score differences between different immunotherapy efficacies. *P < 0.05; **P < 0.01; ***P < 0.001.

Immunotherapy has become a vital part of lung cancer treatment, significantly improving survival rates and quality of life, especially for advanced non-small cell lung cancer patients. We first evaluated the expression of immune checkpoint-related genes between high and low-risk groups, with almost all genes showing higher expression in the low-risk group (**Figure 7A**). Correlation analysis revealed that hub genes, especially *CD79A* and *CD101*, were significantly positively correlated with immune checkpoint-related genes (**Figure 7B**), while risk scores mainly showed a negative correlation. As **Figures 7C, D** depict, *HLA*-related genes were also significantly more expressed in the low-risk group, with risk scores showing a negative correlation with these genes. We then evaluated the IPS scores between high and low-risk groups, with higher scores indicating a higher likelihood of benefiting from immunotherapy. Results showed that IPS scores were significantly higher in the low-risk group, irrespective of *CTLA4* and *PDL1* expression levels (**Figure 7E**). The TIDE algorithm assessment of immune escape likelihood found that the high-risk group was more prone to immune dysfunction and exclusion (**Figures 7F, G**). In the IMvigor210 cohort, the prognosis of the high-risk group was significantly worse than that of the low-risk group (**Figure 7H**). No survival differences were observed between high and low-risk groups in stages I+II (**Figure 7I**), but in stages III+IV, the prognosis was worse for the high-risk group (**Figure 7J**), suggesting our prognostic model is more sensitive in predicting late-stage patient outcomes. Interestingly, the risk scores of patients in the partial response (PR) + complete response (CR) group were significantly lower than those in the progressive disease (PD) + stable disease (SD) group (**Figure 7K**). To further validate the robustness of the model, it was also tested in the additional immunotherapy cohort GSE78220, again finding poorer outcomes for the high-risk group (**Figure 7L**), with the PD group having significantly higher risk scores compared to the PR+CR group (**Figure 7M**).

### 3.11 Verification experiments for bioinformatics analysis

**Figure 8:**
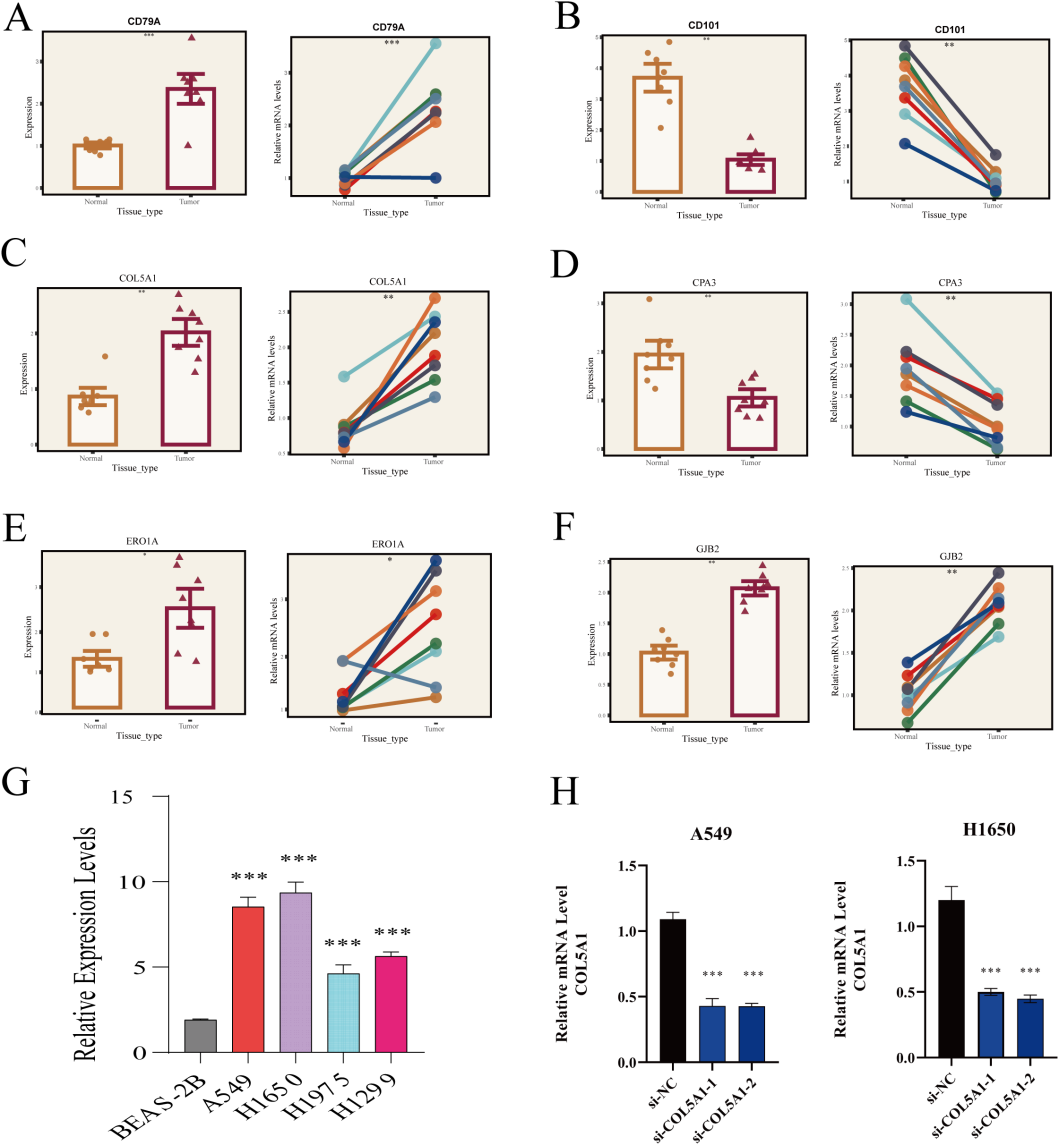
RT-PCR validation of HUB gene expression. **(A)** *CD79A*, **(B)** *CD101*, **(C)** *COL5A1*, **(D)** *CPA3*, **(E)** *ERO1A*, **(F)** *GJB2* expression in normal lung tissues and LUAD. **(G)** *COL5A1* expression in different cell lines. **(H)** Relative expression of *COL5A1* two days post-transfection. *P < 0.05; **P < 0.01; ***P < 0.001.

Acknowledging the potential for errors in our bioinformatics analysis, we conducted a series of validation experiments to ensure the accuracy of our results. Initially, we collected surgical resection samples of tumor and adjacent tissues from 8 LUAD patients at Tianjin Chest Hospital. Through these samples, we validated the differential expression of six key genes involved in model construction. Our findings indicated that *CD79A, COL5A1, ERO1A*, and *GJB2* were significantly overexpressed in tumor tissues compared to normal tissues, while *CD101* and *CPA3* showed more active expression in normal lung tissues (**Figures 8A–F**), aligning with our prior analysis.

Subsequently, we delved deeper into *COL5A1* research. RT-PCR analysis revealed significantly higher expression of *COL5A1* in four LUAD cell lines (A549, H1650, H1975, H1299) compared to the normal lung epithelial cell line (BEAS-2B) (**Figure 8G**). Next, we chose to knock down the expression of COL5A1 in the A549 and H1650 cell lines, which had the highest expression levels, using si-RNA technology. Two days after successful transfection, RT-PCR was used to verify the transfection efficiency, and the results showed that the gene was significantly downregulated at the RNA level (**Figure 8H**).

**Figure 9:**
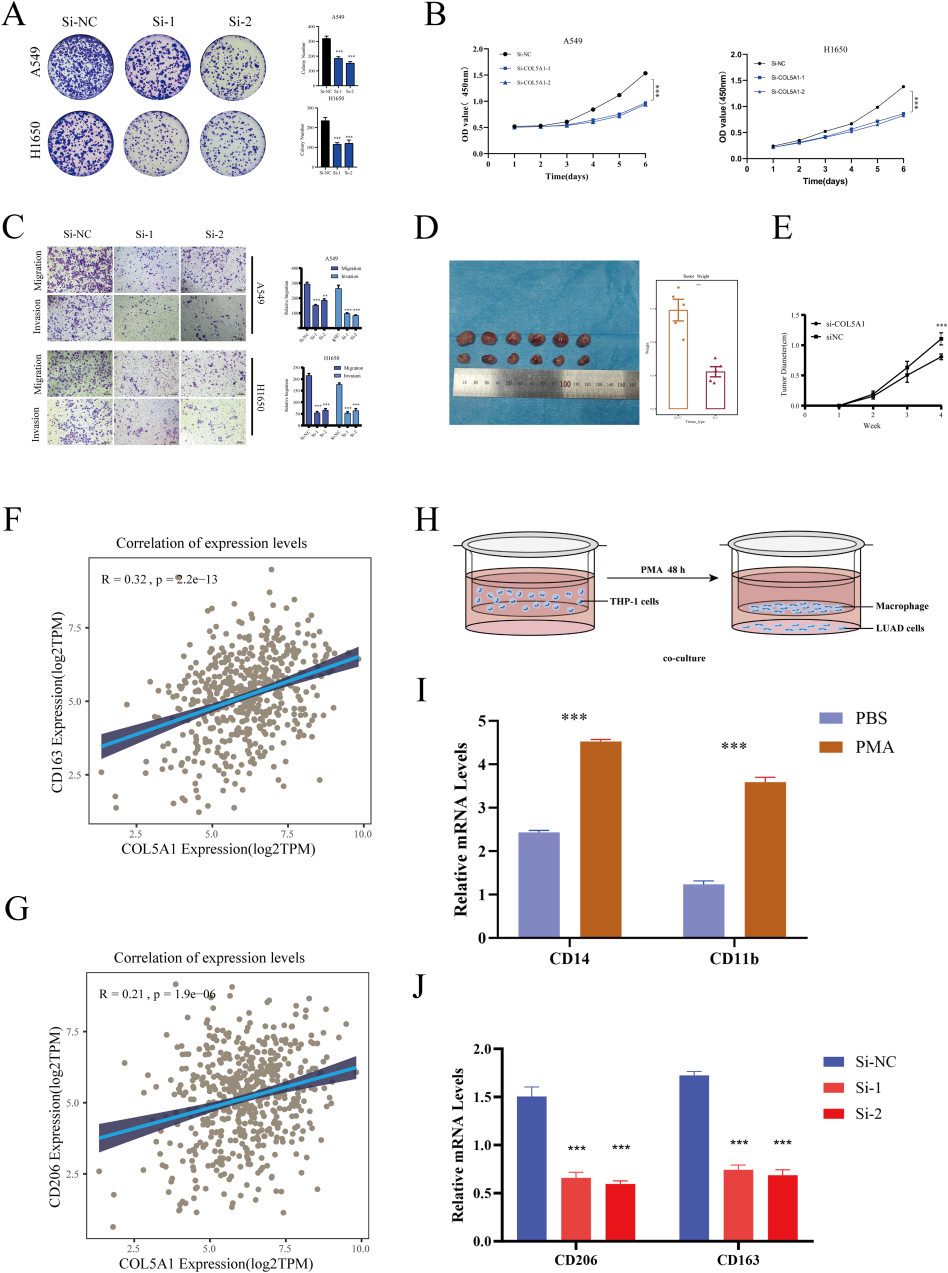
Cellular experiments. **(A)** Colony formation assay assessing the effect of *COL5A1* on cell proliferation. **(B)** CCK8 assay showing the impact of *COL5A1* knockdown on the proliferative capabilities of A549 and H1650 cell lines. **(C)** Transwell assay verifying the impact of *COL5A1* on cell migration and invasion. **(D)** Nude mice experiments simulate the effect of *COL5A1* on LUAD progression *in vivo*. **(E)** The tumor growth curves of mice in the COL5A1 knockdown group and the control group. Correlation of *COL5A1* with **(F)** *CD163*, **(G)** *CD206*. **(H)** Co-culture workflow diagram. **(I)** RT-PCR detection of macrophage induction effect. **(J)** RT-PCR validation of differential expression of anti-inflammatory macrophage (M2) markers after co-culture. *P < 0.05; **P < 0.01; ***P < 0.001.

Simultaneously, we conducted a series of cellular experiments to validate the role of COL5A1 in the progression of LUAD. Initially, colony formation assays showed that knocking down COL5A1 significantly reduced the clonogenic ability of LUAD cells (**Figure 9A**). CCK-8 assays demonstrated that knocking down COL5A1 inhibited the proliferative capacity of LUAD cells (**Figure 9B**). Transwell migration and invasion assays indicated that knocking down COL5A1 suppressed the migration and invasion abilities of LUAD cells (**Figure 9C**).

Given the focus of this study on macrophages, we further validated the relationship between COL5A1 and macrophages. Initially, we conducted a correlation analysis between COL5A1 and anti-inflammatory macrophage marker genes (CD163, CD206) in the TCGA-LUAD database (**Figures 9D, E**), which showed significant correlations. Then, we utilized a co-culture system to further validate the impact of COL5A1 on macrophage polarization, with **Figure 9F** displaying our workflow diagram. THP-1 cells were induced with 200 nM PMA for 48 hours, followed by RT-PCR to assess the expression of macrophage markers. The results showed that CD14 and CD11b were significantly upregulated after PMA induction, confirming the success of the induction (**Figure 9G**). Finally, macrophages were co-cultured with both normal and COL5A1-knockdown cell lines. The results demonstrated that knocking down COL5A1 significantly decreased the expression of CD206 and CD163, indicating that COL5A1 may promote the polarization of anti-inflammatory macrophages (**Figures 9F–J**).

## 4 Discussion

TME is an exceedingly complex ecosystem, comprising non-tumorous cells and their produced matrix environment that surround and support tumor growth. The TME encompasses various cell types, such as immune cells, stromal cells, endothelial cells, along with abundant extracellular matrix components, and is rich in cytokines, enzymes, and growth factors (7). A deep understanding of the TME is crucial for overcoming the challenges posed by cancer. ScRNA-seq, with its high-resolution capabilities, offers us a unique perspective to observe and analyze cellular differences and their collective compositions at the cellular level, which is particularly important for understanding the complex TME (12). Through scRNA-seq, we can intricately analyze various cellular subpopulations within the TME, gaining deeper insights into their specific roles in tumor progression. This, in turn, facilitates the development of more personalized and precise strategies for cancer treatment.

Macrophages are a crucial component of the Tumor TME and play a dual role in the onset and progression of cancer. This study initially categorized scRNA-seq data into eight cell clusters based on classic marker genes. Enrichment analysis revealed that macrophages are primarily involved in functions such as immune regulation, phagocytosis, and antigen presentation. We observed a declining trend in the number of macrophages in tumor tissues compared to adjacent non-tumor tissues, which might be attributed to the hypoxic environment in the TME that is unfavorable for the growth and aggregation of macrophages (40), or due to physical barriers formed by tumor cells and the secretion of specific immunoregulatory factors in the TME, such as TGF-β and IL-10, that inhibit the activation and recruitment of macrophages. Analysis of intercellular communication identified SPP1 and MIF as two key factors in potential carcinogenic mechanisms. The role of the SPP1 signaling pathway is crucial in cancer, involving cell adhesion, mobility, immune response, and inflammation (41, 42). SPP1 plays a significant role in tumor progression, invasion, metastatic ability, and poor prognosis. It attracts macrophages to the TME and induces their polarization towards tumor-promoting M2-type, thereby facilitating tumor progression and metastasis (43). The MIF signaling pathway also plays a key role in the TME, promoting angiogenesis, altering immune cell infiltration and function, and regulating inflammatory responses, thus driving tumor progression and potentially helping the tumor to evade the immune system by forming an immunosuppressive environment (44). Moreover, MIF can synergize with inflammatory pathways such as SPP1 and TNF-α, regulating the TME by interfering with macrophage polarization (45).

Based on current literature, we categorized macrophages into four subtypes: Alveolar-Mφ, Interstitial Mφ Perivascular, Mφ Anti-inflammatory, and Mφ Pro-inflammatory. Alveolar-Mφ, primarily located in the alveoli, serve as a crucial defense line of the respiratory system (28). Their anti-inflammatory properties are essential for maintaining lung stability and balance. Interstitial Mφ and Perivascular Mφ, positioned in tissue interstices and around blood vessels, play key roles in sustaining tissue equilibrium and responding to inflammation. However, in the cancer milieu, these cells may transform into an anti-inflammatory phenotype that promotes tumor progression (46). Mφ Pro-inflammatory, with pro-inflammatory characteristics, can resist pathogens and carcinogenic cells, demonstrating significant anti-cancer potential (47). Conversely, Mφ Anti-inflammatory, by inhibiting immune responses and facilitating tissue repair, may also contribute to tumor growth and metastasis (13). Pseudotime analysis was employed to determine the differentiation path of macrophages, starting from Alveolar-Mφ and transitioning to Interstitial Mφ Perivascular, eventually evolving into either Mφ Pro-inflammatory or Mφ Anti-inflammatory. Analysis from the TCGA database revealed that a high enrichment score of Mφ Anti-inflammatory is associated with a poorer prognosis in LUAD patients, while the other three macrophage subtypes positively impact prognosis.

Employing Lasso regression combined with multivariate COX regression analysis, we successfully developed a precise prognostic model for LUAD. Patients were categorized into high and low-risk groups based on the median risk score. Analysis in both training and validation sets indicated poorer survival outcomes for patients in the high-risk group. Moreover, the model demonstrated significant diagnostic efficacy in predicting the 1-, 3-, and 5-year survival rates of patients. Enrichment analysis of high and low-risk groups revealed that the metabolic reprogramming, aberrant cell cycle regulation, and activation of DNA damage response mechanisms exhibited by tumor cells in the high-risk group could be linked to rapid tumor growth and treatment resistance. In contrast, patients in the low-risk group exhibited stronger immune activity.

The tumor immune cycle, encompassing the entire process from the generation of tumor antigens to the immune cell-mediated clearance of tumor cells, involves complex interactions between tumor cells and the host immune system (48). Key stages of the tumor immune cycle include the release and presentation of tumor antigens, induction of immunogenic signals, activation and proliferation of immune cells, migration and infiltration of immune cells into the TME, recognition of tumor cells, and clearance of tumor cells mediated by effector immune cells (49). Each stage of the tumor immune cycle is crucial for immune-mediated tumor clearance, and dysfunction at any stage can lead to immune response failure and tumor immune escape. Our enrichment analysis showed a significant negative correlation between the risk score and most steps of the tumor immune cycle, suggesting that patients with higher risk are more prone to immune escape. Additionally, our analysis revealed a significant association between tumor immune infiltration characteristics and patient risk scores. Patients in the low-risk group displayed higher levels of immune infiltration, tending to form “hot tumors” that are more sensitive to immunotherapy. This underscores the importance of immune infiltration in patient prognosis and treatment response. Finally, we validated these hypotheses in the immunotherapy cohorts IMvigor210 and GSE78220, where results indicated that the low-risk group benefited more from immunotherapy, and patients with better treatment efficacy had lower risk scores.

A series of experiments were conducted to confirm the validity and reliability of our analysis. Initially, we verified the expression of Hub genes in LUAD tumor and adjacent non-tumor tissue samples, aligning our experimental results with the bioinformatics analysis. Our research then shifted focus to *COL5A1*, a gene located on the q arm of chromosome 9 encoding the alpha-1 chain of type V collagen (50), crucial for collagen fiber assembly and the structural integrity of the ECM (51). Previous studies have indicated *COL5A1*’s role in gastric cancer progression through acting as a ceRNA for miR-137-3p to promote FSTL1 expression (52) and its association with ovarian cancer progression, taxol resistance, and immune cell infiltration in the tumor environment (53). Earlier bioinformatics research suggested that reducing *COL5A1* expression could inhibit glioma cell proliferation and migration and increase sensitivity to the chemotherapeutic drug temozolomide (54). However, *COL5A1*’s role in LUAD remained unclear. To address this, we conducted experiments on A549 and H1650 cell lines, demonstrating that *COL5A1* is significantly upregulated in LUAD. Silencing *COL5A1* effectively inhibited tumor cell growth, invasion, and migration while enhancing apoptosis, providing experimental evidence for its potential role in LUAD treatment.

Overall, our study reveals the heterogeneity of macrophages in LUAD and utilizes their marker genes to construct a prognostic model, offering new insights into LUAD diagnosis and treatment. However, our research has limitations, primarily relying on bioinformatics analysis based on public databases, lacking sufficient experimental validation. In the future, we plan to conduct a series of basic and clinical trials to validate the potential carcinogenic mechanisms of macrophages in LUAD, hoping to offer new strategies and hope for treatment.

## Data Availability

The original contributions presented in the study are included in the article/**Supplementary Material**. Further inquiries can be directed to the corresponding authors.
