# Supplementary material for: Macrophage heterogeneity and oncogenic mechanisms in lung adenocarcinoma: insights from scRNA-seq analysis and predictive modeling
Source: Front Immunol. 2025 Jan 9;15:1491872. doi: 10.3389/fimmu.2024.1491872 (PMC11754191; doi:10.3389/fimmu.2024.1491872)
Supplement: Supplementary file 1 [file DataSheet1.docx]

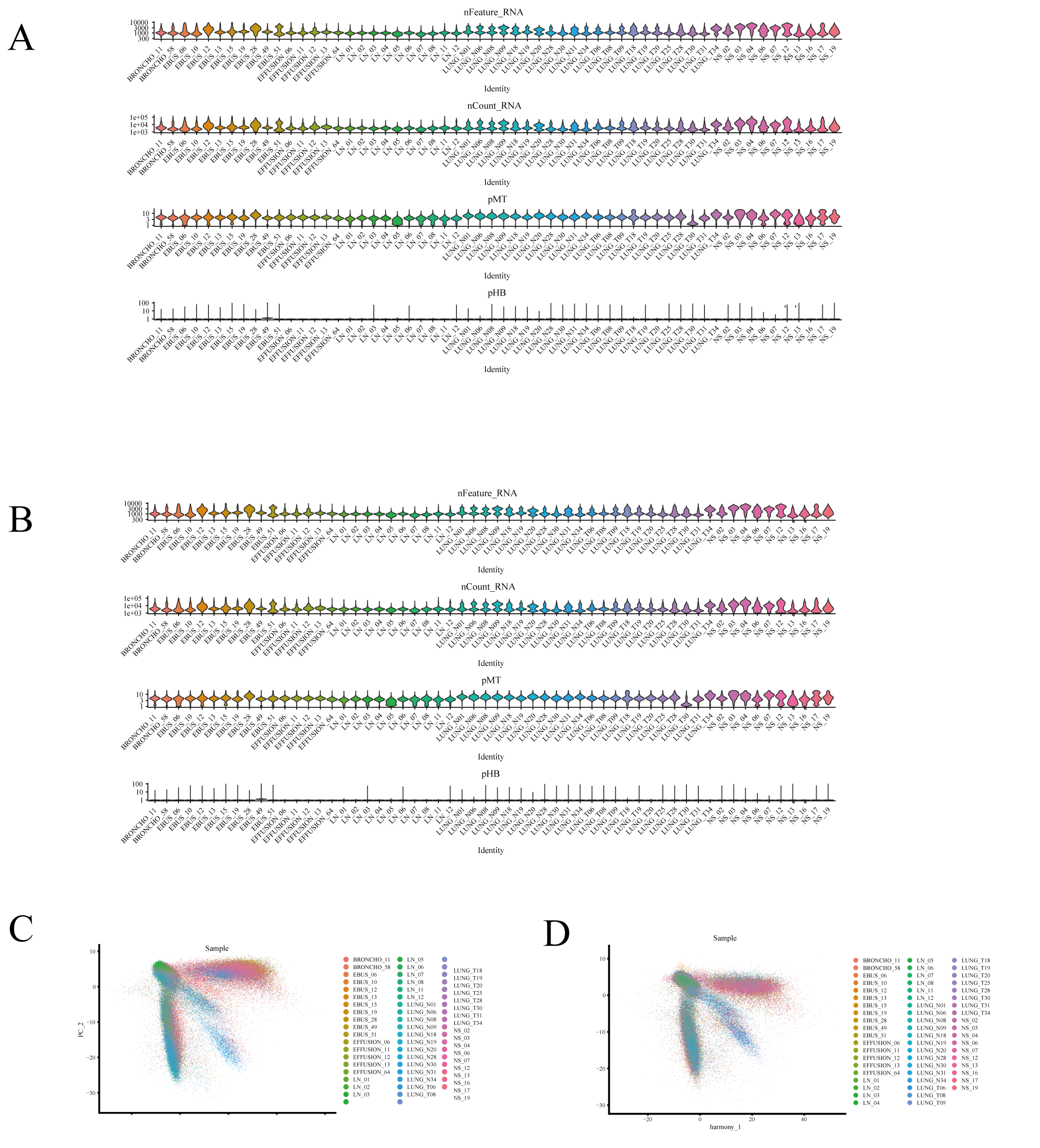


**Supplement Figure1:** Quality control information for single-cell data. The distribution of nFeature_RNA, nCount_RNA, mitochondrial gene percentage, and red blood cell gene percentage in single-cell data before (A) and after (B) quality control. The distribution of samples before (C) and after (D) batch effect removal using the Harmony R package.


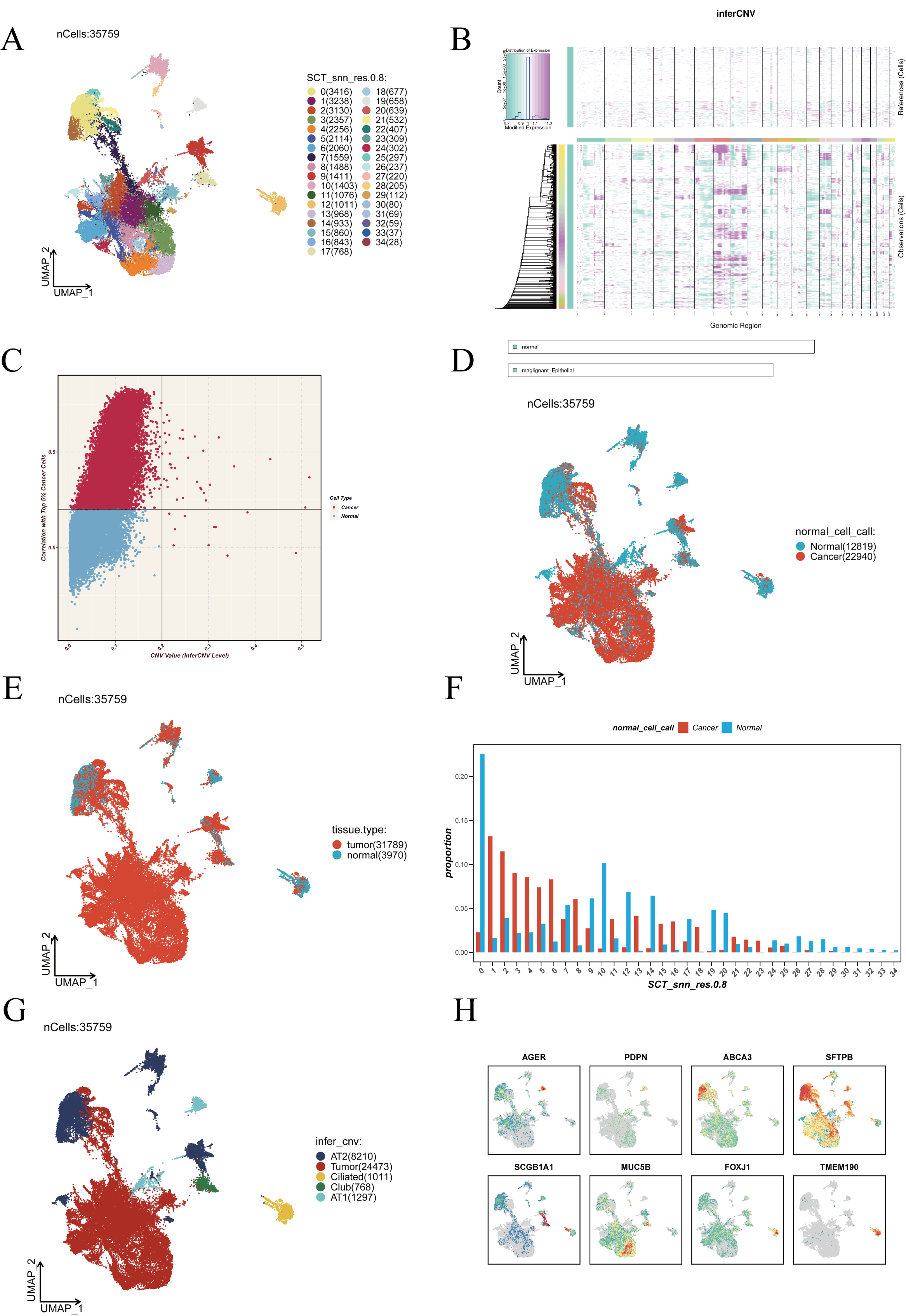


**Supplement Figure2:** Based on the infercnv algorithm, the benign or malignant status of epithelial cells was inferred. (A) Epithelial cells were clustered into 35 distinct cell groups. (B) The chromosomal amplifications and deletions in epithelial cells from normal tissues and tumor tissues were compared using the Infercnv algorithm. (C) A scatter plot shows the correlation between the CNA values of cells and the top 5% of cancer cells, with red representing malignant cells and blue representing normal cells. (D) UMAP plot shows the inferred benign or malignant status of cells based on infercnv. (E) UMAP plot shows the tissue origin of all epithelial cells. (F) A bar chart displays the proportion of benign and malignant cells in each cell cluster (inferred by infercnv). (G) Reannotation of epithelial cells. (H) Expression levels of epithelial cell marker genes


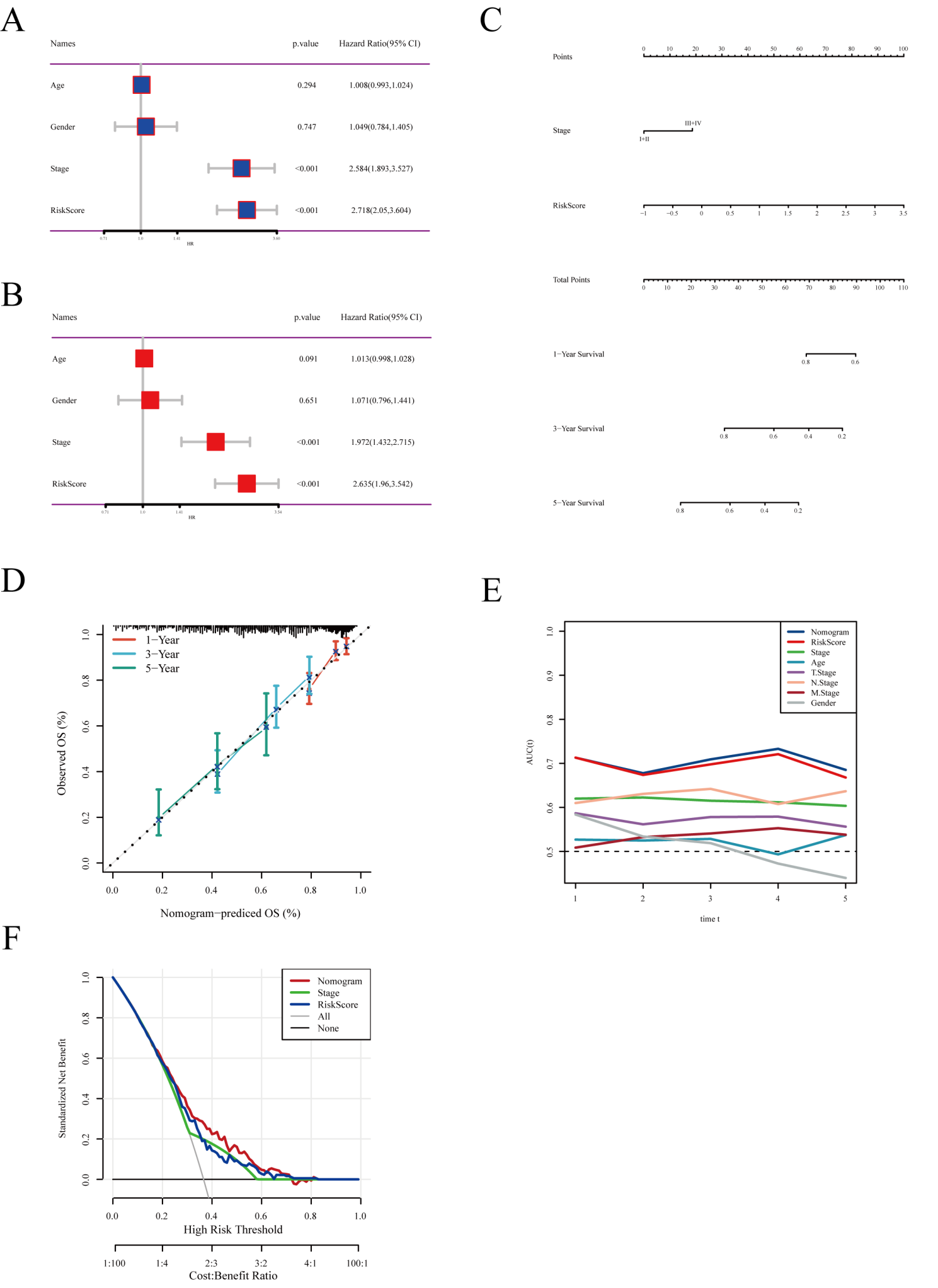
**Supplement Figure3:** Integration of risk score and clinical features to establish a new nomogram. (A) Univariate COX analysis results based on risk score and clinical features. (B) Multivariate COX analysis results based on risk score and clinical features. (C) The constructed nomogram. (D) Calibration curves for 1-, 3-, and 5-year predictions by the nomogram. (E) Time-ROC analysis comparing the predictive capability of the nomogram with other clinical features. (F) Decision curve analysis for the nomogram.


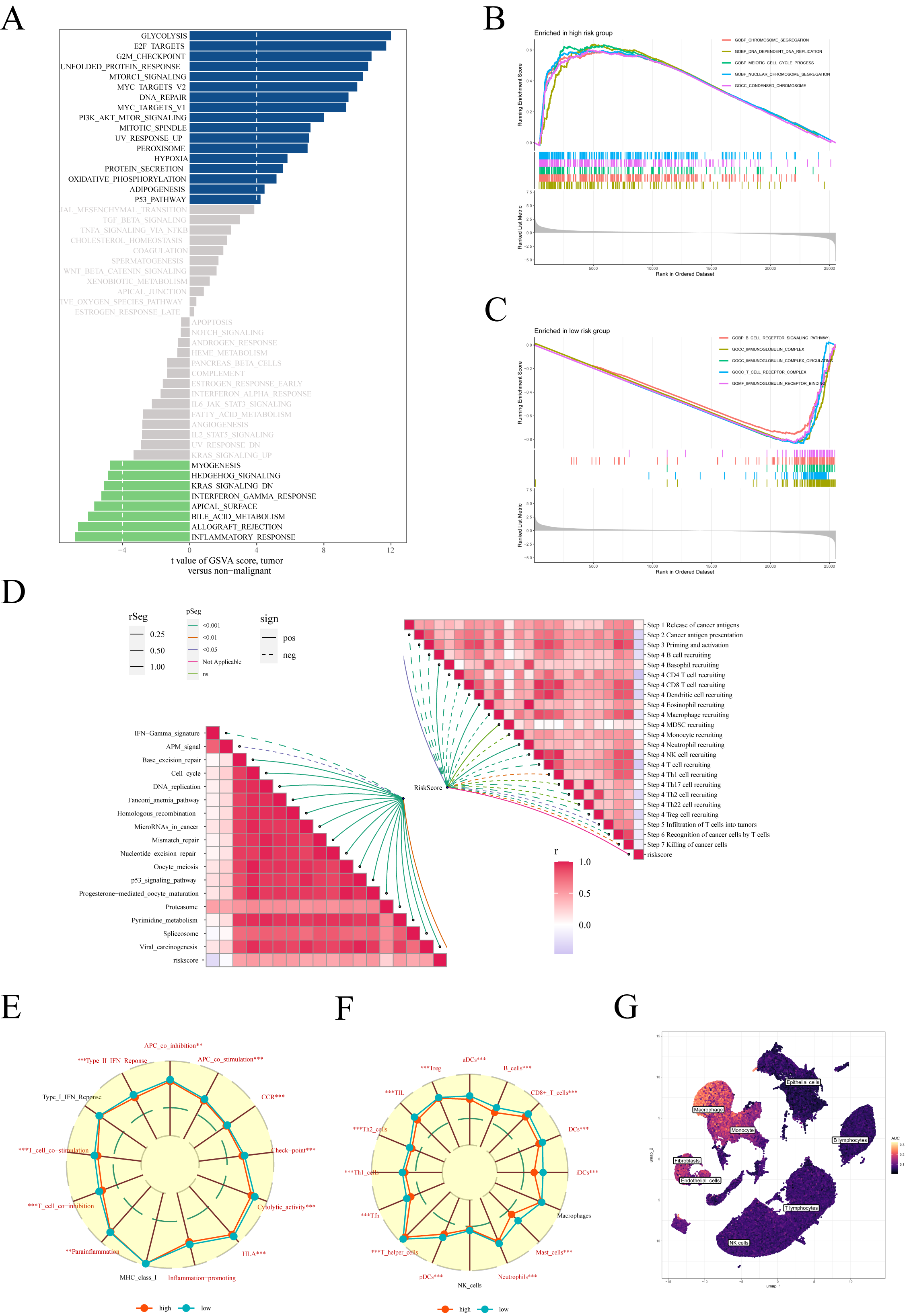
**Supplement Figure4:** Enrichment Analysis. (A) Gene Set Variation Analysis showing Hallmarker pathways significantly enriched in the high-risk group compared to the low-risk group. (B)Gene Set Enrichment Analysis revealing the top 5 GO pathways most related to the high-risk group, (C) and the low-risk group. (D) Heatmap illustrating the relationship between risk score, tumor immune-related scores, and pathways related to immunotherapy efficacy. Radar charts showing differences in (E) immune cells and (F) immune-related functions between high and low-risk groups, based on the single-sample Gene Set Enrichment Analysis . (G) AUcell algorithm assesses the enrichment of gene sets with a correlation greater than 0.4 with macrophage enrichment scores in scRNA-seq data from the TCGA database


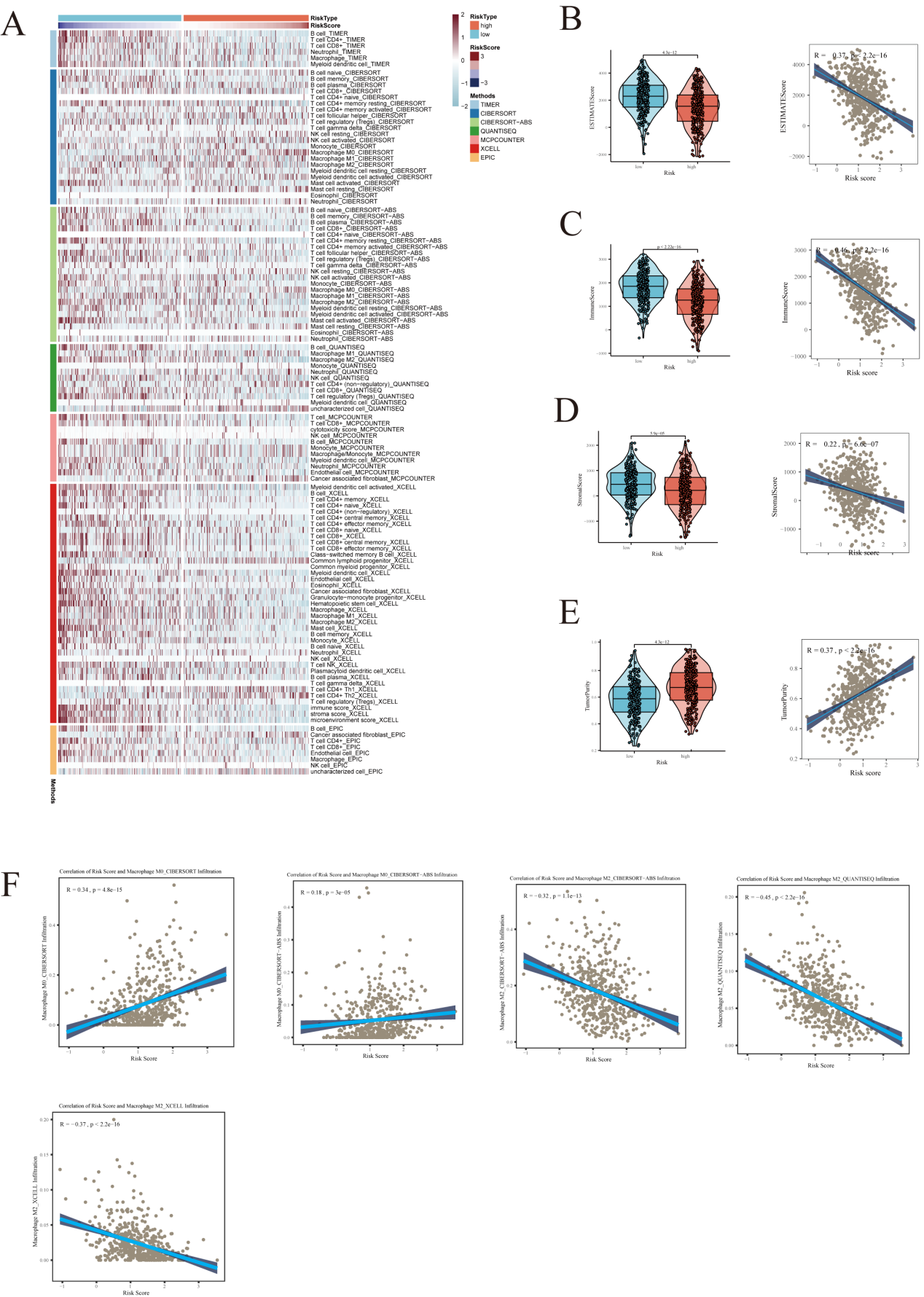


**Supplement Figure5:** Immune Microenvironment Assessment. (A) Evaluation of immune cell infiltration differences between high and low-risk groups based on seven databases. Assessment of (B) ESTIMATE Score, (C) Immune Score, (D) Stromal Score, (E) Tumor Purity scores derived from the Estimate algorithm, their differences between high and low-risk groups, and their correlation with risk scores.(F) Correlation between risk score and macrophage infiltration level.

| Dataet ID | Sample Size | Platform Information | Data Type | Article DOI |
| --- | --- | --- | --- | --- |
| GSE31210 | 246 | GPL570 | Expression profiling by array | 10.1158/0008-5472.CAN-11-1403 |
| GSE37745 | 196 | GPL570 | Expression profiling by array | 10.1158/1078-0432.CCR-12-1139 |
| GSE50081 | 181 | GPL570 | Expression profiling by array | 10.1097/JTO.0000000000000042 |
| GSE68465 | 462 | GPL96 | Expression profiling by array | 10.1038/nm.1790 |
| GSE3141 | 111 | GPL570 | Expression profiling by array | 10.1038/nature04296 |

Supplementary Table 1: Detailed Information of the Five Independent GEO Validation Cohorts

| **Oligonucleotides** | **Nucleotide sequence (5'-3')** |
| --- | --- |
| **siRNA** | (Antisense Strand) |
| siRNA-NC | GCUUCGCGCCGUAGUCUUA |
| si COL5A1-1 | CCUUCCGUGUAAUAUUCAUCUTT |
| si COL5A1-2 | AGUAGUUCUCGUCAAGGUUCCTT |
| **Primer** | **Nucleotide sequence (5'-3')** |
| **GAPDH** | GGCCTCCAAGGAGTAAGACC (Forward) |
|  | AGGGGAGATTCAGTGTGGTG (Reverse) |
| **COL5A1** | TGACAAGAAGTCCGAAGGGG (Forward) |
|  | CGTCCACATAGGAGAGCAGTT(Reverse) |
| **CD79A** | GGCAACGAGTCATACCAGCA (Forward) |
|  | CGTTCTGCCATCGTTTCCTG(Reverse) |
| **CD101** | GGATCCAGCCAGCAGTGAAA (Forward) |
|  | AATGCCTTGAAGCTGTGGGT(Reverse) |
| **CPA3** | GGTCATGGACAAAGAACCGC (Forward) |
|  | TGCACATGGGTCATTGGTGT(Reverse) |
| **GJB2** | CTCCCGACGCAGAGCAAA (Forward) |
|  | GGGTGTTGCAGACAAAGTCG (Reverse) |
| **ERO1A** | CGACTTGGAGCAGTGGATGAA (Forward) |
|  | CCACCAGCAGATCCAATCATCA(Reverse) |

Supplementary Table 2: Sequence of COL5A1 siRNA and primers for the model gene.

| Gene | avg_log2FC | p_val_adj | cluster |
| --- | --- | --- | --- |
| FABP4 | 2.80693317057177 | 0 | Alveolar-Mφ |
| MCEMP1 | 1.97644855842693 | 0 | Alveolar-Mφ |
| CD52 | 1.85532821941173 | 0 | Alveolar-Mφ |
| ALDH2 | 1.64583405655322 | 0 | Alveolar-Mφ |
| CES1 | 1.64427952410018 | 0 | Alveolar-Mφ |
| PDLIM1 | 1.54312155138723 | 0 | Alveolar-Mφ |
| SERPINA1 | 1.50123116779844 | 0 | Alveolar-Mφ |
| MARCO | 1.48110458463866 | 0 | Alveolar-Mφ |
| GCHFR | 1.43666769585592 | 0 | Alveolar-Mφ |
| CRIP1 | 1.42570341440144 | 0 | Alveolar-Mφ |
| FBP1 | 1.41278289594338 | 0 | Alveolar-Mφ |
| INHBA | 1.33801994629966 | 0 | Alveolar-Mφ |
| STXBP2 | 1.31810380342636 | 0 | Alveolar-Mφ |
| SERPING1 | 1.2603660324728 | 0 | Alveolar-Mφ |
| FOLR3 | 1.2508873773151 | 0 | Alveolar-Mφ |
| HLA-DRB5 | 1.1330771908172 | 0 | Alveolar-Mφ |
| RETN | 1.12204097721427 | 0 | Alveolar-Mφ |
| IGFBP2 | 1.11568351955468 | 0 | Alveolar-Mφ |
| LTA4H | 1.11509813873665 | 0 | Alveolar-Mφ |
| GLRX | 1.10465476700183 | 0 | Alveolar-Mφ |
| TREM1 | 1.09414974974635 | 0 | Alveolar-Mφ |
| S100A4 | 1.06949226759582 | 0 | Alveolar-Mφ |
| CD9 | 1.0684112133317 | 0 | Alveolar-Mφ |
| RBP4 | 1.0674631717117 | 0 | Alveolar-Mφ |
| TSPO | 1.06278969278498 | 0 | Alveolar-Mφ |
| UBB | 1.01118564229887 | 0 | Alveolar-Mφ |
| RP11-598F7.3 | 1.00952155713499 | 0 | Alveolar-Mφ |
| S100A13 | 0.985488942201528 | 0 | Alveolar-Mφ |
| AQP3 | 0.982855895847417 | 0 | Alveolar-Mφ |
| RND3 | 0.976765576199206 | 0 | Alveolar-Mφ |
| LGALS3 | 0.95090329014921 | 0 | Alveolar-Mφ |
| LPL | 0.949512032060137 | 0 | Alveolar-Mφ |
| PLA2G16 | 0.933480141675097 | 0 | Alveolar-Mφ |
| ALOX5 | 0.931083906321397 | 0 | Alveolar-Mφ |
| PHLDA3 | 0.924030160821657 | 0 | Alveolar-Mφ |
| ACP5 | 0.897545361549692 | 0 | Alveolar-Mφ |
| PLBD1 | 0.88979083615557 | 0 | Alveolar-Mφ |
| GPD1 | 0.889238225236508 | 0 | Alveolar-Mφ |
| GLIPR2 | 0.864420743377747 | 0 | Alveolar-Mφ |
| TAGLN2 | 0.856701639187188 | 0 | Alveolar-Mφ |
| PNPLA6 | 0.850310033284746 | 0 | Alveolar-Mφ |
| CYP27A1 | 0.845378574946682 | 0 | Alveolar-Mφ |
| MSR1 | 0.840789161435363 | 0 | Alveolar-Mφ |
| SCD | 0.834856853207287 | 0 | Alveolar-Mφ |
| RHBDD2 | 0.823866305565495 | 0 | Alveolar-Mφ |
| S100A6 | 0.823047828018672 | 0 | Alveolar-Mφ |
| ACOT7 | 0.820366991443077 | 0 | Alveolar-Mφ |
| SPN | 0.807693477764869 | 0 | Alveolar-Mφ |
| HLA-DRA | 0.80619132127266 | 0 | Alveolar-Mφ |
| HLA-DRB1 | 0.803122786278229 | 0 | Alveolar-Mφ |
| HLA-DQB1 | 0.800366233986684 | 0 | Alveolar-Mφ |
| FN1 | 0.798939564 | 0 | Alveolar-Mφ |
| SVIL | 0.797022536954117 | 0 | Alveolar-Mφ |
| CCL18 | 0.793413835851229 | 0 | Alveolar-Mφ |
| TNNI2 | 0.792206372378776 | 0 | Alveolar-Mφ |
| PPARG | 0.77583372144212 | 0 | Alveolar-Mφ |
| RGCC | 0.771811604967808 | 0 | Alveolar-Mφ |
| PCOLCE2 | 0.768499966416274 | 0 | Alveolar-Mφ |
| CXCL16 | 0.764036887459923 | 0 | Alveolar-Mφ |
| SCGB1A1 | 0.755674565555726 | 0 | Alveolar-Mφ |
| TFRC | 0.751809838425002 | 0 | Alveolar-Mφ |
| HLA-DQA1 | 0.749746997611617 | 0 | Alveolar-Mφ |
| FHL1 | 0.740309186568997 | 0 | Alveolar-Mφ |
| TGM2 | 0.738634740729053 | 0 | Alveolar-Mφ |
| RMDN3 | 0.737692006336076 | 0 | Alveolar-Mφ |
| GS1-600G8.5 | 0.733482756319994 | 0 | Alveolar-Mφ |
| MRC1 | 0.731627998831546 | 0 | Alveolar-Mφ |
| SEPT11 | 0.731092278922732 | 0 | Alveolar-Mφ |
| MT-ND2 | 0.719603385104218 | 0 | Alveolar-Mφ |
| MGST3 | 0.713674831943131 | 0 | Alveolar-Mφ |
| JAML | 0.713394066504966 | 0 | Alveolar-Mφ |
| RP5-839B4.8 | 0.711520118346263 | 0 | Alveolar-Mφ |
| CSTA | 0.70585962740078 | 0 | Alveolar-Mφ |
| EVL | 0.697998438399012 | 0 | Alveolar-Mφ |
| GRN | 0.69585461278235 | 0 | Alveolar-Mφ |
| TCF7L2 | 0.688848326380216 | 0 | Alveolar-Mφ |
| DOK2 | 0.680027381935435 | 0 | Alveolar-Mφ |
| LDHB | 0.674959638532635 | 0 | Alveolar-Mφ |
| DECR1 | 0.673800710383506 | 0 | Alveolar-Mφ |
| TXNIP | 0.672560837575874 | 0 | Alveolar-Mφ |
| TKT | 0.671172033565672 | 0 | Alveolar-Mφ |
| MME | 0.655250986668718 | 0 | Alveolar-Mφ |
| C20orf27 | 0.652957774107704 | 0 | Alveolar-Mφ |
| ANXA1 | 0.64409239587318 | 0 | Alveolar-Mφ |
| PPIC | 0.634250679810656 | 0 | Alveolar-Mφ |
| LGALS3BP | 0.631981009989695 | 0 | Alveolar-Mφ |
| CARD16 | 0.63034720423732 | 0 | Alveolar-Mφ |
| ABCG1 | 0.624479977881053 | 0 | Alveolar-Mφ |
| CAMP | 0.615009600911616 | 0 | Alveolar-Mφ |
| ALOX5AP | 0.610104091631551 | 0 | Alveolar-Mφ |
| ABHD5 | 0.601221034373853 | 0 | Alveolar-Mφ |
| SNX10 | 0.598352303644921 | 0 | Alveolar-Mφ |
| C1QA | 0.596280715096763 | 0 | Alveolar-Mφ |
| CD37 | 0.590365732317599 | 0 | Alveolar-Mφ |
| ATP1B1 | 0.588502436981643 | 0 | Alveolar-Mφ |
| HDDC2 | 0.588011384879578 | 0 | Alveolar-Mφ |
| PEBP1 | 0.58305423832316 | 0 | Alveolar-Mφ |
| FTH1 | 0.581348892078339 | 0 | Alveolar-Mφ |
| AKR1C3 | 0.580026649274925 | 0 | Alveolar-Mφ |
| PTMS | 0.579830003857713 | 0 | Alveolar-Mφ |
| LST1 | 0.579718628779736 | 0 | Alveolar-Mφ |
| HCAR2 | 0.572953467539282 | 0 | Alveolar-Mφ |
| MTPN | 0.572754282723967 | 0 | Alveolar-Mφ |
| SPI1 | 0.571124512895378 | 0 | Alveolar-Mφ |
| RTN4 | 0.568717743996891 | 0 | Alveolar-Mφ |
| BHLHE41 | 0.567495533429067 | 0 | Alveolar-Mφ |
| OLR1 | 0.565517566876863 | 0 | Alveolar-Mφ |
| CD74 | 0.563475478531442 | 0 | Alveolar-Mφ |
| FAM89A | 0.560795717281966 | 0 | Alveolar-Mφ |
| C1orf162 | 0.560451173659517 | 0 | Alveolar-Mφ |
| NMB | 0.559756577203295 | 0 | Alveolar-Mφ |
| MS4A7 | 0.558333884177891 | 0 | Alveolar-Mφ |
| SFTPC | 0.558102132734983 | 0 | Alveolar-Mφ |
| RNF149 | 0.55503997251385 | 0 | Alveolar-Mφ |
| BSG | 0.554173630428712 | 0 | Alveolar-Mφ |
| FFAR4 | 0.553796115469408 | 0 | Alveolar-Mφ |
| HLA-DPA1 | 0.54662454758476 | 0 | Alveolar-Mφ |
| MT-CYB | 0.545280787605771 | 0 | Alveolar-Mφ |
| RAB11FIP1 | 0.543903412751428 | 0 | Alveolar-Mφ |
| LRPAP1 | 0.536740582748318 | 0 | Alveolar-Mφ |
| PECAM1 | 0.533993202009229 | 0 | Alveolar-Mφ |
| MT-CO1 | 0.533899384397599 | 0 | Alveolar-Mφ |
| TXN | 0.532084088311282 | 0 | Alveolar-Mφ |
| ANXA2 | 0.528728141182606 | 0 | Alveolar-Mφ |
| FDFT1 | 0.528135416752894 | 0 | Alveolar-Mφ |
| LYAR | 0.526763971500494 | 0 | Alveolar-Mφ |
| SEC11A | 0.526482156579098 | 0 | Alveolar-Mφ |
| VMO1 | 0.52464404454004 | 0 | Alveolar-Mφ |
| PTPMT1 | 0.524020039627211 | 0 | Alveolar-Mφ |
| VASP | 0.523822388549257 | 0 | Alveolar-Mφ |
| ETHE1 | 0.52268263123742 | 0 | Alveolar-Mφ |
| C1QB | 0.520221427461767 | 0 | Alveolar-Mφ |
| S100A10 | 0.518713878107212 | 0 | Alveolar-Mφ |
| COMT | 0.518609083278393 | 0 | Alveolar-Mφ |
| C9orf16 | 0.518451088083491 | 0 | Alveolar-Mφ |
| ADAMTSL4 | 0.518256805360929 | 0 | Alveolar-Mφ |
| TCEB2 | 0.514007523634765 | 0 | Alveolar-Mφ |
| CD151 | 0.511403622433391 | 0 | Alveolar-Mφ |
| AGRP | 0.511054700392872 | 0 | Alveolar-Mφ |
| HLA-C | 0.502210236263691 | 0 | Alveolar-Mφ |
| ACO1 | 0.501116501293471 | 0 | Alveolar-Mφ |
| GYPC | 0.498615992021895 | 0 | Alveolar-Mφ |
| OSCAR | 0.494904481633545 | 0 | Alveolar-Mφ |
| MT-CO3 | 0.494346828371238 | 0 | Alveolar-Mφ |
| HPGD | 0.492251222551967 | 0 | Alveolar-Mφ |
| S1PR4 | 0.49184223932662 | 0 | Alveolar-Mφ |
| AVPI1 | 0.486374296527503 | 0 | Alveolar-Mφ |
| LAMTOR1 | 0.485258432461309 | 0 | Alveolar-Mφ |
| ARL4A | 0.484734855376112 | 0 | Alveolar-Mφ |
| LYZ | 0.479609790944371 | 0 | Alveolar-Mφ |
| ADTRP | 0.477384156898251 | 0 | Alveolar-Mφ |
| TMSB4X | 0.473736174117874 | 0 | Alveolar-Mφ |
| VIM | 0.471218877512244 | 0 | Alveolar-Mφ |
| MYL12A | 0.467013605510194 | 0 | Alveolar-Mφ |
| FDX1 | 0.459838029664049 | 0 | Alveolar-Mφ |
| LRRFIP1 | 0.459652972112654 | 0 | Alveolar-Mφ |
| EVI2B | 0.45597076740336 | 0 | Alveolar-Mφ |
| SNX2 | 0.453814153969251 | 0 | Alveolar-Mφ |
| MACC1 | 0.453614916790456 | 0 | Alveolar-Mφ |
| TRPV2 | 0.449616135721267 | 0 | Alveolar-Mφ |
| OASL | 0.448861086518542 | 0 | Alveolar-Mφ |
| MYL6 | 0.448859389206869 | 0 | Alveolar-Mφ |
| NCF2 | 0.445089548759721 | 0 | Alveolar-Mφ |
| FLNA | 0.445051092958904 | 0 | Alveolar-Mφ |
| PRSS21 | 0.442774160528509 | 0 | Alveolar-Mφ |
| PYCARD | 0.441902000148334 | 0 | Alveolar-Mφ |
| ADGRE5 | 0.441598676717132 | 0 | Alveolar-Mφ |
| NOP10 | 0.441275311349478 | 0 | Alveolar-Mφ |
| GPCPD1 | 0.438653324700762 | 0 | Alveolar-Mφ |
| CLEC12A | 0.438347558872424 | 0 | Alveolar-Mφ |
| LY6E | 0.437072166705226 | 0 | Alveolar-Mφ |
| AMIGO2 | 0.43492195385711 | 0 | Alveolar-Mφ |
| APIP | 0.427598977976356 | 0 | Alveolar-Mφ |
| CPE | 0.426255427358861 | 0 | Alveolar-Mφ |
| TBC1D10C | 0.426118004127769 | 0 | Alveolar-Mφ |
| EEF1A1 | 0.424002037093854 | 0 | Alveolar-Mφ |
| CXCL5 | 0.420425711712456 | 0 | Alveolar-Mφ |
| CCND3 | 0.4188051708717 | 0 | Alveolar-Mφ |
| DNAJA1 | 0.418380831370716 | 0 | Alveolar-Mφ |
| CLDN7 | 0.418264631460007 | 0 | Alveolar-Mφ |
| CITED2 | 0.415293800645866 | 0 | Alveolar-Mφ |
| S100A11 | 0.414916606714816 | 0 | Alveolar-Mφ |
| DNASE2B | 0.412502026422334 | 0 | Alveolar-Mφ |
| MT-CO2 | 0.411293272824464 | 0 | Alveolar-Mφ |
| CA2 | 0.4100745680391 | 0 | Alveolar-Mφ |
| SLC7A7 | 0.409645131876861 | 0 | Alveolar-Mφ |
| MYADM | 0.409192436552307 | 0 | Alveolar-Mφ |
| SMIM14 | 0.408908512313764 | 0 | Alveolar-Mφ |
| LINC01272 | 0.408437058134538 | 0 | Alveolar-Mφ |
| USP30-AS1 | 0.405470197516221 | 0 | Alveolar-Mφ |
| GMFG | 0.402536713908566 | 0 | Alveolar-Mφ |
| HBEGF | 0.402343473851085 | 0 | Alveolar-Mφ |
| RAC2 | 0.400361689847725 | 0 | Alveolar-Mφ |
| MT-ND5 | 0.399339512994412 | 0 | Alveolar-Mφ |
| TPT1 | 0.398650926186495 | 0 | Alveolar-Mφ |
| RP3-460G2.2 | 0.398648903921172 | 0 | Alveolar-Mφ |
| RPS4Y1 | 0.396251943942587 | 0 | Alveolar-Mφ |
| ANXA5 | 0.396053799975053 | 0 | Alveolar-Mφ |
| GGA2 | 0.394520222599308 | 0 | Alveolar-Mφ |
| STMN1 | 0.392987686368327 | 0 | Alveolar-Mφ |
| CAT | 0.392457725845791 | 0 | Alveolar-Mφ |
| MLPH | 0.390922460874552 | 0 | Alveolar-Mφ |
| SH3BGRL | 0.389409102648199 | 0 | Alveolar-Mφ |
| ALAS1 | 0.389272910063475 | 0 | Alveolar-Mφ |
| STAC | 0.387009918281304 | 0 | Alveolar-Mφ |
| MDH1 | 0.386907992264782 | 0 | Alveolar-Mφ |
| KAT8 | 0.383647540943991 | 0 | Alveolar-Mφ |
| AGPAT2 | 0.383602617976236 | 0 | Alveolar-Mφ |
| SCP2 | 0.382516585935723 | 0 | Alveolar-Mφ |
| HLA-DMA | 0.380257484781336 | 0 | Alveolar-Mφ |
| GLDN | 0.375923950675616 | 0 | Alveolar-Mφ |
| CYB5R3 | 0.375795047780518 | 0 | Alveolar-Mφ |
| TMEM173 | 0.375083950445165 | 0 | Alveolar-Mφ |
| SLC27A3 | 0.374438895952162 | 0 | Alveolar-Mφ |
| ADAM17 | 0.370802722565134 | 0 | Alveolar-Mφ |
| CD300LF | 0.370553320835571 | 0 | Alveolar-Mφ |
| PGD | 0.370079799709596 | 0 | Alveolar-Mφ |
| SELPLG | 0.367434627476911 | 0 | Alveolar-Mφ |
| TGOLN2 | 0.365821263839785 | 0 | Alveolar-Mφ |
| OAZ1 | 0.365727523573812 | 0 | Alveolar-Mφ |
| LMNA | 0.363863387944343 | 0 | Alveolar-Mφ |
| MGST1 | 0.362546309150633 | 0 | Alveolar-Mφ |
| PILRA | 0.356606073451946 | 0 | Alveolar-Mφ |
| PTPN6 | 0.356178914747859 | 0 | Alveolar-Mφ |
| LSM6 | 0.35581916412439 | 0 | Alveolar-Mφ |
| SPOCD1 | 0.353925273600659 | 0 | Alveolar-Mφ |
| RHOB | 0.353523636146764 | 0 | Alveolar-Mφ |
| PRR13 | 0.352155521132976 | 0 | Alveolar-Mφ |
| GPA33 | 0.350850046382925 | 0 | Alveolar-Mφ |
| ANXA11 | 0.350227655772628 | 0 | Alveolar-Mφ |
| RPS20 | 0.349882215496522 | 0 | Alveolar-Mφ |
| DST | 0.34839336618119 | 0 | Alveolar-Mφ |
| C19orf43 | 0.347192808387709 | 0 | Alveolar-Mφ |
| RP11-1008C21.1 | 0.346975081485315 | 0 | Alveolar-Mφ |
| POR | 0.346707583008627 | 0 | Alveolar-Mφ |
| MOB3B | 0.343403148860805 | 0 | Alveolar-Mφ |
| VAT1 | 0.342870943623102 | 0 | Alveolar-Mφ |
| HSD17B11 | 0.341487680888803 | 0 | Alveolar-Mφ |
| ENPP4 | 0.341487646842706 | 0 | Alveolar-Mφ |
| AXL | 0.338872967189682 | 0 | Alveolar-Mφ |
| CDC42EP3 | 0.338281473198537 | 0 | Alveolar-Mφ |
| HN1 | 0.337064492158281 | 0 | Alveolar-Mφ |
| SORT1 | 0.335635010253193 | 0 | Alveolar-Mφ |
| YBX1 | 0.335289600180907 | 0 | Alveolar-Mφ |
| ZBTB8OS | 0.33515086776874 | 0 | Alveolar-Mφ |
| MT-ND1 | 0.335095946811405 | 0 | Alveolar-Mφ |
| CORO2A | 0.331197665262272 | 0 | Alveolar-Mφ |
| ALDH1A1 | 0.330411027451827 | 0 | Alveolar-Mφ |
| ACER3 | 0.330084303378511 | 0 | Alveolar-Mφ |
| IRS2 | 0.329584752955319 | 0 | Alveolar-Mφ |
| MT-ND3 | 0.328946950775936 | 0 | Alveolar-Mφ |
| EML4 | 0.328674710356325 | 0 | Alveolar-Mφ |
| ECSCR | 0.328230965051092 | 0 | Alveolar-Mφ |
| CTSS | 0.327959689143063 | 0 | Alveolar-Mφ |
| TMEM230 | 0.327645923665082 | 0 | Alveolar-Mφ |
| GABARAPL1 | 0.326489809590658 | 0 | Alveolar-Mφ |
| ST13 | 0.325935796093207 | 0 | Alveolar-Mφ |
| CHP1 | 0.322171002326734 | 0 | Alveolar-Mφ |
| UBE2E2 | 0.320787188954829 | 0 | Alveolar-Mφ |
| OSBPL11 | 0.320558710615337 | 0 | Alveolar-Mφ |
| FKBP1A | 0.320458978883031 | 0 | Alveolar-Mφ |
| SGMS2 | 0.320152904926558 | 0 | Alveolar-Mφ |
| TAX1BP1 | 0.319638188978573 | 0 | Alveolar-Mφ |
| H2AFZ | 0.319024886966564 | 0 | Alveolar-Mφ |
| ECH1 | 0.31830704006016 | 0 | Alveolar-Mφ |
| DEFB1 | 0.317621401638799 | 0 | Alveolar-Mφ |
| MPC1 | 0.316212242187236 | 0 | Alveolar-Mφ |
| UBASH3B | 0.314879222785091 | 0 | Alveolar-Mφ |
| SMCO4 | 0.311183150793876 | 0 | Alveolar-Mφ |
| ANAPC15 | 0.310437333582623 | 0 | Alveolar-Mφ |
| HLA-DPB1 | 0.309108681933226 | 0 | Alveolar-Mφ |
| RASAL2 | 0.308916033094602 | 0 | Alveolar-Mφ |
| SLC15A3 | 0.30870460983488 | 0 | Alveolar-Mφ |
| CCL23 | 0.307713460120956 | 0 | Alveolar-Mφ |
| LIMA1 | 0.302900337413487 | 0 | Alveolar-Mφ |
| TMEM53 | 0.302357711343552 | 0 | Alveolar-Mφ |
| NCEH1 | 0.300010072802046 | 0 | Alveolar-Mφ |
| MSMO1 | 0.297342271371982 | 0 | Alveolar-Mφ |
| NUCB1 | 0.296784109718427 | 0 | Alveolar-Mφ |
| B3GNT5 | 0.295601777270632 | 0 | Alveolar-Mφ |
| CYB5A | 0.294058961433269 | 0 | Alveolar-Mφ |
| RASGRP2 | 0.292807155260161 | 0 | Alveolar-Mφ |
| SLFN11 | 0.29203224050518 | 0 | Alveolar-Mφ |
| TMBIM1 | 0.291925250100099 | 0 | Alveolar-Mφ |
| STX12 | 0.291558359548084 | 0 | Alveolar-Mφ |
| BLOC1S2 | 0.291320668286073 | 0 | Alveolar-Mφ |
| ROGDI | 0.29061527090455 | 0 | Alveolar-Mφ |
| MIR3945HG | 0.290500108943786 | 0 | Alveolar-Mφ |
| SARAF | 0.289682748167441 | 0 | Alveolar-Mφ |
| POLR2K | 0.289409396297907 | 0 | Alveolar-Mφ |
| C6orf48 | 0.289217030075545 | 0 | Alveolar-Mφ |
| ISCU | 0.288263788817741 | 0 | Alveolar-Mφ |
| TMED5 | 0.288132493780436 | 0 | Alveolar-Mφ |
| GPX4 | 0.287795881081324 | 0 | Alveolar-Mφ |
| RSU1 | 0.28768959948945 | 0 | Alveolar-Mφ |
| SIRPB1 | 0.285411211467803 | 0 | Alveolar-Mφ |
| ATP6V1E1 | 0.285384769979854 | 0 | Alveolar-Mφ |
| SCCPDH | 0.283647808745762 | 0 | Alveolar-Mφ |
| SPG21 | 0.282051685089479 | 0 | Alveolar-Mφ |
| CD46 | 0.281432677457688 | 0 | Alveolar-Mφ |
| HACD4 | 0.280270997518268 | 0 | Alveolar-Mφ |
| MYO6 | 0.280125552536505 | 0 | Alveolar-Mφ |
| ITPK1 | 0.279384181095353 | 0 | Alveolar-Mφ |
| GALNT12 | 0.277147737490994 | 0 | Alveolar-Mφ |
| SFTPA2 | 0.27633278088159 | 0 | Alveolar-Mφ |
| TSPAN3 | 0.275274895940029 | 0 | Alveolar-Mφ |
| MAN1A1 | 0.273586881926906 | 0 | Alveolar-Mφ |
| DIAPH1 | 0.273341425377968 | 0 | Alveolar-Mφ |
| SLC11A1 | 0.272844145998272 | 0 | Alveolar-Mφ |
| TMEM261 | 0.271995257168935 | 0 | Alveolar-Mφ |
| RTN3 | 0.271177341121341 | 0 | Alveolar-Mφ |
| DSTN | 0.269980695495745 | 0 | Alveolar-Mφ |
| TNFSF12 | 0.26937447081773 | 0 | Alveolar-Mφ |
| LSAMP | 0.265166701598564 | 0 | Alveolar-Mφ |
| GAA | 0.263170941020932 | 0 | Alveolar-Mφ |
| TAGLN | 0.262163688755414 | 0 | Alveolar-Mφ |
| NRBF2 | 0.261892125147326 | 0 | Alveolar-Mφ |
| DPYSL2 | 0.261372073092609 | 0 | Alveolar-Mφ |
| BLVRA | 0.260645201969207 | 0 | Alveolar-Mφ |
| COX17 | 0.258156648896857 | 0 | Alveolar-Mφ |
| MINK1 | 0.25699904573053 | 0 | Alveolar-Mφ |
| GPRIN3 | 0.256876959436417 | 0 | Alveolar-Mφ |
| SPNS1 | 0.254839446760631 | 0 | Alveolar-Mφ |
| C10orf128 | 0.25398841210117 | 0 | Alveolar-Mφ |
| TUBB6 | 0.253929126671532 | 0 | Alveolar-Mφ |
| PYURF | 0.253408458572076 | 0 | Alveolar-Mφ |
| RABGGTB | 0.253265920311515 | 0 | Alveolar-Mφ |
| RARA | 0.252989318489835 | 0 | Alveolar-Mφ |
| TMEM243 | 0.252901065879897 | 0 | Alveolar-Mφ |
| DDAH2 | 0.25271994628689 | 0 | Alveolar-Mφ |
| CASP1 | 0.251603945293203 | 0 | Alveolar-Mφ |
| OPN3 | 0.251124204069327 | 0 | Alveolar-Mφ |
| PTPN12 | 0.250839886715127 | 0 | Alveolar-Mφ |
| GRINA | 0.297777419369438 | 5.28244045032236e-302 | Alveolar-Mφ |
| NDUFS5 | 0.288097065729364 | 7.70620975477095e-299 | Alveolar-Mφ |
| HEXB | 0.358827754750776 | 4.13505466507088e-297 | Alveolar-Mφ |
| ARPC1B | 0.294325853492048 | 3.10569590461541e-294 | Alveolar-Mφ |
| CTSC | 0.318248828368104 | 9.9583029532595e-289 | Alveolar-Mφ |
| GLTSCR2 | 0.259544488777555 | 5.57685565013761e-286 | Alveolar-Mφ |
| CDC37 | 0.255181571945521 | 1.41020346294039e-284 | Alveolar-Mφ |
| GSTO1 | 0.335799088713327 | 1.6455275285618e-283 | Alveolar-Mφ |
| CEBPB | 0.3175688078765 | 2.53706929164992e-281 | Alveolar-Mφ |
| LAMP2 | 0.286846420408333 | 2.56264614148107e-276 | Alveolar-Mφ |
| CSF1 | 0.266709027508741 | 3.29151611931206e-272 | Alveolar-Mφ |
| ATP6V0D1 | 0.265674790491629 | 6.33522363219459e-268 | Alveolar-Mφ |
| RP11-1143G9.4 | 0.304956814787849 | 5.08317096349133e-263 | Alveolar-Mφ |
| MT-ND4 | 0.26296836197294 | 3.75531136332455e-257 | Alveolar-Mφ |
| GNB2 | 0.260840094355344 | 8.74255063326791e-252 | Alveolar-Mφ |
| HLA-DQA2 | 0.810787366130745 | 1.12177285948186e-251 | Alveolar-Mφ |
| HMGN2 | 0.265415310683488 | 8.85947498517068e-250 | Alveolar-Mφ |
| ZNF706 | 0.251356477189112 | 9.93532836184306e-250 | Alveolar-Mφ |
| NAP1L1 | 0.267661972001843 | 5.62946459758016e-247 | Alveolar-Mφ |
| FABP3 | 0.28365524360235 | 6.03828341517931e-113 | Alveolar-Mφ |
| HLA-B | 0.254018431804642 | 2.05584212124951e-63 | Alveolar-Mφ |
| IFI27 | 0.502517104754365 | 3.32989868779916e-26 | Alveolar-Mφ |
| SEPP1 | 2.61283958782613 | 0 | interstitial Mφ perivascular |
| SLC40A1 | 2.01947542962509 | 0 | interstitial Mφ perivascular |
| APOE | 1.83913051131119 | 0 | interstitial Mφ perivascular |
| LGMN | 1.52116522579186 | 0 | interstitial Mφ perivascular |
| FOLR2 | 1.51753820951958 | 0 | interstitial Mφ perivascular |
| PLTP | 1.35358610869236 | 0 | interstitial Mφ perivascular |
| MS4A6A | 1.3153185701305 | 0 | interstitial Mφ perivascular |
| TMEM176B | 1.22836251558616 | 0 | interstitial Mφ perivascular |
| F13A1 | 1.19707076670634 | 0 | interstitial Mφ perivascular |
| A2M | 1.1719395770703 | 0 | interstitial Mφ perivascular |
| FUCA1 | 1.13422389276658 | 0 | interstitial Mφ perivascular |
| PLD3 | 1.11262517663662 | 0 | interstitial Mφ perivascular |
| MARCKS | 1.10671975278491 | 0 | interstitial Mφ perivascular |
| GPNMB | 1.08884548817771 | 0 | interstitial Mφ perivascular |
| CPM | 1.00186878118777 | 0 | interstitial Mφ perivascular |
| PLA2G7 | 0.963750284254057 | 0 | interstitial Mφ perivascular |
| RARRES1 | 0.950165350873887 | 0 | interstitial Mφ perivascular |
| CTSZ | 0.93988100978067 | 0 | interstitial Mφ perivascular |
| TMEM176A | 0.928623576698786 | 0 | interstitial Mφ perivascular |
| STAB1 | 0.897371822449875 | 0 | interstitial Mφ perivascular |
| ZFP36L1 | 0.872738010473313 | 0 | interstitial Mφ perivascular |
| PSAP | 0.849455475063767 | 0 | interstitial Mφ perivascular |
| CD84 | 0.831010896601103 | 0 | interstitial Mφ perivascular |
| CTSB | 0.813044938701116 | 0 | interstitial Mφ perivascular |
| CEBPD | 0.807542219637574 | 0 | interstitial Mφ perivascular |
| CD14 | 0.803096955435651 | 0 | interstitial Mφ perivascular |
| ARL4C | 0.773467095245122 | 0 | interstitial Mφ perivascular |
| RGS1 | 0.770763365711997 | 0 | interstitial Mφ perivascular |
| NPC2 | 0.748951727496348 | 0 | interstitial Mφ perivascular |
| RNASE6 | 0.708397286863954 | 0 | interstitial Mφ perivascular |
| NPL | 0.692092907730389 | 0 | interstitial Mφ perivascular |
| GPR183 | 0.681657076152532 | 0 | interstitial Mφ perivascular |
| SAT1 | 0.663492641181997 | 0 | interstitial Mφ perivascular |
| ABCA1 | 0.66041865216115 | 0 | interstitial Mφ perivascular |
| ADAMDEC1 | 0.643650129187842 | 0 | interstitial Mφ perivascular |
| HS3ST2 | 0.569862895646452 | 0 | interstitial Mφ perivascular |
| PRCP | 0.554149258034303 | 0 | interstitial Mφ perivascular |
| LILRB5 | 0.521882513286006 | 0 | interstitial Mφ perivascular |
| SMPDL3A | 0.516807763806803 | 0 | interstitial Mφ perivascular |
| ADORA3 | 0.510527045258424 | 0 | interstitial Mφ perivascular |
| SDC3 | 0.500812911779459 | 0 | interstitial Mφ perivascular |
| TMEM37 | 0.499692079127206 | 0 | interstitial Mφ perivascular |
| GPX1 | 0.464813906586727 | 0 | interstitial Mφ perivascular |
| CMKLR1 | 0.438831732936309 | 0 | interstitial Mφ perivascular |
| CYBRD1 | 0.379885647483432 | 0 | interstitial Mφ perivascular |
| SLC18B1 | 0.369323218889748 | 0 | interstitial Mφ perivascular |
| CREG1 | 0.732979104117648 | 2.87050251883611e-303 | interstitial Mφ perivascular |
| GAS6 | 0.455667297209518 | 1.11018249122999e-301 | interstitial Mφ perivascular |
| GPR34 | 0.695832808769008 | 1.65135523260148e-301 | interstitial Mφ perivascular |
| PLXNC1 | 0.338504469933485 | 1.69426880035505e-298 | interstitial Mφ perivascular |
| LILRB4 | 0.738070074163736 | 3.78441250359166e-292 | interstitial Mφ perivascular |
| ENPP2 | 0.420007511753546 | 8.71073037330164e-292 | interstitial Mφ perivascular |
| RGS2 | 0.832177665303368 | 2.39855545722463e-291 | interstitial Mφ perivascular |
| SLC1A3 | 0.475377294879953 | 2.7619978540671e-290 | interstitial Mφ perivascular |
| LIPA | 0.999447240514184 | 1.91401948254594e-284 | interstitial Mφ perivascular |
| BASP1 | 0.508164848863463 | 1.1684011224762e-282 | interstitial Mφ perivascular |
| SDS | 0.569227655267551 | 1.88983248075627e-282 | interstitial Mφ perivascular |
| CHIT1 | 1.17687580811657 | 2.94033775053891e-276 | interstitial Mφ perivascular |
| METTL7A | 0.466757485779809 | 5.27240261209471e-272 | interstitial Mφ perivascular |
| FOS | 0.868365070374633 | 2.73288435729992e-270 | interstitial Mφ perivascular |
| FYB | 0.649180533631684 | 1.98220767198879e-268 | interstitial Mφ perivascular |
| GAL3ST4 | 0.441690050272303 | 2.28255298766253e-267 | interstitial Mφ perivascular |
| IL2RA | 0.423009040248282 | 5.01788919278852e-267 | interstitial Mφ perivascular |
| FGL2 | 0.568673826587542 | 1.63767291291104e-262 | interstitial Mφ perivascular |
| SERPINF1 | 0.644435185346674 | 1.0886190689202e-259 | interstitial Mφ perivascular |
| CCL13 | 1.44588807741372 | 1.03423404046709e-258 | interstitial Mφ perivascular |
| TPCN1 | 0.251593391486814 | 4.44979032590505e-258 | interstitial Mφ perivascular |
| OTOA | 0.525347977892614 | 2.37707124747114e-251 | interstitial Mφ perivascular |
| IER3 | 0.743956381813011 | 4.1695607722894e-251 | interstitial Mφ perivascular |
| TTYH3 | 0.512808584981764 | 4.35401062904793e-250 | interstitial Mφ perivascular |
| RASSF4 | 0.634853911770176 | 1.39875549426952e-249 | interstitial Mφ perivascular |
| RNASE1 | 0.897066005672229 | 2.16315139013008e-247 | interstitial Mφ perivascular |
| TSPAN4 | 0.6630313761389 | 3.69433072471807e-247 | interstitial Mφ perivascular |
| NINJ1 | 0.49868671299643 | 3.9172845887371e-236 | interstitial Mφ perivascular |
| HLA-DMB | 0.697251563460512 | 1.04921176062757e-232 | interstitial Mφ perivascular |
| CD209 | 0.315881879554007 | 1.0193324775851e-231 | interstitial Mφ perivascular |
| MAF | 0.624159959869723 | 3.34568001122933e-231 | interstitial Mφ perivascular |
| MERTK | 0.384006594517183 | 3.63450271572513e-220 | interstitial Mφ perivascular |
| CLEC10A | 0.411486941858514 | 5.63394222521241e-220 | interstitial Mφ perivascular |
| CFD | 0.924396145106827 | 2.53493182505e-219 | interstitial Mφ perivascular |
| FAM198B | 0.304468735812855 | 3.77258300113902e-217 | interstitial Mφ perivascular |
| CHCHD6 | 0.516706562111544 | 3.035926646327e-215 | interstitial Mφ perivascular |
| C1QC | 0.707244662025987 | 5.54613922099877e-215 | interstitial Mφ perivascular |
| FOSB | 0.886354398682559 | 1.30260833751485e-213 | interstitial Mφ perivascular |
| TCN2 | 0.628166204372677 | 2.41859290175788e-212 | interstitial Mφ perivascular |
| BLVRB | 0.642558977119762 | 1.23315993023416e-210 | interstitial Mφ perivascular |
| MAFB | 0.640210450531755 | 3.44132009773975e-209 | interstitial Mφ perivascular |
| ABCC5 | 0.304284911113795 | 2.1868044483827e-203 | interstitial Mφ perivascular |
| GPR155 | 0.257782021874157 | 2.27107104712763e-203 | interstitial Mφ perivascular |
| TMIGD3 | 0.622008133374421 | 4.27743881169594e-203 | interstitial Mφ perivascular |
| PIK3IP1 | 0.323084691762565 | 3.01989481522442e-202 | interstitial Mφ perivascular |
| FCGRT | 0.536219844529424 | 1.09365629064863e-200 | interstitial Mφ perivascular |
| SGK1 | 0.596113498917297 | 3.50747491387993e-200 | interstitial Mφ perivascular |
| HIF1A | 0.550809241564245 | 3.04553784927839e-199 | interstitial Mφ perivascular |
| CCL3 | 0.376856622453024 | 6.15464009941013e-199 | interstitial Mφ perivascular |
| GADD45B | 0.811993764909345 | 6.55846781105113e-198 | interstitial Mφ perivascular |
| OLFML2B | 0.304711403658612 | 7.35119802187567e-196 | interstitial Mφ perivascular |
| SCARB1 | 0.301720198482774 | 8.31740626889691e-196 | interstitial Mφ perivascular |
| FAM20A | 0.302702150916217 | 4.5110333353376e-195 | interstitial Mφ perivascular |
| RGL1 | 0.297430183301882 | 8.49223629514717e-193 | interstitial Mφ perivascular |
| PMP22 | 0.590471186114667 | 1.20857872804239e-190 | interstitial Mφ perivascular |
| JUNB | 0.701989385901777 | 4.67410424741922e-187 | interstitial Mφ perivascular |
| RAP2B | 0.525386094319937 | 2.1502959281525e-184 | interstitial Mφ perivascular |
| NR4A2 | 0.820684840574398 | 1.4653369288376e-183 | interstitial Mφ perivascular |
| BMP2K | 0.4617745866495 | 1.25439476126602e-179 | interstitial Mφ perivascular |
| MFSD1 | 0.544455205434688 | 1.15599513475662e-176 | interstitial Mφ perivascular |
| IL10 | 0.339656575929111 | 3.86081885836639e-172 | interstitial Mφ perivascular |
| CSF1R | 0.544835613151305 | 1.50331147795429e-171 | interstitial Mφ perivascular |
| KCNMA1 | 0.52089264309618 | 2.88161163535512e-171 | interstitial Mφ perivascular |
| MTSS1 | 0.29724411346873 | 4.28652148169402e-170 | interstitial Mφ perivascular |
| MMP14 | 0.445533136323991 | 2.26320481880405e-169 | interstitial Mφ perivascular |
| AP1B1 | 0.493129446619549 | 2.29583280757733e-169 | interstitial Mφ perivascular |
| NRP2 | 0.42609295323019 | 2.78158953591998e-168 | interstitial Mφ perivascular |
| LY96 | 0.491059845232581 | 4.14695450807069e-168 | interstitial Mφ perivascular |
| CD163 | 0.810014474120274 | 1.77350148852282e-167 | interstitial Mφ perivascular |
| ICAM1 | 0.481879414831115 | 9.04447387679847e-166 | interstitial Mφ perivascular |
| AKR1B1 | 0.650122581098534 | 8.90943028886428e-165 | interstitial Mφ perivascular |
| ADAP2 | 0.538325210400163 | 1.99958119389289e-164 | interstitial Mφ perivascular |
| C1orf54 | 0.460703654786042 | 9.96913407887413e-163 | interstitial Mφ perivascular |
| ST8SIA4 | 0.295905408320875 | 8.90291134166492e-160 | interstitial Mφ perivascular |
| EMB | 0.352734260206211 | 4.1828792271699e-158 | interstitial Mφ perivascular |
| CPVL | 0.639490253029301 | 1.51736474786246e-157 | interstitial Mφ perivascular |
| TCF4 | 0.305983814756511 | 1.0657980434829e-154 | interstitial Mφ perivascular |
| MEF2C | 0.369033540147399 | 2.57759141945107e-152 | interstitial Mφ perivascular |
| RNF130 | 0.42759786633142 | 3.40174237902697e-151 | interstitial Mφ perivascular |
| AP1S2 | 0.440193785113894 | 6.21026678874607e-151 | interstitial Mφ perivascular |
| DNPH1 | 0.389619857038953 | 2.61054369598308e-149 | interstitial Mφ perivascular |
| CYP1B1 | 0.349935401454924 | 3.68045020179724e-147 | interstitial Mφ perivascular |
| FCGR2B | 0.31695181314429 | 1.42872965740872e-145 | interstitial Mφ perivascular |
| ITGB2 | 0.485105230840237 | 1.33312558550527e-144 | interstitial Mφ perivascular |
| C3AR1 | 0.502653291216117 | 1.64040983615922e-144 | interstitial Mφ perivascular |
| MS4A4A | 0.652987785958066 | 7.7904321169533e-144 | interstitial Mφ perivascular |
| MPEG1 | 0.444458964004219 | 4.03056765767352e-143 | interstitial Mφ perivascular |
| CTSK | 1.42278841842554 | 1.42575335398934e-142 | interstitial Mφ perivascular |
| AP2A2 | 0.439849660628847 | 2.04784394740628e-142 | interstitial Mφ perivascular |
| FPR3 | 0.47942007909024 | 2.57101680647296e-142 | interstitial Mφ perivascular |
| ZNF331 | 0.590068415940975 | 4.53550165503938e-142 | interstitial Mφ perivascular |
| CTSS1 | 0.464637640755708 | 1.39548461916647e-141 | interstitial Mφ perivascular |
| EPB41L2 | 0.357613688349177 | 4.21281498009684e-140 | interstitial Mφ perivascular |
| HLA-A | 0.403075995163877 | 1.72086184990096e-138 | interstitial Mφ perivascular |
| MMP9 | 1.83100614993682 | 1.96967936086103e-138 | interstitial Mφ perivascular |
| EVA1B | 0.265271414940518 | 1.64885130528829e-137 | interstitial Mφ perivascular |
| CD48 | 0.314509311991227 | 3.6720657031993e-137 | interstitial Mφ perivascular |
| TREM2 | 0.55070610170591 | 8.61172061645946e-137 | interstitial Mφ perivascular |
| CD59 | 0.587532976772642 | 4.50751409829041e-136 | interstitial Mφ perivascular |
| ZFP36 | 0.655057402112298 | 7.76191793070638e-136 | interstitial Mφ perivascular |
| IGKC | 1.10337729187467 | 2.28147214406841e-134 | interstitial Mφ perivascular |
| IGHG3 | 0.70505870989589 | 1.63344929332136e-133 | interstitial Mφ perivascular |
| EGR1 | 0.708333581468952 | 4.26837829827102e-132 | interstitial Mφ perivascular |
| CD4 | 0.496470371985181 | 4.73351310582765e-130 | interstitial Mφ perivascular |
| HMOX1 | 0.753089760357974 | 7.71926075295111e-129 | interstitial Mφ perivascular |
| IER2 | 0.755205095767215 | 1.60330434202589e-126 | interstitial Mφ perivascular |
| HLA-E | 0.370880876225613 | 1.16638586082006e-124 | interstitial Mφ perivascular |
| RBP1 | 0.301514314106566 | 6.34051611570709e-123 | interstitial Mφ perivascular |
| AOAH | 0.258436089486163 | 1.23006334273995e-122 | interstitial Mφ perivascular |
| IGLC2 | 0.870148825354119 | 3.57787849978902e-121 | interstitial Mφ perivascular |
| LTC4S | 0.263808505738311 | 2.56203966006566e-120 | interstitial Mφ perivascular |
| GLMP | 0.368235357446393 | 8.38597801264287e-120 | interstitial Mφ perivascular |
| ASAH1 | 0.393346360506628 | 5.43964330838948e-118 | interstitial Mφ perivascular |
| RAB32 | 0.467880510251732 | 9.97457414317792e-117 | interstitial Mφ perivascular |
| FAM20C | 0.296491001777715 | 5.03666975830403e-116 | interstitial Mφ perivascular |
| SOD2 | 0.409041111913435 | 5.28500456299298e-115 | interstitial Mφ perivascular |
| NEAT1 | 0.482068256867485 | 1.50427820180901e-114 | interstitial Mφ perivascular |
| NAIP | 0.312515498052825 | 1.54224304556138e-114 | interstitial Mφ perivascular |
| DHRS9 | 0.283728896942454 | 1.54777012207315e-114 | interstitial Mφ perivascular |
| CCL4 | 0.401942118250014 | 2.00505159623195e-114 | interstitial Mφ perivascular |
| IGFBP4 | 0.272199606917296 | 2.52870826224495e-109 | interstitial Mφ perivascular |
| IDH1 | 0.467598143750665 | 3.24505140781523e-108 | interstitial Mφ perivascular |
| HERPUD1 | 0.519546612302879 | 1.63534605425185e-107 | interstitial Mφ perivascular |
| DAB2 | 0.575568800933357 | 1.0832858439881e-106 | interstitial Mφ perivascular |
| CSF3R | 0.283527383032368 | 1.23041808970475e-106 | interstitial Mφ perivascular |
| IGHG4 | 0.503684422729672 | 2.64527912889154e-106 | interstitial Mφ perivascular |
| CXCR4 | 0.332091977556864 | 1.77816523442071e-105 | interstitial Mφ perivascular |
| PFKFB3 | 0.282718595709212 | 2.36092112458823e-105 | interstitial Mφ perivascular |
| LYVE1 | 0.508291337198344 | 1.70334623957545e-104 | interstitial Mφ perivascular |
| RGS10 | 0.490559255230478 | 1.15705949008246e-103 | interstitial Mφ perivascular |
| GM2A | 0.517318649440123 | 7.63119427625997e-103 | interstitial Mφ perivascular |
| LPAR6 | 0.346939551559282 | 2.85574193420629e-102 | interstitial Mφ perivascular |
| FRMD4B | 0.348220916203618 | 1.52955615189089e-101 | interstitial Mφ perivascular |
| OAZ2 | 0.318544847478789 | 5.51374344031795e-101 | interstitial Mφ perivascular |
| CTSL | 0.328227448617536 | 1.21296926957812e-100 | interstitial Mφ perivascular |
| CHI3L1 | 0.627720996262905 | 1.98678012379439e-99 | interstitial Mφ perivascular |
| APOC1 | 0.563375117875878 | 1.11384801834485e-98 | interstitial Mφ perivascular |
| IGHG1 | 0.540528329916199 | 1.20610028096733e-97 | interstitial Mφ perivascular |
| LHFPL2 | 0.354705029126648 | 1.35657469462177e-97 | interstitial Mφ perivascular |
| FTL | 0.285615682708213 | 1.46496362393617e-97 | interstitial Mφ perivascular |
| RB1 | 0.396508770769107 | 1.78036751316688e-93 | interstitial Mφ perivascular |
| DUSP1 | 0.480819881864966 | 5.43873945087872e-93 | interstitial Mφ perivascular |
| CCL4L2 | 0.303734574848147 | 2.57786801812499e-92 | interstitial Mφ perivascular |
| TPP1 | 0.366603240067421 | 2.6293432290588e-92 | interstitial Mφ perivascular |
| SLCO2B1 | 0.510661440277291 | 8.79094136725234e-92 | interstitial Mφ perivascular |
| FCHO2 | 0.294769798592823 | 1.13130286437293e-91 | interstitial Mφ perivascular |
| CALM3 | 0.496465778658562 | 9.95383325845544e-88 | interstitial Mφ perivascular |
| MGAT4A | 0.364402813677976 | 3.92702969027148e-87 | interstitial Mφ perivascular |
| C2 | 0.446391728321362 | 6.34661652961173e-87 | interstitial Mφ perivascular |
| CXCL9 | 0.669121233286019 | 1.17076762407583e-86 | interstitial Mφ perivascular |
| HLA-DPB11 | 0.48593217927953 | 2.72036756176109e-84 | interstitial Mφ perivascular |
| PLXND1 | 0.309292304935241 | 3.90320717210449e-83 | interstitial Mφ perivascular |
| KLF6 | 0.46964665997853 | 7.48076999934366e-82 | interstitial Mφ perivascular |
| PLEKHO1 | 0.283301583559954 | 4.05777449642909e-81 | interstitial Mφ perivascular |
| EPHX1 | 0.473506601019189 | 6.64077563012304e-81 | interstitial Mφ perivascular |
| KCTD12 | 0.442718217234026 | 1.71768878778538e-80 | interstitial Mφ perivascular |
| HLA-DMA1 | 0.368004966207513 | 2.48554554792738e-80 | interstitial Mφ perivascular |
| ARL5A | 0.419976460061961 | 3.17992377379437e-80 | interstitial Mφ perivascular |
| ANKH | 0.271409979452753 | 4.19250203251862e-80 | interstitial Mφ perivascular |
| SCGB3A1 | 0.484317031910832 | 5.47016746305589e-80 | interstitial Mφ perivascular |
| IQGAP2 | 0.37725841358491 | 1.59307653232096e-79 | interstitial Mφ perivascular |
| CYFIP1 | 0.322191559208154 | 7.2987837517319e-79 | interstitial Mφ perivascular |
| SOCS3 | 0.387299194245391 | 1.00841639498507e-77 | interstitial Mφ perivascular |
| ITM2B | 0.397444990992331 | 3.27988362386928e-77 | interstitial Mφ perivascular |
| HEXA | 0.366996053743839 | 1.10198663108251e-76 | interstitial Mφ perivascular |
| CD63 | 0.335595171584425 | 1.18476231109794e-76 | interstitial Mφ perivascular |
| ADAM9 | 0.31149888501322 | 2.97503042715881e-76 | interstitial Mφ perivascular |
| CTSA | 0.392011478651461 | 8.23827171149271e-76 | interstitial Mφ perivascular |
| RP11-160E2.6 | 0.437915872645877 | 1.02246824566238e-75 | interstitial Mφ perivascular |
| MALAT1 | 0.287766762948147 | 3.82492048145872e-75 | interstitial Mφ perivascular |
| ACP2 | 0.317179921550677 | 5.29698073681714e-75 | interstitial Mφ perivascular |
| CRYL1 | 0.32908833421786 | 2.6262012025718e-74 | interstitial Mφ perivascular |
| SGPL1 | 0.260619476360744 | 2.7795956281671e-74 | interstitial Mφ perivascular |
| UCP2 | 0.372780714895831 | 1.86677182687382e-73 | interstitial Mφ perivascular |
| CREBL2 | 0.313646761309309 | 3.84298734225307e-73 | interstitial Mφ perivascular |
| IGLC3 | 0.418364604081981 | 1.10515413222398e-72 | interstitial Mφ perivascular |
| CECR1 | 0.425668060733925 | 2.47793084398296e-72 | interstitial Mφ perivascular |
| ENTPD1 | 0.295889121243617 | 2.58112083481216e-72 | interstitial Mφ perivascular |
| CD36 | 0.335786445354983 | 2.01098126023539e-71 | interstitial Mφ perivascular |
| C1QB1 | 0.402469166692828 | 4.37593854593692e-71 | interstitial Mφ perivascular |
| C3 | 0.358962701423016 | 7.26877512487741e-71 | interstitial Mφ perivascular |
| NFKBIA | 0.283780611452353 | 1.7207760328415e-70 | interstitial Mφ perivascular |
| GPX3 | 0.439296205305581 | 2.70735543374171e-70 | interstitial Mφ perivascular |
| CTSC1 | 0.460182160749245 | 4.33141644966397e-69 | interstitial Mφ perivascular |
| LGALS2 | 0.278077580850354 | 1.18942803226603e-67 | interstitial Mφ perivascular |
| ELL2 | 0.352263524466075 | 5.50794916320258e-67 | interstitial Mφ perivascular |
| FAM26F | 0.286454149433896 | 8.5017812969884e-67 | interstitial Mφ perivascular |
| ATOX1 | 0.348616793907115 | 9.02411932764694e-66 | interstitial Mφ perivascular |
| PLAU | 0.386021971298965 | 1.59178707687705e-65 | interstitial Mφ perivascular |
| MPP1 | 0.289240831716675 | 7.59346057244718e-65 | interstitial Mφ perivascular |
| NCOA4 | 0.354473014238363 | 2.08036530142116e-61 | interstitial Mφ perivascular |
| SNX6 | 0.340523421283566 | 1.61456033148935e-59 | interstitial Mφ perivascular |
| SLAMF8 | 0.263938562978477 | 5.74394898741727e-59 | interstitial Mφ perivascular |
| GNS | 0.334027695828323 | 9.46547727575894e-57 | interstitial Mφ perivascular |
| IGHA1 | 0.622824128308085 | 1.29789448757611e-54 | interstitial Mφ perivascular |
| CTSD | 0.33076938525412 | 1.8773722123645e-53 | interstitial Mφ perivascular |
| GLIPR1 | 0.335799619415317 | 5.44342311572721e-53 | interstitial Mφ perivascular |
| CYTH4 | 0.271278903728041 | 3.04011261855993e-52 | interstitial Mφ perivascular |
| TIMP2 | 0.332615069137285 | 2.72211665517926e-50 | interstitial Mφ perivascular |
| ALDH1A11 | 0.471895199905589 | 4.91659746193399e-50 | interstitial Mφ perivascular |
| FKBP5 | 0.318545917515354 | 4.67207996193662e-49 | interstitial Mφ perivascular |
| IL18 | 0.352693461328259 | 2.24702608378546e-48 | interstitial Mφ perivascular |
| SMAP2 | 0.282705532137888 | 3.43765586703453e-48 | interstitial Mφ perivascular |
| GNPDA1 | 0.260655804036689 | 1.15825267585737e-47 | interstitial Mφ perivascular |
| HSPA1A | 0.443380007324917 | 1.33615888611419e-47 | interstitial Mφ perivascular |
| NR1H3 | 0.281694119536095 | 2.65484104734547e-47 | interstitial Mφ perivascular |
| DPP7 | 0.288853999338359 | 4.3121476034404e-47 | interstitial Mφ perivascular |
| DRAM2 | 0.322099477734486 | 4.5389204542211e-47 | interstitial Mφ perivascular |
| IGSF6 | 0.469733773126706 | 4.74382757719003e-47 | interstitial Mφ perivascular |
| PRNP | 0.353922217858373 | 2.36502785887563e-46 | interstitial Mφ perivascular |
| ATP6AP2 | 0.296649223514053 | 1.6309622697647e-44 | interstitial Mφ perivascular |
| PABPC4 | 0.284467097532522 | 3.36236444614767e-44 | interstitial Mφ perivascular |
| SCPEP1 | 0.34673067404107 | 1.38678116948986e-43 | interstitial Mφ perivascular |
| C1QA1 | 0.316284047329771 | 1.59079234818991e-41 | interstitial Mφ perivascular |
| NAGK | 0.28528917311594 | 1.7075272973935e-41 | interstitial Mφ perivascular |
| DUSP2 | 0.384634970253668 | 2.87815088417617e-41 | interstitial Mφ perivascular |
| CXCL2 | 0.259074328167782 | 5.4707570816831e-41 | interstitial Mφ perivascular |
| SMS | 0.281113879342044 | 1.2100473872984e-40 | interstitial Mφ perivascular |
| HLA-DOA | 0.308913336715631 | 9.99312563177237e-40 | interstitial Mφ perivascular |
| AHR | 0.298894315263483 | 1.08984444812807e-39 | interstitial Mφ perivascular |
| NDFIP1 | 0.290229295897016 | 1.02694472902096e-38 | interstitial Mφ perivascular |
| PITHD1 | 0.269166113374868 | 1.45610475953907e-38 | interstitial Mφ perivascular |
| RAB20 | 0.255989014297787 | 3.42987750773207e-38 | interstitial Mφ perivascular |
| MT-ND31 | 0.293766967088978 | 6.0162558400071e-38 | interstitial Mφ perivascular |
| ABHD12 | 0.26920517157139 | 6.27898437486231e-37 | interstitial Mφ perivascular |
| LAPTM4A | 0.272766976032663 | 1.0458946329505e-35 | interstitial Mφ perivascular |
| TNFRSF14 | 0.256099513452681 | 1.38752980095432e-35 | interstitial Mφ perivascular |
| GADD45G | 0.253511701974558 | 3.18412932270883e-35 | interstitial Mφ perivascular |
| CHMP1B | 0.455114174621824 | 3.23705264479551e-35 | interstitial Mφ perivascular |
| MGLL | 0.38243073979134 | 1.04869949607633e-33 | interstitial Mφ perivascular |
| CST3 | 0.379587327545981 | 2.3811421180102e-33 | interstitial Mφ perivascular |
| ZFP36L2 | 0.325644297975098 | 1.47789430693726e-32 | interstitial Mφ perivascular |
| FCGR2A | 0.342649365198296 | 1.73710952106588e-32 | interstitial Mφ perivascular |
| 1-Mar | 0.2729580113507 | 7.23875051471838e-32 | interstitial Mφ perivascular |
| TBXAS1 | 0.297719520125913 | 2.93747217181347e-30 | interstitial Mφ perivascular |
| QKI | 0.273112848826359 | 4.23358881014416e-29 | interstitial Mφ perivascular |
| SOAT1 | 0.254776743148031 | 1.16561172273853e-28 | interstitial Mφ perivascular |
| ZEB2 | 0.252467267674441 | 1.6740393332266e-28 | interstitial Mφ perivascular |
| TNS3 | 0.258138742145099 | 4.72989897308611e-27 | interstitial Mφ perivascular |
| PTGDS | 0.881357183790189 | 3.49329522202126e-24 | interstitial Mφ perivascular |
| MGST2 | 0.27579476690708 | 5.63850665529902e-24 | interstitial Mφ perivascular |
| CREM | 0.346197343195245 | 6.73031205065442e-24 | interstitial Mφ perivascular |
| SIGLEC1 | 0.253393235834944 | 6.96084237715559e-24 | interstitial Mφ perivascular |
| CD741 | 0.264477502422422 | 1.53194893465211e-23 | interstitial Mφ perivascular |
| TNFAIP3 | 0.371322310893229 | 2.67496511990682e-23 | interstitial Mφ perivascular |
| EVI2A | 0.263568463830307 | 6.40412807547291e-20 | interstitial Mφ perivascular |
| VMP1 | 0.266315716594575 | 1.52660569969764e-19 | interstitial Mφ perivascular |
| SPRED1 | 0.303901351889872 | 5.34636262075657e-18 | interstitial Mφ perivascular |
| GLUL | 0.256265986599843 | 2.6010002927116e-15 | interstitial Mφ perivascular |
| BTG2 | 0.264085005635296 | 5.4589587847465e-15 | interstitial Mφ perivascular |
| CLTC | 0.264031713614607 | 3.38003364540008e-14 | interstitial Mφ perivascular |
| HLA-DQA11 | 0.31110572729608 | 3.59143187136994e-13 | interstitial Mφ perivascular |
| HLA-DPA11 | 0.270331530298105 | 3.83873308953504e-13 | interstitial Mφ perivascular |
| JUN | 0.269679898245047 | 4.78857783841482e-12 | interstitial Mφ perivascular |
| CCL181 | 1.22833026982831 | 9.95814174533822e-11 | interstitial Mφ perivascular |
| STAT1 | 0.252018760452864 | 9.95038879150164e-10 | interstitial Mφ perivascular |
| ACP51 | 0.258574461619806 | 1 | interstitial Mφ perivascular |
| AREG | 0.291449475509103 | 1 | interstitial Mφ perivascular |
| CCL2 | 3.07868879077933 | 0 | Mφ Pro-inflammatory |
| CCL3L3 | 2.41016651834602 | 0 | Mφ Pro-inflammatory |
| CXCL10 | 2.25948717912539 | 0 | Mφ Pro-inflammatory |
| CCL20 | 2.18702281550509 | 0 | Mφ Pro-inflammatory |
| CCL31 | 2.15653347950571 | 0 | Mφ Pro-inflammatory |
| CCL4L21 | 2.12417629056139 | 0 | Mφ Pro-inflammatory |
| CCL41 | 2.05980747787416 | 0 | Mφ Pro-inflammatory |
| IL1B | 1.93604732697398 | 0 | Mφ Pro-inflammatory |
| CCL7 | 1.88523426511577 | 0 | Mφ Pro-inflammatory |
| CCL8 | 1.81598372066731 | 0 | Mφ Pro-inflammatory |
| ISG15 | 1.81539560974431 | 0 | Mφ Pro-inflammatory |
| G0S2 | 1.76822984728121 | 0 | Mφ Pro-inflammatory |
| CXCL8 | 1.66101241696294 | 0 | Mφ Pro-inflammatory |
| SPP1 | 1.64088649605808 | 0 | Mφ Pro-inflammatory |
| IER31 | 1.59965125851676 | 0 | Mφ Pro-inflammatory |
| CXCL21 | 1.57740984609589 | 0 | Mφ Pro-inflammatory |
| NFKBIA1 | 1.51393392542024 | 0 | Mφ Pro-inflammatory |
| MT1X | 1.49593481693882 | 0 | Mφ Pro-inflammatory |
| TIMP1 | 1.34757473255241 | 0 | Mφ Pro-inflammatory |
| IFITM3 | 1.14225581577965 | 0 | Mφ Pro-inflammatory |
| C15orf48 | 1.14132301107474 | 0 | Mφ Pro-inflammatory |
| SOD21 | 1.12237210874776 | 0 | Mφ Pro-inflammatory |
| EREG | 1.10284129598604 | 0 | Mφ Pro-inflammatory |
| GPR1831 | 1.03224795613064 | 0 | Mφ Pro-inflammatory |
| TNFAIP31 | 0.952446277362781 | 0 | Mφ Pro-inflammatory |
| DUSP21 | 0.938619012168395 | 0 | Mφ Pro-inflammatory |
| VCAN | 0.89326117174042 | 0 | Mφ Pro-inflammatory |
| SRGN | 0.885321051285717 | 0 | Mφ Pro-inflammatory |
| CD141 | 0.8826982643633 | 0 | Mφ Pro-inflammatory |
| NFKBIZ | 0.836466073827389 | 0 | Mφ Pro-inflammatory |
| TYMP | 0.830687055872195 | 0 | Mφ Pro-inflammatory |
| ZFP36L11 | 0.827146156374232 | 0 | Mφ Pro-inflammatory |
| PLAUR | 0.823699912210091 | 0 | Mφ Pro-inflammatory |
| FAM26F1 | 0.800917588955886 | 0 | Mφ Pro-inflammatory |
| PNRC1 | 0.800836713837175 | 0 | Mφ Pro-inflammatory |
| GAPDH | 0.799944970779203 | 0 | Mφ Pro-inflammatory |
| BCL2A1 | 0.792756248148472 | 0 | Mφ Pro-inflammatory |
| PPP1R15A | 0.776923528856131 | 0 | Mφ Pro-inflammatory |
| BTG1 | 0.766025419643814 | 0 | Mφ Pro-inflammatory |
| MARCKS1 | 0.720193605975145 | 0 | Mφ Pro-inflammatory |
| LDHA | 0.700429469267758 | 0 | Mφ Pro-inflammatory |
| NINJ11 | 0.696775712480816 | 0 | Mφ Pro-inflammatory |
| ADM | 0.689128904915209 | 0 | Mφ Pro-inflammatory |
| IL4I1 | 0.675084926442601 | 0 | Mφ Pro-inflammatory |
| CLEC5A | 0.674004388114908 | 0 | Mφ Pro-inflammatory |
| TPI1 | 0.6558514894153 | 0 | Mφ Pro-inflammatory |
| CORO1A | 0.643898042986361 | 0 | Mφ Pro-inflammatory |
| ICAM11 | 0.607366524650961 | 0 | Mφ Pro-inflammatory |
| H3F3B | 0.507478498684102 | 0 | Mφ Pro-inflammatory |
| CD93 | 0.461674898513485 | 0 | Mφ Pro-inflammatory |
| NLRP3 | 0.422025442715968 | 0 | Mφ Pro-inflammatory |
| IL6 | 0.773317244382286 | 6.31937610900275e-301 | Mφ Pro-inflammatory |
| KLF61 | 0.717759464577113 | 1.21174271000936e-300 | Mφ Pro-inflammatory |
| ANKRD22 | 0.352808832579899 | 1.47393888025035e-299 | Mφ Pro-inflammatory |
| ATF5 | 0.653523398001107 | 3.53257561081321e-294 | Mφ Pro-inflammatory |
| GPR84 | 0.294545590761294 | 1.98963671855712e-287 | Mφ Pro-inflammatory |
| MT2A | 1.69836199193725 | 1.47870347571426e-279 | Mφ Pro-inflammatory |
| IL1RN | 1.02047091740898 | 7.35308305818039e-279 | Mφ Pro-inflammatory |
| PTGS2 | 0.613788828656155 | 2.19269160862254e-275 | Mφ Pro-inflammatory |
| ZFP361 | 0.725780902983547 | 1.87247867535574e-274 | Mφ Pro-inflammatory |
| CXCL1 | 1.17382782353783 | 4.20552988914554e-274 | Mφ Pro-inflammatory |
| SPHK1 | 0.281831230769881 | 5.06108889848307e-274 | Mφ Pro-inflammatory |
| MIF | 0.686994010375146 | 9.62539128886415e-274 | Mφ Pro-inflammatory |
| PIM3 | 0.546088389801246 | 1.45542493412977e-273 | Mφ Pro-inflammatory |
| MT1G | 1.86365487463135 | 1.24568604219978e-272 | Mφ Pro-inflammatory |
| SOCS31 | 0.591633444038382 | 1.00597507737071e-266 | Mφ Pro-inflammatory |
| PLEK | 0.655303790437734 | 9.07763478551566e-265 | Mφ Pro-inflammatory |
| LUCAT1 | 0.359176611413876 | 1.34691955738654e-264 | Mφ Pro-inflammatory |
| CD83 | 0.722240624775141 | 1.44938536546417e-261 | Mφ Pro-inflammatory |
| CTSB1 | 0.534288327272082 | 1.98725220929667e-260 | Mφ Pro-inflammatory |
| RSAD2 | 0.677279052224547 | 3.77821585356034e-255 | Mφ Pro-inflammatory |
| CTSL1 | 0.919356173550065 | 8.19072259115988e-253 | Mφ Pro-inflammatory |
| ENO1 | 0.540951836209488 | 7.19394808912517e-252 | Mφ Pro-inflammatory |
| ADAM8 | 0.378606909455461 | 1.63112372754564e-249 | Mφ Pro-inflammatory |
| SLC39A8 | 0.387374730647782 | 3.57660564846041e-249 | Mφ Pro-inflammatory |
| SERPINB9 | 0.355506016319543 | 1.77220110148199e-245 | Mφ Pro-inflammatory |
| CXCL3 | 1.37149291005746 | 3.33291126579185e-245 | Mφ Pro-inflammatory |
| FCGR2B1 | 0.533363982580912 | 4.12542921624425e-244 | Mφ Pro-inflammatory |
| SGK11 | 0.69572286833142 | 3.57941276428373e-242 | Mφ Pro-inflammatory |
| PLEKHO11 | 0.477494321045502 | 1.23632507435477e-238 | Mφ Pro-inflammatory |
| NFKB1 | 0.518787497865621 | 1.70269191471743e-236 | Mφ Pro-inflammatory |
| LGALS1 | 0.474994299192914 | 3.33326289166572e-232 | Mφ Pro-inflammatory |
| EPSTI1 | 0.63012453166756 | 1.17483189005776e-231 | Mφ Pro-inflammatory |
| ABL2 | 0.624047723693289 | 1.2101385382796e-231 | Mφ Pro-inflammatory |
| BNIP3 | 0.480095585426812 | 1.91568349380514e-228 | Mφ Pro-inflammatory |
| MCL1 | 0.522850019874593 | 2.55727314518254e-227 | Mφ Pro-inflammatory |
| MAFB1 | 0.547582015305997 | 1.07714444907437e-224 | Mφ Pro-inflammatory |
| SAT11 | 0.473450387750729 | 1.77378237392224e-224 | Mφ Pro-inflammatory |
| FCN1 | 0.641357363964321 | 3.83675055010389e-223 | Mφ Pro-inflammatory |
| MIR155HG | 0.432841212766566 | 7.22068978479576e-221 | Mφ Pro-inflammatory |
| TNFAIP6 | 0.405419140744035 | 4.54890861392044e-218 | Mφ Pro-inflammatory |
| CAMK1 | 0.370744055594957 | 8.82052948427508e-214 | Mφ Pro-inflammatory |
| STAB11 | 0.359700961288904 | 1.3323337959537e-213 | Mφ Pro-inflammatory |
| EGR11 | 0.756980658069786 | 4.21970244800294e-201 | Mφ Pro-inflammatory |
| PRDM1 | 0.424912298976858 | 1.00626720524831e-198 | Mφ Pro-inflammatory |
| MX2 | 0.466810959078761 | 2.1954276926162e-198 | Mφ Pro-inflammatory |
| MXD1 | 0.394564803432423 | 4.05221692991609e-198 | Mφ Pro-inflammatory |
| ISG20 | 0.589025783290876 | 3.23308163818027e-197 | Mφ Pro-inflammatory |
| LIMS1 | 0.48952870089568 | 6.23351001069454e-197 | Mφ Pro-inflammatory |
| HS3ST1 | 0.258891932930373 | 1.10231576886752e-196 | Mφ Pro-inflammatory |
| ETS2 | 0.483282972678012 | 1.62369099983025e-196 | Mφ Pro-inflammatory |
| APOBEC3A | 0.448807207929089 | 4.60968361124059e-194 | Mφ Pro-inflammatory |
| HIF1A1 | 0.484009275171256 | 7.55382426494922e-194 | Mφ Pro-inflammatory |
| MT1E | 0.91696818500279 | 3.79605096276928e-192 | Mφ Pro-inflammatory |
| SLC16A3 | 0.549313858999225 | 1.23139243619737e-191 | Mφ Pro-inflammatory |
| DUSP11 | 0.53584039325769 | 1.38874979303908e-191 | Mφ Pro-inflammatory |
| GCH1 | 0.291707004115127 | 1.48975159116556e-191 | Mφ Pro-inflammatory |
| ACTB | 0.328799665648794 | 2.5077123735023e-190 | Mφ Pro-inflammatory |
| IFI6 | 0.869294430700653 | 1.41013064977734e-185 | Mφ Pro-inflammatory |
| PHLDA1 | 0.730499223434216 | 2.63596516181323e-184 | Mφ Pro-inflammatory |
| CD300A | 0.366600986841092 | 1.20996850794863e-183 | Mφ Pro-inflammatory |
| RNASE11 | 0.685164145649834 | 1.45996532703537e-183 | Mφ Pro-inflammatory |
| EMP1 | 0.488671868723005 | 1.05761387328111e-181 | Mφ Pro-inflammatory |
| IFITM2 | 0.526989898156532 | 4.9136907690156e-181 | Mφ Pro-inflammatory |
| PKM | 0.420268793900978 | 4.66548056454186e-179 | Mφ Pro-inflammatory |
| TLR2 | 0.305972313675405 | 4.55837313281423e-178 | Mφ Pro-inflammatory |
| RNASE2 | 0.295044373839375 | 5.4685161626242e-178 | Mφ Pro-inflammatory |
| NR4A21 | 0.559280786045162 | 2.03961878628808e-177 | Mφ Pro-inflammatory |
| MT1F | 0.687737460227529 | 1.15875486535757e-176 | Mφ Pro-inflammatory |
| TNIP3 | 0.250756089236085 | 1.17180464085522e-176 | Mφ Pro-inflammatory |
| IFNGR2 | 0.431735080030564 | 1.48383904519648e-176 | Mφ Pro-inflammatory |
| NAMPT | 0.522294155551988 | 1.25401413064476e-174 | Mφ Pro-inflammatory |
| JUNB1 | 0.488679892127442 | 1.3463065672392e-174 | Mφ Pro-inflammatory |
| CFLAR | 0.471373896683569 | 9.69562330447513e-173 | Mφ Pro-inflammatory |
| PPIF | 0.454250343628248 | 1.98186525996809e-172 | Mφ Pro-inflammatory |
| SLC2A5 | 0.272537406944498 | 5.55322494572179e-172 | Mφ Pro-inflammatory |
| SEC61G | 0.414690241282143 | 4.85876837919948e-171 | Mφ Pro-inflammatory |
| GADD45B1 | 0.517286481077152 | 7.38871501992513e-170 | Mφ Pro-inflammatory |
| CD300E | 0.264699118400786 | 1.73833029732727e-168 | Mφ Pro-inflammatory |
| TNF | 0.882119614965218 | 5.81059664794914e-165 | Mφ Pro-inflammatory |
| LILRB41 | 0.426385128488127 | 1.78327192815063e-163 | Mφ Pro-inflammatory |
| IFI44L | 0.583956790880267 | 8.09320180816393e-163 | Mφ Pro-inflammatory |
| FOSB1 | 0.507010493038414 | 8.83363573459936e-163 | Mφ Pro-inflammatory |
| RNASET2 | 0.594597457752097 | 1.01963320323938e-160 | Mφ Pro-inflammatory |
| RGS21 | 0.545095955142714 | 1.160924363933e-160 | Mφ Pro-inflammatory |
| CST6 | 0.281704080611072 | 1.0501562553994e-159 | Mφ Pro-inflammatory |
| NR4A3 | 0.418104321885192 | 8.26570753624679e-158 | Mφ Pro-inflammatory |
| IER21 | 0.58144868814306 | 1.38871430023967e-156 | Mφ Pro-inflammatory |
| SLC2A3 | 0.477104204885501 | 3.23361563097201e-156 | Mφ Pro-inflammatory |
| CARD19 | 0.312684356733109 | 1.17555625317386e-155 | Mφ Pro-inflammatory |
| PGAM1 | 0.420800188827833 | 1.66225392068593e-153 | Mφ Pro-inflammatory |
| ABCA11 | 0.338674188237535 | 6.85116371267169e-153 | Mφ Pro-inflammatory |
| VEGFA | 0.328608253922855 | 6.10297725957777e-152 | Mφ Pro-inflammatory |
| CDKN1A | 0.45590659698368 | 3.22955957985104e-151 | Mφ Pro-inflammatory |
| CSF1R1 | 0.397494005047905 | 5.82783940699021e-151 | Mφ Pro-inflammatory |
| PGK1 | 0.440443905936514 | 6.79490152991483e-150 | Mφ Pro-inflammatory |
| BASP11 | 0.366433970769704 | 8.45083133391572e-150 | Mφ Pro-inflammatory |
| PKIB | 0.252555963237323 | 1.75112012623042e-149 | Mφ Pro-inflammatory |
| AP1S21 | 0.401537226519063 | 3.49447036518373e-149 | Mφ Pro-inflammatory |
| CALR | 0.500719303878193 | 1.85494468162197e-147 | Mφ Pro-inflammatory |
| C4orf3 | 0.383967214381209 | 1.4250146281208e-146 | Mφ Pro-inflammatory |
| FNIP2 | 0.375288521371191 | 3.48653026180637e-146 | Mφ Pro-inflammatory |
| ZEB21 | 0.390524022118686 | 1.25393572547763e-144 | Mφ Pro-inflammatory |
| CSRNP1 | 0.379978229702673 | 1.11409192570489e-143 | Mφ Pro-inflammatory |
| PMAIP1 | 0.391552705559987 | 2.93958719636429e-142 | Mφ Pro-inflammatory |
| PLSCR1 | 0.440318760357675 | 7.52491821542286e-142 | Mφ Pro-inflammatory |
| RGS11 | 0.85025287106088 | 1.93118137738353e-141 | Mφ Pro-inflammatory |
| OSM | 0.438689581868223 | 2.45964161555789e-141 | Mφ Pro-inflammatory |
| TUBA1C | 0.404237149321661 | 3.7985668093328e-141 | Mφ Pro-inflammatory |
| KDM6B | 0.458903714998603 | 4.38631969766283e-141 | Mφ Pro-inflammatory |
| FAM49B | 0.346866570081844 | 1.7206076964525e-140 | Mφ Pro-inflammatory |
| P4HB | 0.38095854117834 | 3.5983293414695e-139 | Mφ Pro-inflammatory |
| MAP3K8 | 0.458241272629417 | 4.62600944057428e-139 | Mφ Pro-inflammatory |
| CD481 | 0.289890140613821 | 1.13224912813935e-137 | Mφ Pro-inflammatory |
| TGFBI | 0.539787565340059 | 5.80168809321644e-137 | Mφ Pro-inflammatory |
| FGL21 | 0.361761790990818 | 1.91714676398217e-134 | Mφ Pro-inflammatory |
| PDE4B | 0.320772510988899 | 5.53677244058754e-134 | Mφ Pro-inflammatory |
| DUSP6 | 0.464402287130884 | 1.23232977587417e-133 | Mφ Pro-inflammatory |
| IRF7 | 0.420832083382629 | 3.58277738780022e-133 | Mφ Pro-inflammatory |
| HMGA1 | 0.393174668552119 | 4.73525123760232e-133 | Mφ Pro-inflammatory |
| TNFSF13B | 0.529612982604791 | 2.61246701551345e-132 | Mφ Pro-inflammatory |
| SLAMF9 | 0.301902789593921 | 2.78268554344719e-132 | Mφ Pro-inflammatory |
| SCO2 | 0.387025308326632 | 3.52547534448686e-132 | Mφ Pro-inflammatory |
| LAP3 | 0.431460947501954 | 5.03964477937443e-132 | Mφ Pro-inflammatory |
| GRB2 | 0.366994551493374 | 2.35268475065205e-131 | Mφ Pro-inflammatory |
| REL | 0.500103874406641 | 1.69819139507852e-130 | Mφ Pro-inflammatory |
| TPM4 | 0.362627073215313 | 4.30188455545446e-130 | Mφ Pro-inflammatory |
| NEAT11 | 0.352660877396317 | 6.95053214366343e-130 | Mφ Pro-inflammatory |
| FLOT1 | 0.343308990276599 | 3.91135989678264e-125 | Mφ Pro-inflammatory |
| C10orf54 | 0.347372476085771 | 8.36799483062733e-123 | Mφ Pro-inflammatory |
| CCR1 | 0.408568564520762 | 1.4517725723357e-121 | Mφ Pro-inflammatory |
| IL101 | 0.325011979566305 | 1.81857327184768e-121 | Mφ Pro-inflammatory |
| METRNL | 0.341021842476627 | 1.13861116698781e-120 | Mφ Pro-inflammatory |
| AQP9 | 0.390692239283516 | 1.71801533571103e-120 | Mφ Pro-inflammatory |
| AP2S1 | 0.292671785386522 | 8.74062350987107e-120 | Mφ Pro-inflammatory |
| ADAP21 | 0.374557415744386 | 3.22493412000177e-119 | Mφ Pro-inflammatory |
| MEF2C1 | 0.283098940918904 | 1.46321612343531e-118 | Mφ Pro-inflammatory |
| SLC25A37 | 0.372231591944306 | 3.82306479723196e-117 | Mφ Pro-inflammatory |
| OTUD1 | 0.527552357075326 | 1.06423028267843e-115 | Mφ Pro-inflammatory |
| SH3BGRL3 | 0.286276861922483 | 3.40493239816657e-115 | Mφ Pro-inflammatory |
| FPR31 | 0.378702405832935 | 2.86632630340859e-114 | Mφ Pro-inflammatory |
| ITGB21 | 0.33628359704358 | 6.23740521252231e-114 | Mφ Pro-inflammatory |
| PPIB | 0.373320921919527 | 1.66368115563487e-113 | Mφ Pro-inflammatory |
| CAPZB | 0.274803979402363 | 4.78518743057781e-113 | Mφ Pro-inflammatory |
| STAT11 | 0.484468685902974 | 1.41279811396614e-112 | Mφ Pro-inflammatory |
| LYN | 0.408412803282285 | 2.36348744762654e-111 | Mφ Pro-inflammatory |
| ZC3H12A | 0.288732224213303 | 1.85549602881491e-110 | Mφ Pro-inflammatory |
| TNFRSF1B | 0.298707896036845 | 1.57046555084092e-109 | Mφ Pro-inflammatory |
| MARCKSL1 | 0.260204576638005 | 1.93092633565861e-109 | Mφ Pro-inflammatory |
| TTYH31 | 0.288464942208258 | 2.07628437891919e-109 | Mφ Pro-inflammatory |
| PTPRE | 0.323651865331727 | 4.49484969771655e-107 | Mφ Pro-inflammatory |
| ATF3 | 0.394451902323409 | 5.33467893828836e-107 | Mφ Pro-inflammatory |
| DSE | 0.321624562556763 | 5.81774378647075e-107 | Mφ Pro-inflammatory |
| TAP1 | 0.38861865589978 | 2.25826022060412e-105 | Mφ Pro-inflammatory |
| MALAT11 | 0.295038060026959 | 6.89927306500048e-105 | Mφ Pro-inflammatory |
| VAMP5 | 0.791952111287549 | 1.41573439801183e-100 | Mφ Pro-inflammatory |
| RIPK2 | 0.31087196391993 | 2.23251579114462e-100 | Mφ Pro-inflammatory |
| TAGAP | 0.257413513575808 | 2.97322312354882e-100 | Mφ Pro-inflammatory |
| ERO1A | 0.372919866607443 | 8.65519400318572e-100 | Mφ Pro-inflammatory |
| MX1 | 0.606054282856634 | 1.6151355508622e-99 | Mφ Pro-inflammatory |
| SLC43A3 | 0.301850492312455 | 3.66923548024856e-99 | Mφ Pro-inflammatory |
| PVRL2 | 0.265166768571351 | 2.25371198977637e-98 | Mφ Pro-inflammatory |
| GALM | 0.253004339675239 | 3.38689393991254e-98 | Mφ Pro-inflammatory |
| SAMSN1 | 0.334121661616655 | 2.33492669095269e-97 | Mφ Pro-inflammatory |
| KLF10 | 0.335771813863364 | 1.2667318806167e-96 | Mφ Pro-inflammatory |
| CCDC109B | 0.308406987810608 | 5.24830598048607e-95 | Mφ Pro-inflammatory |
| CKS2 | 0.320394792797437 | 1.01428636329846e-94 | Mφ Pro-inflammatory |
| IFI35 | 0.4355616178594 | 1.75234470218259e-94 | Mφ Pro-inflammatory |
| CMPK2 | 0.27240151550189 | 2.00880510342662e-94 | Mφ Pro-inflammatory |
| PEA15 | 0.282017866134191 | 3.34357379660419e-94 | Mφ Pro-inflammatory |
| BST2 | 0.412376720460177 | 1.15668273096823e-93 | Mφ Pro-inflammatory |
| AREG1 | 0.79636488306574 | 2.28463751292766e-93 | Mφ Pro-inflammatory |
| USP18 | 0.269660011318568 | 1.02014297242028e-92 | Mφ Pro-inflammatory |
| PDIA6 | 0.356196519884123 | 1.02857361303691e-92 | Mφ Pro-inflammatory |
| DDIT4 | 0.293470321759149 | 3.01071442920673e-92 | Mφ Pro-inflammatory |
| SLC16A10 | 0.255187554663511 | 1.41065296498544e-91 | Mφ Pro-inflammatory |
| PDIA3 | 0.312464053447193 | 8.97088149304488e-91 | Mφ Pro-inflammatory |
| LINC00936 | 0.403030556911796 | 9.6001416579625e-91 | Mφ Pro-inflammatory |
| C3AR11 | 0.270436768145452 | 1.61884928294864e-90 | Mφ Pro-inflammatory |
| KDELR2 | 0.313767191669618 | 1.27541964458564e-89 | Mφ Pro-inflammatory |
| ID2 | 0.459852801209933 | 1.19207925520129e-88 | Mφ Pro-inflammatory |
| TNFAIP8 | 0.291421511845752 | 1.21552378405954e-87 | Mφ Pro-inflammatory |
| EMP3 | 0.37683855960834 | 2.46718122653264e-87 | Mφ Pro-inflammatory |
| NR4A1 | 0.385179275076142 | 3.20138438050764e-87 | Mφ Pro-inflammatory |
| SDF2L1 | 0.448090025687616 | 3.97789559874938e-87 | Mφ Pro-inflammatory |
| TMEM167A | 0.30069181736359 | 5.4259615914032e-85 | Mφ Pro-inflammatory |
| RALA | 0.343216985060346 | 1.15518366644097e-84 | Mφ Pro-inflammatory |
| KCTD121 | 0.313717959473929 | 1.42549430140283e-83 | Mφ Pro-inflammatory |
| ALDOA | 0.284374569086724 | 3.01739399074369e-83 | Mφ Pro-inflammatory |
| LCP2 | 0.321947083336654 | 4.66550977226718e-83 | Mφ Pro-inflammatory |
| IFIT1 | 0.820061336571551 | 4.6853962191429e-83 | Mφ Pro-inflammatory |
| OAS2 | 0.346814501881707 | 6.97412499750204e-83 | Mφ Pro-inflammatory |
| ARID5B | 0.272170284816715 | 7.72420325277805e-83 | Mφ Pro-inflammatory |
| PLK3 | 0.303687153245728 | 4.46537809956549e-81 | Mφ Pro-inflammatory |
| EHD1 | 0.255735221505314 | 8.95697801267607e-81 | Mφ Pro-inflammatory |
| BIRC3 | 0.25737007883669 | 2.88732111823366e-80 | Mφ Pro-inflammatory |
| OAS3 | 0.329021114337872 | 4.17778239618986e-80 | Mφ Pro-inflammatory |
| NFKBID | 0.269673782889604 | 1.04128467313203e-79 | Mφ Pro-inflammatory |
| FOS1 | 0.469262838090135 | 1.47234501021429e-78 | Mφ Pro-inflammatory |
| CEBPD1 | 0.303783460449596 | 3.56232990153792e-76 | Mφ Pro-inflammatory |
| MAP2K3 | 0.265176208749023 | 5.160072465991e-76 | Mφ Pro-inflammatory |
| HM13 | 0.292206037575343 | 7.51020839411564e-76 | Mφ Pro-inflammatory |
| CLEC7A | 0.296970864809908 | 1.8973051379362e-75 | Mφ Pro-inflammatory |
| FNDC3B | 0.331816280878149 | 5.85065645360859e-75 | Mφ Pro-inflammatory |
| DRAM1 | 0.332066645317674 | 1.95551578134316e-74 | Mφ Pro-inflammatory |
| DRAP1 | 0.283800616859375 | 2.11182438436991e-73 | Mφ Pro-inflammatory |
| PMP221 | 0.27800376322076 | 1.21090927676702e-72 | Mφ Pro-inflammatory |
| BAZ1A | 0.326768201140713 | 4.30369068431284e-72 | Mφ Pro-inflammatory |
| WSB1 | 0.295112918075237 | 1.02287493692983e-71 | Mφ Pro-inflammatory |
| SLC25A19 | 0.311097987821796 | 1.23679662875796e-71 | Mφ Pro-inflammatory |
| NME1 | 0.313664026090627 | 2.66350140749837e-71 | Mφ Pro-inflammatory |
| IFI44 | 0.333309523613883 | 1.29708011925254e-70 | Mφ Pro-inflammatory |
| NDRG1 | 0.267168233821556 | 7.28115246791219e-70 | Mφ Pro-inflammatory |
| XAF1 | 0.354747742064606 | 8.42237954475924e-70 | Mφ Pro-inflammatory |
| CREM1 | 0.278737157475485 | 6.64723623689735e-69 | Mφ Pro-inflammatory |
| GBP1 | 0.614796442407937 | 7.60704449700038e-69 | Mφ Pro-inflammatory |
| IRF2BP2 | 0.273209377077578 | 3.19437103126075e-68 | Mφ Pro-inflammatory |
| S100A9 | 0.509828941226731 | 3.91145605171526e-68 | Mφ Pro-inflammatory |
| WTAP | 0.336952658644003 | 3.99837466278098e-68 | Mφ Pro-inflammatory |
| SDS1 | 0.277255967381946 | 9.26424689252211e-68 | Mφ Pro-inflammatory |
| RHOC | 0.265741132770736 | 1.43638618064985e-67 | Mφ Pro-inflammatory |
| PSMB9 | 0.376457654761347 | 1.36653389479064e-65 | Mφ Pro-inflammatory |
| GLUL1 | 0.311962187615534 | 5.35550707324964e-65 | Mφ Pro-inflammatory |
| MANF | 0.354960668720269 | 5.67799836573482e-65 | Mφ Pro-inflammatory |
| ME2 | 0.322443278703803 | 7.2871843290394e-65 | Mφ Pro-inflammatory |
| PLAU1 | 0.519514956976596 | 1.10201896233077e-64 | Mφ Pro-inflammatory |
| GNA15 | 0.251912723427784 | 2.95592007181736e-63 | Mφ Pro-inflammatory |
| FYB1 | 0.277957081306228 | 4.02988032724912e-63 | Mφ Pro-inflammatory |
| NGFRAP1 | 0.257476826249698 | 6.11772890177237e-63 | Mφ Pro-inflammatory |
| RPL22L1 | 0.344647551951128 | 1.65374987664449e-62 | Mφ Pro-inflammatory |
| SFPQ | 0.275434113455508 | 1.16046831120236e-61 | Mφ Pro-inflammatory |
| MAFF | 0.320616926581229 | 4.47200261892844e-61 | Mφ Pro-inflammatory |
| FCGR2A1 | 0.33357594145339 | 1.17947629436884e-58 | Mφ Pro-inflammatory |
| TUBB4B | 0.258409159017377 | 2.96105595555382e-58 | Mφ Pro-inflammatory |
| S100A8 | 0.666829877347343 | 8.15680017808979e-58 | Mφ Pro-inflammatory |
| EIF4E | 0.373440207329689 | 1.35751127428674e-56 | Mφ Pro-inflammatory |
| IFIT3 | 0.688522383841802 | 1.67463488225554e-56 | Mφ Pro-inflammatory |
| NRP21 | 0.252251906546173 | 1.96094795634672e-56 | Mφ Pro-inflammatory |
| IL1A | 0.310602248725912 | 4.44897132269364e-56 | Mφ Pro-inflammatory |
| LINC00152 | 0.257069352463781 | 8.66300725239534e-56 | Mφ Pro-inflammatory |
| CD1631 | 0.28048955336986 | 2.86432737926298e-54 | Mφ Pro-inflammatory |
| IRF1 | 0.388164084431313 | 1.25147026173173e-53 | Mφ Pro-inflammatory |
| LSP1 | 0.272682722382021 | 2.77944624333721e-53 | Mφ Pro-inflammatory |
| TMEM123 | 0.262575092708795 | 3.81434454337355e-53 | Mφ Pro-inflammatory |
| GBP5 | 0.292512174196866 | 9.57938820747159e-53 | Mφ Pro-inflammatory |
| TNFSF10 | 0.573721589593902 | 3.30992629252354e-52 | Mφ Pro-inflammatory |
| TRIB1 | 0.286197922061819 | 6.46993521014086e-49 | Mφ Pro-inflammatory |
| ZNF3311 | 0.266349400357004 | 1.35501190157854e-48 | Mφ Pro-inflammatory |
| RCAN1 | 0.256407099511255 | 2.66571072661035e-47 | Mφ Pro-inflammatory |
| RP11-160E2.61 | 0.320221137787126 | 3.18643779392935e-47 | Mφ Pro-inflammatory |
| HAMP | 0.308414697338224 | 3.41232146834542e-47 | Mφ Pro-inflammatory |
| CD47 | 0.254261499322838 | 6.33665369588967e-47 | Mφ Pro-inflammatory |
| ANPEP | 0.261169398534511 | 4.87452533163772e-45 | Mφ Pro-inflammatory |
| UPP1 | 0.270879900246297 | 2.08212924857828e-44 | Mφ Pro-inflammatory |
| HSPA5 | 0.329076244077173 | 8.08851679614114e-44 | Mφ Pro-inflammatory |
| ANKRD28 | 0.275109123472482 | 1.42707477891697e-43 | Mφ Pro-inflammatory |
| ENG | 0.255571608104889 | 1.82526225582923e-42 | Mφ Pro-inflammatory |
| HCST | 0.268743339836823 | 2.85296579045021e-42 | Mφ Pro-inflammatory |
| BTG21 | 0.276631643759277 | 9.97581273623674e-41 | Mφ Pro-inflammatory |
| PHLDA2 | 0.261755820858167 | 7.2062286801817e-40 | Mφ Pro-inflammatory |
| EIF2AK2 | 0.272500071230259 | 7.41659867490598e-39 | Mφ Pro-inflammatory |
| RNF213 | 0.29113491000644 | 1.16009881200609e-36 | Mφ Pro-inflammatory |
| UBE2L6 | 0.267822728988839 | 2.04791509188696e-35 | Mφ Pro-inflammatory |
| TWISTNB | 0.392728844922697 | 1.53671910610891e-34 | Mφ Pro-inflammatory |
| OAS1 | 0.319746290370682 | 7.5716123557118e-33 | Mφ Pro-inflammatory |
| THBD | 0.343223953420717 | 1.03012507021006e-27 | Mφ Pro-inflammatory |
| PLIN2 | 0.545404959842816 | 9.61950568428068e-27 | Mφ Pro-inflammatory |
| TREM21 | 0.315257218722787 | 2.16772699083233e-23 | Mφ Pro-inflammatory |
| PARP14 | 0.251553549833067 | 1.14566906118238e-21 | Mφ Pro-inflammatory |
| IER5 | 0.273993067089122 | 2.34077050803379e-20 | Mφ Pro-inflammatory |
| IFIT2 | 0.596526923909431 | 9.51965083397016e-17 | Mφ Pro-inflammatory |
| LY6E1 | 0.265314791814441 | 1.36180456275558e-13 | Mφ Pro-inflammatory |
| MIR3945HG1 | 0.371299771592327 | 1.43994505954375e-12 | Mφ Pro-inflammatory |
| DNAJB1 | 0.268352144284034 | 2.2195685362261e-11 | Mφ Pro-inflammatory |
| EGR2 | 0.269684372660467 | 4.12333434221896e-11 | Mφ Pro-inflammatory |
| NT5C3A | 0.251571378377886 | 1 | Mφ Pro-inflammatory |
| SPP11 | 1.78956137703732 | 0 | Mφ Anti-inflammatory |
| GAPDH1 | 0.857845977131251 | 0 | Mφ Anti-inflammatory |
| RPL13 | 0.439435564857711 | 4.54377592272137e-244 | Mφ Anti-inflammatory |
| RPL36 | 0.430647719786696 | 9.82308067572471e-201 | Mφ Anti-inflammatory |
| RPS6 | 0.481826888902458 | 1.35959224076157e-188 | Mφ Anti-inflammatory |
| RPS18 | 0.398934151645676 | 4.09190361222615e-182 | Mφ Anti-inflammatory |
| RPS3A | 0.41702155663745 | 1.139512473501e-172 | Mφ Anti-inflammatory |
| RPL35 | 0.351004060913757 | 4.95959677240224e-163 | Mφ Anti-inflammatory |
| RPL35A | 0.425654705864607 | 5.40946503058099e-155 | Mφ Anti-inflammatory |
| RPL24 | 0.364994265385039 | 1.21735948814416e-150 | Mφ Anti-inflammatory |
| RPL41 | 0.366445361661991 | 1.43816311995262e-150 | Mφ Anti-inflammatory |
| TPI11 | 0.696638098332546 | 1.62730477115811e-147 | Mφ Anti-inflammatory |
| RPL29 | 0.359742769645066 | 5.10834583962834e-144 | Mφ Anti-inflammatory |
| RPS15 | 0.30563503268982 | 6.10166165045032e-138 | Mφ Anti-inflammatory |
| RPL31 | 0.450628457002422 | 2.70117470585281e-137 | Mφ Anti-inflammatory |
| RPS15A | 0.411428470642941 | 2.41930783256436e-136 | Mφ Anti-inflammatory |
| RPS7 | 0.385722005970471 | 6.07802413703245e-136 | Mφ Anti-inflammatory |
| SLC2A1 | 0.593270194230061 | 1.72677597475548e-129 | Mφ Anti-inflammatory |
| CORO1A1 | 0.742948289365628 | 1.64829523725556e-123 | Mφ Anti-inflammatory |
| RPL32 | 0.346416771883105 | 6.60684792708426e-123 | Mφ Anti-inflammatory |
| PTMA | 0.335352525021686 | 1.29284922832144e-119 | Mφ Anti-inflammatory |
| ENO11 | 0.618325121736637 | 3.38392848892629e-119 | Mφ Anti-inflammatory |
| RPS4X | 0.390439856003978 | 4.3180026595756e-119 | Mφ Anti-inflammatory |
| LDHA1 | 0.789364765495661 | 8.75655309700072e-117 | Mφ Anti-inflammatory |
| RPL18 | 0.334091057360325 | 4.30318097227488e-115 | Mφ Anti-inflammatory |
| RPS17 | 0.329216574722645 | 3.5493968821489e-112 | Mφ Anti-inflammatory |
| RPL37 | 0.337654776301647 | 2.68769311964777e-102 | Mφ Anti-inflammatory |
| RPS3 | 0.405391318036094 | 3.01817519481318e-102 | Mφ Anti-inflammatory |
| RPL39 | 0.310218502458719 | 1.24203874393029e-100 | Mφ Anti-inflammatory |
| LGALS11 | 0.47451228791716 | 1.82809417560528e-99 | Mφ Anti-inflammatory |
| RPL10 | 0.311046423578575 | 2.02465969034718e-99 | Mφ Anti-inflammatory |
| RPL18A | 0.315428917305732 | 2.11146707745556e-97 | Mφ Anti-inflammatory |
| RPLP2 | 0.286757924098578 | 6.76939932132585e-96 | Mφ Anti-inflammatory |
| RPL23A | 0.364361996036376 | 1.09168618178942e-92 | Mφ Anti-inflammatory |
| RPS12 | 0.311630064014769 | 7.08100157667499e-91 | Mφ Anti-inflammatory |
| BNIP31 | 0.711501739155047 | 1.24251349153068e-90 | Mφ Anti-inflammatory |
| RPS27A | 0.331378687103584 | 1.50266593030955e-88 | Mφ Anti-inflammatory |
| RPL13A | 0.309301889879749 | 4.95783308210205e-88 | Mφ Anti-inflammatory |
| RPLP0 | 0.394557963256243 | 1.99398815764075e-87 | Mφ Anti-inflammatory |
| RPL27A | 0.25589933708478 | 6.56566873702467e-84 | Mφ Anti-inflammatory |
| MIF1 | 0.820771832633044 | 1.06722186785384e-83 | Mφ Anti-inflammatory |
| ACAP1 | 0.296958172919326 | 2.44188222511699e-83 | Mφ Anti-inflammatory |
| ADAM81 | 0.419508025315075 | 5.27983949458026e-82 | Mφ Anti-inflammatory |
| RPL11 | 0.279376782644557 | 9.54129665688377e-81 | Mφ Anti-inflammatory |
| RPS25 | 0.331266937634848 | 2.73612764935421e-80 | Mφ Anti-inflammatory |
| RPS8 | 0.329710864708938 | 9.23817795489199e-78 | Mφ Anti-inflammatory |
| RNASE12 | 0.819614332302394 | 9.46953566533858e-75 | Mφ Anti-inflammatory |
| SCGB3A11 | 0.855839877308138 | 6.08933485696997e-70 | Mφ Anti-inflammatory |
| RPL26 | 0.284301514583334 | 4.42518429558123e-69 | Mφ Anti-inflammatory |
| RPL34 | 0.338488051815263 | 9.88994774265939e-65 | Mφ Anti-inflammatory |
| RPL9 | 0.255578262797757 | 5.61664609148952e-64 | Mφ Anti-inflammatory |
| TMA7 | 0.403839973211997 | 9.69871849217151e-62 | Mφ Anti-inflammatory |
| SLAMF91 | 0.388715442884731 | 8.37700883286169e-60 | Mφ Anti-inflammatory |
| RPL22 | 0.26183932978753 | 2.91926528976489e-59 | Mφ Anti-inflammatory |
| RPSA | 0.384823709839637 | 3.196047273854e-59 | Mφ Anti-inflammatory |
| RPL7 | 0.296493127138659 | 1.22454951523342e-58 | Mφ Anti-inflammatory |
| RPS29 | 0.322354870782707 | 3.64273217806791e-58 | Mφ Anti-inflammatory |
| FCGR2B2 | 0.557562676504839 | 6.63536625502004e-55 | Mφ Anti-inflammatory |
| RPL21 | 0.292486190908418 | 8.0079558733854e-54 | Mφ Anti-inflammatory |
| TRAC | 0.743705166309635 | 5.47649556200682e-53 | Mφ Anti-inflammatory |
| RPL7A | 0.258463380808751 | 5.84632127105347e-53 | Mφ Anti-inflammatory |
| DDIT41 | 0.559400537766772 | 2.46167744725239e-52 | Mφ Anti-inflammatory |
| RPL3 | 0.369575686592778 | 2.12796943750284e-50 | Mφ Anti-inflammatory |
| RPS27 | 0.38247444707159 | 3.62027774534719e-50 | Mφ Anti-inflammatory |
| NUPR1 | 0.464318744366754 | 5.38011986882032e-49 | Mφ Anti-inflammatory |
| SLC2A51 | 0.28698067517943 | 1.54955124861106e-48 | Mφ Anti-inflammatory |
| RPL30 | 0.283419328896867 | 2.61713458067942e-48 | Mφ Anti-inflammatory |
| VCAN1 | 0.559618786543845 | 2.1577714785118e-45 | Mφ Anti-inflammatory |
| CD482 | 0.472203361931252 | 1.01168579249397e-44 | Mφ Anti-inflammatory |
| RPL10A | 0.299293454296341 | 1.13522178003622e-42 | Mφ Anti-inflammatory |
| PLIN21 | 0.426794063527331 | 5.9020805402192e-41 | Mφ Anti-inflammatory |
| ALDOA1 | 0.330993910025585 | 5.72677580441103e-39 | Mφ Anti-inflammatory |
| LIMD2 | 0.397435531012727 | 2.19739968050643e-37 | Mφ Anti-inflammatory |
| S100B | 0.357436854043581 | 1.44270954808445e-36 | Mφ Anti-inflammatory |
| RPL6 | 0.261937343420854 | 7.14255334136674e-36 | Mφ Anti-inflammatory |
| PKIB1 | 0.258196856855751 | 1.05548964471502e-33 | Mφ Anti-inflammatory |
| C4orf31 | 0.490642023451565 | 3.49448801507077e-33 | Mφ Anti-inflammatory |
| CLEC5A1 | 0.293992457173955 | 7.54192960983072e-33 | Mφ Anti-inflammatory |
| RPL36A | 0.464692054830767 | 3.29236459790306e-32 | Mφ Anti-inflammatory |
| MT1X1 | 0.819379607701193 | 1.32358223292143e-31 | Mφ Anti-inflammatory |
| NDRG11 | 0.542552791459276 | 3.22697303984994e-31 | Mφ Anti-inflammatory |
| HSPA1A1 | 0.339820746070896 | 1.22083276082658e-29 | Mφ Anti-inflammatory |
| UBC | 0.340656622167626 | 7.06068076283208e-28 | Mφ Anti-inflammatory |
| CD2 | 0.388607454505177 | 1.59475986686561e-27 | Mφ Anti-inflammatory |
| GNLY | 0.284668774268182 | 5.79075451872672e-27 | Mφ Anti-inflammatory |
| TMIGD31 | 0.50360006830829 | 8.38814781061699e-25 | Mφ Anti-inflammatory |
| RPL5 | 0.286568985507267 | 4.61628479723059e-24 | Mφ Anti-inflammatory |
| BTG11 | 0.472776456782389 | 1.29183156807081e-22 | Mφ Anti-inflammatory |
| TIMP11 | 0.704584415797804 | 2.76048599884226e-22 | Mφ Anti-inflammatory |
| SEC61G1 | 0.397589011529941 | 1.75277368520572e-21 | Mφ Anti-inflammatory |
| IL4I11 | 0.388544430900373 | 8.50788285515975e-21 | Mφ Anti-inflammatory |
| CAMK11 | 0.296779100495693 | 2.88556204125253e-20 | Mφ Anti-inflammatory |
| IGKC1 | 0.437906371932365 | 1.16982991984185e-19 | Mφ Anti-inflammatory |
| CXCR41 | 0.675618426955336 | 2.34445614607018e-19 | Mφ Anti-inflammatory |
| OST4 | 0.263562901169951 | 3.14066545178429e-19 | Mφ Anti-inflammatory |
| IFITM21 | 0.488628800086995 | 1.73696087964565e-18 | Mφ Anti-inflammatory |
| EEF1B2 | 0.273482554077689 | 3.06618961238102e-17 | Mφ Anti-inflammatory |
| TRBC2 | 0.431600612877465 | 4.87596052025027e-17 | Mφ Anti-inflammatory |
| PKM1 | 0.291966853445788 | 7.72165619442788e-16 | Mφ Anti-inflammatory |
| ADM1 | 0.643132547916383 | 2.1325320093429e-15 | Mφ Anti-inflammatory |
| RGS12 | 0.45851075817609 | 7.15489748851792e-15 | Mφ Anti-inflammatory |
| OCIAD2 | 0.385161118648277 | 7.36822228765099e-15 | Mφ Anti-inflammatory |
| IFITM31 | 0.454254452632093 | 2.46331568009371e-14 | Mφ Anti-inflammatory |
| LSP11 | 0.465172576057808 | 7.25670156782924e-12 | Mφ Anti-inflammatory |
| PGK11 | 0.41974902872603 | 2.10337088056957e-11 | Mφ Anti-inflammatory |
| CAPZB1 | 0.35542247318131 | 5.31568737126932e-11 | Mφ Anti-inflammatory |
| AGR2 | 0.392823301980835 | 8.3259420726751e-10 | Mφ Anti-inflammatory |
| WFDC2 | 0.340668982583524 | 1.34031628875145e-08 | Mφ Anti-inflammatory |
| CD300A1 | 0.337960822879299 | 2.05888195825144e-08 | Mφ Anti-inflammatory |
| HNRNPA1 | 0.266836365582982 | 2.84708534971177e-08 | Mφ Anti-inflammatory |
| C15orf481 | 0.6901507885934 | 7.33575168729156e-08 | Mφ Anti-inflammatory |
| ATF51 | 0.374083914624863 | 7.48975720958403e-08 | Mφ Anti-inflammatory |
| CALM31 | 0.527840408236343 | 1.08440474752722e-07 | Mφ Anti-inflammatory |
| CST7 | 0.310921802705691 | 1.48236171362518e-07 | Mφ Anti-inflammatory |
| HAMP1 | 0.307679589180763 | 2.22377732472486e-07 | Mφ Anti-inflammatory |
| GIMAP7 | 0.265614092335357 | 8.05614967539522e-07 | Mφ Anti-inflammatory |
| RNASET21 | 0.426222323394167 | 3.13735242189852e-06 | Mφ Anti-inflammatory |
| HK2 | 0.349128102316227 | 4.07431390701675e-06 | Mφ Anti-inflammatory |
| IL32 | 0.830889925225703 | 0.000194672695193142 | Mφ Anti-inflammatory |
| SNHG12 | 0.289599073925511 | 0.000301824330008366 | Mφ Anti-inflammatory |
| CLEC2B | 0.315352242146439 | 0.00037514148939909 | Mφ Anti-inflammatory |
| NINJ12 | 0.331024828044675 | 0.000456713245031847 | Mφ Anti-inflammatory |
| HMOX11 | 0.700212259436928 | 0.000484573284223792 | Mφ Anti-inflammatory |
| SLC2A31 | 0.612707585723439 | 0.000869510869014924 | Mφ Anti-inflammatory |
| NKG7 | 0.462883483832166 | 0.00133101409196617 | Mφ Anti-inflammatory |
| CARD191 | 0.322957025553112 | 0.00223595294265659 | Mφ Anti-inflammatory |
| TNFRSF1B1 | 0.305443394244801 | 0.0172136703659609 | Mφ Anti-inflammatory |
| MALAT12 | 0.543289796797268 | 0.0331105220397519 | Mφ Anti-inflammatory |
| FAM162A | 0.361390589155777 | 0.0899951491533937 | Mφ Anti-inflammatory |
| ZNF385A | 0.253591103134545 | 0.090613206834508 | Mφ Anti-inflammatory |
| RAB42 | 0.320875800253603 | 0.122108150440456 | Mφ Anti-inflammatory |
| IGLC21 | 0.291827228435124 | 1 | Mφ Anti-inflammatory |
| HSPA6 | 0.763828317212568 | 1 | Mφ Anti-inflammatory |
| NGFRAP11 | 0.297217610052175 | 1 | Mφ Anti-inflammatory |
| PGAM11 | 0.324552730849323 | 1 | Mφ Anti-inflammatory |
| MT2A1 | 0.42053728926033 | 1 | Mφ Anti-inflammatory |
| SNRPD2 | 0.304909804796095 | 1 | Mφ Anti-inflammatory |
| CD142 | 0.407134461404176 | 1 | Mφ Anti-inflammatory |
| RALA1 | 0.494682089230664 | 1 | Mφ Anti-inflammatory |
| ERO1A1 | 0.416762713156602 | 1 | Mφ Anti-inflammatory |
| RGS101 | 0.294625258439478 | 1 | Mφ Anti-inflammatory |
| CCL5 | 0.755977791186388 | 1 | Mφ Anti-inflammatory |
| BCAT1 | 0.259647895681019 | 1 | Mφ Anti-inflammatory |

Supplementary Table 3: Marker genes of macrophages.
